# Brain region and epilepsy-associated differences in inflammatory mediator levels in medically refractory mesial temporal lobe epilepsy

**DOI:** 10.1186/s12974-016-0727-z

**Published:** 2016-10-13

**Authors:** Kenneth I. Strauss, Kost V. Elisevich

**Affiliations:** 1 College of Human Medicine, Michigan State University, 333 Bostwick Ave NE, Grand Rapids, MI USA; 2 Department of Clinical Neurosciences, Spectrum Health System, Grand Rapids, MI USA; 3 Division of Neurosurgery, Michigan State University, East Lansing, USA

**Keywords:** Neuroinflammation, Clinical study, Medically refractory epilepsy, Hippocampus, Entorhinal cortex, Temporal cortex, Brain cytokine levels, Brain chemokine levels, Brain vascular mediator levels

## Abstract

**Background:**

Epilepsy patients have distinct immune/inflammatory cell profiles and inflammatory mediator levels in the blood. Although the neural origin of inflammatory cells and mediators has been implied, few studies have measured these inflammatory components in the human brain itself. This study examines the brain levels of chemokines (8), cytokines (14), and vascular injury mediators (3) suspected of being altered in epilepsy.

**Methods:**

Soluble protein extracts of fresh frozen resected hippocampus, entorhinal cortex, and temporal cortex from 58 medically refractory mesial temporal lobe epilepsy subjects and 4 nonepileptic neurosurgical subjects were assayed for 25 inflammation-related mediators using ultrasensitive low-density arrays.

**Results:**

Brain mediator levels were compared between regions and between epileptic and nonepileptic cases, showing a number of regional and possible epilepsy-associated differences. Eotaxin, interferon-γ, interleukin (IL)-2, IL-4, IL-12 p70, IL-17A, tumor necrosis factor-α, and intercellular adhesion molecule (ICAM)-1 levels were highest in the hippocampus, the presumptive site of epileptogenesis. Surprisingly, IL-1β and IL-1α were lowest in the hippocampus, compared to cortical regions. In the temporal cortex, IL-1β, IL-8, and MIP-1α levels were highest, compared to the entorhinal cortex and the hippocampus.

The most pronounced epilepsy-associated differences were decreased levels of eotaxin, IL-1β, C-reactive protein, and vascular cell adhesion molecule (VCAM)-1 and increased IL-12 p70 levels. Caution must be used in interpreting these results, however, because nonepileptic subjects were emergent neurosurgical cases, not a control group.

Correlation analyses of each mediator in each brain region yielded valuable insights into the regulation of these mediator levels in the brain. Over 70 % of the associations identified were between different mediators in a single brain region, providing support for local control of mediator levels. Correlations of different mediators in different brain regions suggested more distributed control mechanisms, particularly in the hippocampus. Interestingly, only four mediators showed robust correlations between the brain regions, yet levels in three of these were significantly different between regions, indicating both global and local controls for these mediators.

**Conclusions:**

Both brain region-specific and epilepsy-associated changes in inflammation-related mediators were detected. Correlations in mediator levels within and between brain regions indicated local and global regulation, respectively. The hippocampus showed the majority of interregional associations, suggesting a focus of inflammatory control between these regions.

**Electronic supplementary material:**

The online version of this article (doi:10.1186/s12974-016-0727-z) contains supplementary material, which is available to authorized users.

## Background

Over sixty-five million people worldwide and three million people in the USA have epilepsy [1], 15–20 % of whom are medically refractory [2]. Of these, mesial temporal lobe epilepsy (mTLE) defines the majority, accounting for 60–75 % of epilepsy patients undergoing surgery [3]. No consensus has been reached regarding the mechanisms underlying epileptogenicity, although over the past decade, increasing evidence has supported the contribution of inflammatory processes in promoting its occurrence [4–6].

Little is known regarding the postulated neuroinflammatory mechanisms in epilepsy; however, inflammation-related mediators have been implicated in a number of studies. Preclinical models of epilepsy have revealed changes in a few brain inflammatory mediators [7–9]; however, only recently have clinical investigations focused on peripheral markers of inflammation [10–17]. Findings of elevated blood cytokine levels in epilepsy patients have stimulated interest in brain tissue levels of inflammation-related mediators [10, 12]. Importantly, one study showed that neurosurgical resection of a small epileptic focus significantly reduced elevated blood levels of interleukin (IL)-1β, tumor necrosis factor (TNF)-α, and macrophage inflammatory protein (MIP)-1α but not IL-6 or TGF-β that were also elevated in mTLE [11]. Thus, it is likely that the source of these mediators was, at least in part, within the central nervous system.

Cells of both peripheral (e.g., leukocytes) and brain (e.g., microglia, astrocytes, neurons) origin produce a plethora of inflammation-related mediators. Cytokines, chemokines, and certain vascular-related mediators regulate the inflammatory state locally and communicate this to other tissues. In the brain, such mediators activate microglia and astrocytes, alter cerebrovascular function, act as chemoattractants, affect the infiltration of peripheral inflammatory cells, and/or promote cellular proliferation, survival, or death. They can also affect ionic fluxes, neurotransmission, and neural cell-cell communications through a variety of mechanisms [18].

Local inflammatory responses may be an important factor in chronic epilepsies, mediating secondary tissue damage and increasing the likelihood of seizures and their recurrence. For example, in epilepsy patients, several studies have identified fluctuations in circulating inflammatory cells [13, 19–21], as well as the infiltration of peripheral blood cells within injured and epileptic brain [21–26]. Regions of neuronal death and dysfunction in epileptogenic tissues have been associated with increased leukocyte numbers and altered blood levels of inflammatory mediators [11, 13, 27–29]. Moreover, the chemokine CCL21, produced by neurons after insult or injury, can be packaged into vesicles for transport along axons; its release activates microglia via their CXCR3 receptors, which may account for remote microglial activation [30]. Areas of highly concentrated afferent input, such as the entorhinal cortex with its reciprocal connection with the hippocampus, may be particularly vulnerable under such conditions producing a site of epileptogenicity remote from the site of the original injury [31].

Inflammatory mediators, produced by either blood or brain cells, can uniquely instigate brain inflammation, with little or no effect on the extracranial environment [23]. Thus, the neuroinflammatory milieu at the site of epileptogenesis is likely related to seizure onset, recurrence, and/or resistance to antiepileptic drug (AED) treatments.

Characterization of inflammation-related mediator levels at the epileptic focus will aid in further understanding these phenomena as well as better defining the set of relevant targets for novel epilepsy therapies. Moreover, although epilepsy-associated alterations in inflammation-related mediators at the site of epileptogenesis may not be directly reflected in the systemic circulation, these changes could indirectly affect circulating inflammatory mediators and cells in such a way that permits minimally invasive detection (i.e., in the blood or cerebral spinal fluid (CSF)).

Unfortunately, there have been few investigations of *local* alterations in inflammatory mediators *within or around* the site of epileptogenesis, particularly in freshly resected human brain tissue [18]. In the present study, brain levels of 25 cytokines, chemokines, and vasoactive proteins were quantified in soluble protein extracts of fresh frozen, surgically resected temporal cortex, entorhinal cortex, and hippocampus from 58 epileptic and 4 nonepileptic patients. To our knowledge, direct, simultaneous quantification of multiple inflammation-related mediators (e.g., pro- and anti-inflammatory, immune, and vascular effectors) in resected human epileptic and nonepileptic brain tissue has not previously been accomplished.

## Methods

### Participants

This study was undertaken through an institutional review board approved process at the Henry Ford Health System (Detroit, MI, USA) where the harvest of the tissues took place between 2002 and 2008. Subsequent analyses took place at the Spectrum Health System and Michigan State University (Grand Rapids, MI, USA). Inflammation-related mediator brain levels were determined in a series of 62 neurosurgical cases (Tables 1 and 2; Additional file 1 (Patient_Data_Repository.xlsx, available online)), 58 of which were focal epilepsy patients undergoing surgical resection for medically resistant epilepsy. Four nonepileptic cases were included in the study, involving (1) intratumoral (glioblastoma) hemorrhage with herniation; (2) putaminal hypertensive hemorrhage with middle cerebral artery infarction and herniation; (3) middle cerebral artery aneurysm hemorrhage with herniation; and (4) acute subdural hematoma with temporal contusion and herniation.

**Table 1:** Study enrollee demographics by Engel classification. Enrollees were operated for mTLE by a single surgeon (KE) and followed for more than 18 months postsurgically. Individual case details can be found in the Additional file 1, available online

Total enrolled	Side resected			Gender		Mean age (Y), 39 ± 1
(*N*) 62	Right, 32	Left, 30		Female, 24	Male, 38	
Engel classification	Modified class		(*n*)	% Total		
1A	“1”		39	63 %		38 ± 1
1D	“1D”		7	11 %		45 ± 3
2A	“2”		1	2 %		37 ± 2
2B	“2”		4	6 %		
2D	“2”		1	2 %		
3A	“3”		5	8 %		41 ± 2
Unclassified	–		1	2 %		–
Nonepileptic	“Ø”		4	6 %		65 ± 3

**Table 2:** Established epilepsy risk factors and clinical variables. Individual case details can be found in the Additional file 1, available online

Epilepsy risk factor	(*n*)	Mean age	Brain side (R/L)	Family history of seizures
Closed head injury	15	40.13	7/8	3/12
Febrile	11	32.91	8/3	4/7
Infection	5	38.00	2/3	0/5
Developmental	4	42.50	0/4	1/3
Hypoxic Injury	4	37.00	1/3	1/3
Mild head injury	4	40.75	2/2	2/2
Lesional	3	32.33	2/1	0/3
Not Known	12	38.83	7/5	3/9
Nonepileptic	4	63.50	3/1	0/4

### Clinical specimens

The hippocampus, entorhinal cortex, and temporal cortex specimens were flash frozen in liquid nitrogen immediately upon resection and stored in polyethylene screw-cap tubes below −70 °C. Each specimen was homogenized at the same tissue concentration in order to improve comparisons. Brain pieces were dissected (100–200 mg, avoiding blood and white matter as much as possible) and pulverized on dry ice, transferred to pre-weighed polyethylene tubes on dry ice, weighed, and immediately sonicated in 5 vol. ice cold buffer H (100 mM potassium phosphate (pH 7.4), 10 mM ethylenediaminetetraacetic acid (EDTA), 1 mM dithiothreitol containing Cømplete^®^ Protease Inhibitor (Roche)), then centrifuged at 15K×*g* for 20 min at 4 °C. A small portion of each supernatant was assayed for total protein (microplate Bradford assay), and the remainder of the soluble protein extracts split into several aliquots that were stored frozen below −70 °C.

### Measurements

For assays, tissue extracts were thawed on ice, differentially diluted for the various VPLEX® assay sets (Additional file 2: Table S1A) in Buffer H, and assayed in duplicates. This study utilized ultrasensitive (fg/mL range) multiplex enzyme immunoassays (custom-designed nine-plex human array plates, Meso Scale Discovery (MSD), Gaithersburg, MD). The multiplex assay plates were processed according to the manufacturer’s instructions and read on the MSD Sector 6000 electrochemiluminescence plate scanner (MSD). Each VPLEX® set was accompanied by sets of mixed calibrator standards provided by the manufacturer, with seven serial dilutions in duplicates on each plate, to serve as a standard curve for the analytes measured. Prior studies (K. Strauss, unpublished data) established the appropriate dilution of protein extracts to minimize signal suppression and yield reproducible standard curves. Blanks containing the diluent only (i.e., no tissue) were also included in each study set. Analyte concentrations were extrapolated only from within the ranges for each standard curve, with coefficient of correlation, *R* ^2^ ≥ 0.98. Lower limits of detection (LLOD) and quantification (LLOQ) were determined from these curves. LLOD was the mean background from blanks plus 6 standard deviations above the mean background value. LLOQ was designated as the point on each standard curve above which the coefficient of variation (i.e., standard deviation/mean) of calibrator concentrations was less than 15 %. None of the brain extract values approached the upper limits of any assay. Values below the LLOD were assigned a value of zero. For values between the LLOQ and LLOD, a value of half the LLOQ was assigned for analysis. Mass concentrations in the original specimen were calculated by accounting for assay volume, dilution at homogenization, differential dilution at assay (depending on the VPLEX® set), and normalization to tissue wet weight (picogram analyte per gram tissue). These concentrations approximate tissue concentrations when the specific gravity of the brain tissue is close to 1 g/mL [32].

### Statistics

To determine which inflammation-related mediators might be germane to recurrent seizure disorders, two primary hypotheses were addressed in this study. These were as follows: (1) inflammatory mediator levels would be greater in brain regions proximal vs. distal to the epileptogenic focus in epilepsy cases and (2) mediators important in a recurrent seizure disorder would show different levels in epilepsy vs. nonepilepsy cases. Hypothesis (1) was tested using multiple analyses of variance (MANOVA) with brain region repeated measures, and hypothesis (2) tested using two-way ANOVA (brain region × epilepsy status). The criterion for rejecting the null hypothesis that there was no difference between group means was *p* < 0.05, unless otherwise noted.

For each mediator, outliers (on the right) were removed based upon the generalized extreme Studentized deviate test (*α* < 0.005, one-sided) [33]. In cases where there were significant main and specific effects, but no significant post hoc result (*p* > 0.05; Tukey’s honest significant difference test, the Tukey-Kramer method (Tukey’s HSD)), non-parametric analyses (i.e., Wilcoxon rank sum test or Kruskal-Wallis for multiple comparisons) were initiated to mitigate the effects of large interindividual mediator level variability. Statistical analyses were carried out using JMP 11 software (SAS Institute Inc.). Note that full analyses of the data using log_10_ transformation for normalization yielded virtually identical results to those presented below.

In the figures, individual case data are in red (epileptic) or blue (nonepileptic), the grand mean is indicated by a horizontal line, diamond center lines indicate group means, and diamond upper and lower triangles indicate the 95 % confidence interval.

Secondary analyses compared the mediator levels in all three tissues with each secondary independent variable using two-way ANOVA (brain region × secondary variable). These variables included the following: age at surgery (years); side of the brain (L/R); gender (M/F); epilepsy duration (years); Engel classification (modified to a five-point scale including nonepileptic, 1A, 1D, 2A–C, 3A,B); epilepsy risk factor (nonepileptic, lesional, moderate-to-severe closed head injury (CHI), mild CHI, developmental, febrile, or infection); and pathology/imaging reports (hippocampus-only abnormalities, cortex-only abnormalities, or both).

To determine whether mediator values were linearly correlated, levels in each brain region were compared using multivariate analysis. Due to the large number of simultaneous comparisons (i.e., 25 mediators in three brain regions gives 2775 pairwise comparisons), a high degree of type I error was expected. To achieve acceptable rates of type I and type II errors, a more stringent type I error (*α*′ = 0.0001, *df* = 73, *r* ≥ 0.4340) was used to select significant correlations; however, all significant comparisons with correlation coefficient 99.9 % confidence intervals that overlapped zero were eliminated.

## Results

Soluble protein extracts from fresh frozen surgically resected human hippocampus, entorhinal cortex, and temporal cortex specimens from 58 medically refractory epilepsy and 4 nonepileptic neurosurgical cases (Tables 1 and 2) were assayed for 25 inflammation-related mediators using ultrasensitive multiplex enzyme immunoassay technology (see “Methods” section, Additional file 2:﻿ Figures S2A ﻿- S2AA). The primary independent variables for the study were the brain region and preoperative epilepsy status.

### Variability in mediator measurements

Median mediator values varied over 4 orders of magnitude, and interindividual variability was great, even after outlier removal (Additional file 2: Table S3B). Variability was examined using coefficients of variation (CV) calculated for each mediator, between the brain regions and by the epilepsy status (Additional file 2: Tables S3C–E). The grand mean CV for all mediators was 125 %, with tissue means of 114 % in the hippocampus, 130 % in the entorhinal cortex, and 132 % in the temporal cortex. The highest variability was seen with VEGF (236 %), IL-2 (232 %), IL-6 (195 %), IL-8 (195 %), and IL-10 (183 %) levels. Interestingly, IL-2 and IL-10 had high variability across all tissues independent of epilepsy status, whereas VEGF, IL-6, and IL-8 showed greater variability in epileptic compared to nonepileptic tissues (Additional file 2: Tables S3D, E).

### Brain mediator levels

Levels of 23 inflammation-related mediators were detectable in the majority of specimens. The exceptions were granulocyte-macrophage colony-stimulating factor (GM-CSF) (74 % tested negative; see Additional file 2: Tables S3F, G) and IFN-γ (94 % of specimens tested negative).

Most of the brain tissue examined did not exhibit detectable GM-CSF (see Table 3﻿ note 4, Additional file 2: Figure S2H); however, GM-CSF was present in the hippocampus (15/48 epilepsy and 0/3 nonepileptic cases), entorhinal cortex (3/58 epilepsy and 1/4 nonepileptic cases), and temporal cortex (22/55 epilepsy and 3/4 nonepileptic cases). Entorhinal cortical GM-CSF levels were lower overall (Table 3; *p* < 0.005 Tukey’s HSD). This was further supported by examining the number of nonzero GM-CSF values in each brain region (Additional file 2: Table S3G(A–C)). There was significant nonhomogeneity between the brain regions (*p* < 0.0001 chi-square, *df* = 2), with entorhinal cortex showing the lowest incidence of GM-CSF-positive cases. In addition, nonhomogeneity was shown between the brain region and epilepsy status groups (*p* < 0.001 chi-square, *df* = 5) (Additional file 2: Figure S2H, Table S3G(C)).

**Table 3:** Inflammation-related mediators by brain region

Mediator^a^ (pg/g tissue)	Hippocampus			Entorhinal cortex			Temporal cortex			MANOVA, brain region *P* value^b^	Two-way ANOVA, epilepsy (±) × region *P* value^c^
	Mean	SEM	*N*	Mean	SEM	*N*	Mean	SEM	*N*		
Eotaxin	627****	67	51	404	40	62	307	42	59	*<0.0001*	*<0.0001*
IP-10	3597**	453	51	6213	1292	59	7004	1413	59	0.0306	*0.0038*
MCP-1	8051	1199	51	4551*	465	60	8440	1803	58	*0.0045*	0.1923
MCP-4	839	89	51	545*	35	62	676	152	57	*0.0011*	0.3751
MIP-1α	1008	189	51	590	124	62	2187****	361	59	*0.0001*	<0.0001
MIP-1β	2362	311	51	1636	208	61	3306***	437	59	*0.0037*	0.0153
TARC	666	111	51	501	49	61	855	205	59	0.1047	0.3991
GM-CSF^d^	3062	710	51	470****	230	62	2579	400	59	*<0.0001*	0.0009
IFN-γ^d^	33.1****	10.9	51	0	0	62	1.56	1.56	59	*0.0143*	0.0008
IL-1α	992**	162	51	3616**	372	62	2576**	271	58	*<0.0001*	<0.0001
IL-1β	13.8**	2.2	51	27.8**	3.9	62	39.7**	5.0	57	*<0.0001*	*0.0002*
IL-2^d^	38.3****	7.9	49	4.16	1.83	62	7.97	2.27	59	*<0.0001*	*<0.0001*
IL-4	11.7****	1.2	49	7.17	0.40	62	6.45	0.44	59	*0.0010*	<0.0001
IL-6	33.4	8.2	51	34.8	8.5	60	60.8	15.2	57	0.1409	*0.0161*
IL-8	570	121	51	933	251	61	1473*	331	57	*0.0029*	0.0259
IL-10^d^	4.21	0.75	51	1.49**	0.47	61	3.37	0.85	59	*0.0225*	0.1399
IL-12/23 p40	72.9	14.8	51	97.9	12.8	61	88.2	13.3	58	0.1490	0.1273
IL-12 p70	5.17	0.68	50	2.65***	0.43	62	3.59	0.29	58	*0.0494*	*0.0019*
IL-17A	949****	112	49	378	44	62	252	28	59	*<0.0001*	<0.0001
TNF-α	5.89	0.75	51	3.16	0.67	60	5.26	0.85	57	0.1176	0.0518
TNF-β	3.85	0.89	49	8.52****	0.58	62	4.90	0.56	59	*0.0143*	<0.0001
VEGF	7716***	1865	49	3553	1199	62	1889	720	59	*0.0231*	0.0465
CRP	26,540	2222	49	22,069	1435	62	15,624****	1115	59	*<0.0001*	*<0.0001*
ICAM-1	12,088**	1110	50	9243	786	62	8291	897	58	*0.0035*	*<0.0001*
VCAM-1	21,887	4754	51	21,351	3659	62	14,427	2632	59	0.0103	*<0.0001*

*Abbreviations*: *Eotaxin* (also known as (aka) CCL-11), *IP-10* interferon gamma-induced protein 10 (aka CXCL-10), *MCP-1* monocyte chemoattractant protein 1 (aka CCL-2), *MCP-4* monocyte chemoattractant protein 4 (aka CCL-13), *MIP-1α* macrophage inflammatory protein 1-alpha (aka CCL-3), *MIP-1β* macrophage inflammatory protein 1-beta (aka CCL-4), *TARC* thymus- and activation-regulated chemokine (aka CCL-17), *GM-CSF* granulocyte-macrophage colony-stimulating factor (aka CSF2), *IFN-γ* interferon gamma, *IL* interleukin, *IL-8* (aka CXCL-8), *TNF* tumor necrosis factor, *VEGF* vascular endothelial growth factor, *CRP* C-reactive protein, *ICAM-1* intercellular adhesion molecule 1 (aka CD54) , *VCAM-1* vascular cell adhesion molecule 1 (aka CD106)

*p ≤ 0.01, Wilcoxon/Kruskal-Wallis;

**p < 0.05, Tukey's HSD;

***p < 0.01, Tukey’s HSD;

****p < 0.001, Tukey’s HSD

^a^Mediator values in pg/g tissue, *N* is number of observations, SEM = (standard deviation/*N* ^1/2^). The total number of specimens assayed was as follows: hippocampus = 51, entorhinal cortex = 62, temporal cortex = 59; differences from these values indicate outlier removals. Case data, distributions, and summary statistics can be found in Additional file 2: Figures S2A - S2AA, and the complete data set in Additional file 1

^b^Repeated measures MANOVA of mediator levels by brain region, brain region effect. Italicized *P* values indicate significant post hoc comparisons

^c^Two-way ANOVA epilepsy status (±) × brain region, main effect. See Table 4 for post hoc comparisons

^d^GM-CSF and IFN-γ exhibited 74 and 94 % assay values of zero (below the lower limit of detection), and assays for IL-2 and IL-10 had 65 and 59 % zero values, with more than half the zero values in the cortical region specimens

Similarly, most of the brain tissues examined did not exhibit detectable IFN-γ levels (see Table 3, Additional file 2: Figure S2U, Table S3H(A–C)) and no epilepsy-related differences could be discerned. However, IFN-γ was more often detected in the hippocampus (10/51 cases) than in the entorhinal cortex (0/62) or temporal cortex (1/59) (*p* = 0.0001 chi-square, *df* = 2). As with GM-CSF, there was nonhomogeneity between groups with respect to the brain region and epilepsy status (*p* = 0.004 chi-square, *df* = 5).

#### Chemokines

**Fig. 1:**
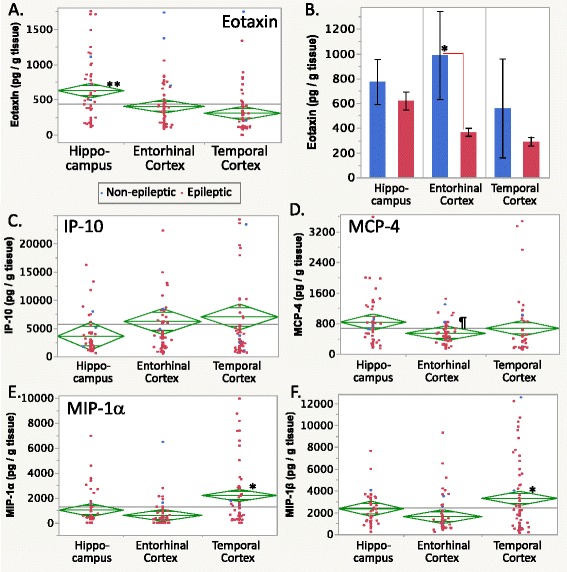
**a** Eotaxin levels in human brain specimens. Overall, the hippocampal levels were greater than the entorhinal and temporal cortex. **b** Epilepsy-related eotaxin differences. Brain levels of eotaxin were greater among nonepileptic cases overall, and the nonepileptic entorhinal cortex eotaxin was greater than in the epileptic entorhinal cortex. **c** IP-10 levels were not significantly different between brain regions. **d** MCP-4 levels appeared lower in the entorhinal cortex than the other tissues. **e** MIP-1α and **f** MIP-1β levels in the human brain showed similar regional differences. In all graphs, *blue* = nonepileptic cases, *red* = epileptic cases. Data points are means of duplicate measurements; *diamond graphs* show the grand mean for all cases as a *horizontal line*, *diamond center line* indicates group mean, *upper and lower triangles* indicate the 95 % confidence intervals. *Histograms* show group mean ± standard error; *solid lines* show within epilepsy status, and *dashed lines* show between epilepsy status group differences. Post hoc testing: **p < 0.001; *p < 0.01; ^§^ *p* < 0.05, Tukey’s HSD; ^¶^ *p* < 0.01, non-parametric Wilcoxon/Kruskal-Wallis, unless otherwise noted

Eotaxin levels (Table 3) showed brain region﻿-specific differences over all cases using brain region repeated measures (MANOVA; *p* < 0.0001). Hippocampal eotaxin levels were higher than those in the entorhinal and temporal cortices (Fig. 1a, *p* < 0.001 post hoc Tukey’s HSD). Epilepsy-related differences in eotaxin were also apparent. Eotaxin levels were higher in nonepileptic cases (Table 4, Fig. 1b) and, specifically in the entorhinal cortex using epilepsy × brain region repeated measures (MANOVA; epilepsy effect, *p* < 0.01 Tukey’s HSD).

**Table 4:** Epilepsy-related differences in inflammation-related mediator levels

Mediator (pg/g tissue)	Brain region					
	Hippocampus		Entorhinal cortex		Temporal cortex	
	Nonepileptic	Epileptic	Nonepileptic	Epileptic	Nonepileptic	Epileptic
Eotaxin	772 ± 180^a,^**	618 ± 71	986 ± 355^a,^**	364 ± 31	558 ± 401^a,^**	289 ± 36
*n*	3	48	4	58	4	55
IP-10^c^	4.96 ± 1.79	3.51 ± 0.47	24.0 ± 17.4^b,^**	5.26 ± 0.97	7.68 ± 5.27	6.96 ± 1.48
*n*	3	48	3	56	4	55
MIP-1α^d^	698 ± 494	1027 ± 199	2192 ± 1463	480 ± 80	873 ± 437	2283 ± 383
*n*	3	48	4	58	4	55
IL-1β^d^	29.1 ± 15.3^a,^**	12.9 ± 2.1	59.0 ± 26.4^a,^**	25.7 ± 3.7	49.3 ± 39.2^a,^**	39.0 ± 4.8
*n*	3	48	4	58	4	53
IL-2	24.6 ± 24.6	39.2 ± 8.3	11.3 ± 6.7	3.67 ± 1.90	30.1 ± 30.1^b,^***	6.37 ± 1.25
*n*	3	46	4	58	4	55
IL-6^d^	149 ± 38^a,^****	26.1 ± 7.2	65.2 ± 47.6	33.2 ± 8.6	61.7 ± 35.2	60.7 ± 16.0
*n*	3	48	3	57	3	54
IL-12 p70	2.26 ± 2.26	5.35 ± 0.71^a,^**	1.94 ± 1.94	2.69 ± 0.44^a,^**	0	3.79 ± 0.28^a,^**
*n*	3	47	4	58	3	55
TNF-α^d^	5.04 ± 3.21	5.95 ± 0.77	7.60 ± 4.44	2.93 ± 0.67	0.79 ± 0.79	5.51 ± 0.88
*n*	3	48	3	57	3	54
CRP^c^	50.5 ± 37.4^a,^****	25.5 ± 18.8	39.7 ± 11.7^a,^****	20.9 ± 12.1	23.8 ± 7.9^a,^****	15.0 ± 1.0
*n*	2	47	4	58	4	55
ICAM-1^c^	16.0 ± 8.7	11.9 ± 1.1	19.1 ± 8.1^b,^****	8.57 ± 0.59	6.60 ± 2.26	8.42 ± 0.95
*n*	2	48	4	58	4	54
VCAM-1^c^	131 ± 9^b,^****	15.0 ± 2.9	75.7 ± 22.5^b,^****	17.6 ± 3.1	49.9 ± 16.1^b,^****	11.9 ± 2.3
*n*	3	48	4	58	4	55

**p < 0.05;

***p < 0.01;

****p < 0.001, post hoc Tukey’s HSD

^a^Epilepsy effect; overall epileptic vs. nonepileptic cases. Only the greater set of values are marked

^b^Epilepsy × brain region interactions, epileptic vs. nonepileptic cases in individual brain region(s). Only the greater set of values are marked

^c^The units of these mediators are in ng/g tissue

^d^These mediators were elevated in the blood of mTLE patients [11]; neurosurgical resection reduced the blood levels of TNF-α, IL-1β, and MIP-1α, but not IL-6

Levels of IP-10 showed no regional differences (Fig. 1c), likely due to the high variance in cortical specimens. Although IP-10 levels were greater in nonepileptic cases overall, and specifically in the entorhinal cortex, when considering the epilepsy effect and epilepsy × brain region interaction (both *p* < 0.001 Tukey’s HSD), these effects may have been due to a few extreme nonoutlier values in the cortical specimens (see Additional file 2: Figure S2B).

**Fig. 2:**
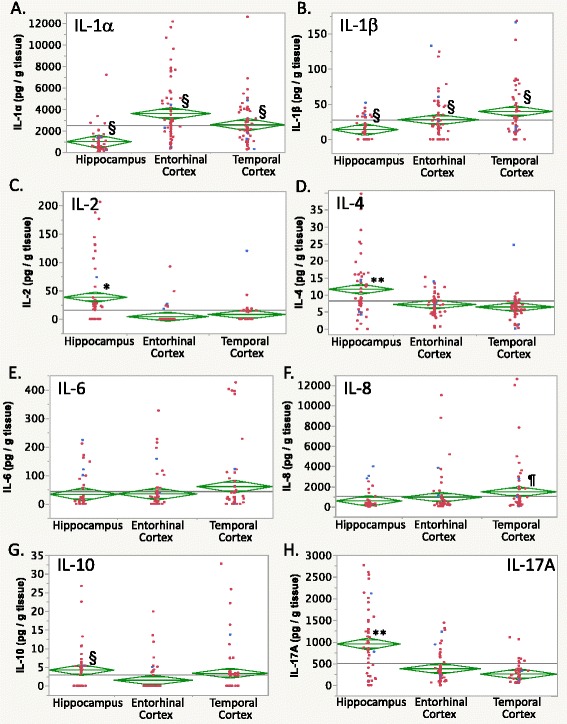
Brain region differences in interleukin (IL) levels. **a** IL-1α and **b** IL-1β levels differed between all three brain regions. **c** IL-2 and (**d**) IL-4 levels were elevated in the hippocampus. (**e**) IL-6 levels showed high variability without brain region-specific or epilepsy-related differences. **f** IL-8 levels were higher in the temporal cortex than the other brain regions, whereas **g** IL-10 levels were higher in the hippocampus than in the entorhinal cortex, and **h** IL-17A levels were higher in the hippocampus than the entorhinal cortex and temporal cortex. For graphics details, see Fig. 1

Monocyte chemoattractant protein (MCP)-1 and MCP-4 levels had brain region-specific but no epilepsy-related differences (Table 3). Nonparametric analyses suggested that entorhinal cortex levels were lower for each than in the hippocampus or temporal cortex (post hoc Wilcoxon/Kruskal-Wallis, *p* = 0.002 and *p* = 0.0005, respectively). Inspection of the individual case data showed that this was true in 67 % of cases for MCP-1 (Additional file 2: Figure S2C) and in 53 % of cases for MCP-4 levels (Fig. 1d, Additional file 2: Figure S2D).

MIP-1α levels showed brain region-specific differences overall (Fig. 1e, Table 3). Temporal cortical MIP-1α levels were greater than in the entorhinal cortex or hippocampus (*p* < 0.01 Tukey’s HSD). MIP-1β levels showed similar region-specific differences overall (Fig. 1f, brain region effect *p* = 0.0037). The temporal cortical MIP-1β levels were higher than in the entorhinal cortex (Table 3, *p* = 0.002 Tukey’s HSD) but not different from the hippocampal levels. No epilepsy-related MIP-1α or MIP-1β differences were detected.

Brain levels of the thymus- and activation-regulated chemokine (TARC) showed neither brain region- nor epilepsy-related changes. The temporal cortex from epileptic cases had very high TARC levels; however, this was obscured by a high variability of TARC levels in the epileptic temporal cortex (Additional file 2: Figure S2G, Table S3E).

#### Cytokines

Interleukin (IL-)1α levels showed graded brain region-specific differences overall (Fig. 2a, Table 3) with greatest expression in the entorhinal cortex followed by that in the temporal cortex and, lastly, the hippocampus (*p* < 0.03 post hoc Tukey’s HSD). No epilepsy-related differences were discerned. On the other hand, IL-1β levels (Fig. 2b, Table 3) showed both graded brain region-specific differences and epilepsy-related effects. IL-1β levels were greatest in the temporal cortex, intermediary in the entorhinal cortex, and lowest in the hippocampus (Table 3; *p* < 0.05 Tukey’s HSD). In addition, nonepileptic brain regions showed greater IL-1β levels than epileptic brain regions overall (Table 4; epilepsy × brain region ANOVA, epilepsy effect *p* < 0.04, *p* < 0.05 Tukey’s HSD).

IL-2 (Fig. 2c) and IL-4 (Fig. 2d) brain levels showed similar patterns of regional differences. IL-2 was elevated in the hippocampus compared to either the entorhinal or temporal cortices (Table 3, *p* < 0.005 Tukey’s HSD). A significant epilepsy status × brain region repeated measures interaction (*p* < 0.03) was noted, with IL-2 levels in the nonepileptic temporal cortex exceeding those in the epileptic temporal cortex (*p* < 0.01 Tukey’s HSD). Similarly, hippocampal IL-4 levels were elevated compared to other tissues (Table 3, *p* < 0.001 Tukey’s HSD), but no epilepsy-related differences were observed.

IL-6 levels were numerically highest in the temporal cortex but variability in both epileptic and nonepileptic cases confounded the analysis (Additional file 2: Figure S2M, Tables S3C–E, H). Although there were no overall differences in IL-6 between brain regions (Fig. 2e), there was a significant epilepsy × repeated measures brain region interaction (*p* < 0.02). The hippocampal IL-6 levels were greater in nonepileptic compared to epileptic cases (Table 4, *p* < 0.001 Tukey’s HSD).

Overall, IL-8 levels in the temporal cortex were greater than in the hippocampus or entorhinal cortex (Table 3, Fig. 2f, *p* < 0.008 post hoc Wilcoxon/Kruskal-Wallis), with the entorhinal cortical levels intermediary to these regions (not significantly different (n.s.d.)). In contrast, IL-10 levels were higher in the hippocampus than in the entorhinal cortex (Fig. 2g, *p* < 0.05 Tukey’s HSD), with intermediary levels (n.s.d.) found in the temporal cortex.

IL-17A levels showed brain region-specific differences, with the hippocampal levels higher than both the entorhinal and temporal cortices (Table 3, *p* < 0.001 Tukey’s HSD). Neither epilepsy-related differences nor brain region × epilepsy interactions were detected for brain IL-17A levels.

Bioactive IL-12 is a p35:p40 heterodimer-designated IL-12 p70. The p40 subunit also binds with other proteins, such as p17, to form IL-23. Two IL-12-related assays were performed, the IL-12/23 p40 assay that detected p40 in all its forms *except* for IL-12 p70, and the IL-12 p70 assay which detected only intact heterodimers (Paul Grulich, MSD Scientific Support Group, personal communication).

**Fig. 3:**
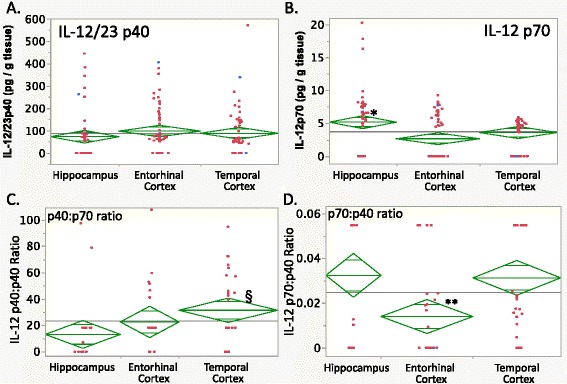
IL-12 brain levels. **a** IL-12/23 p40 levels did not show brain region-specific differences. **b** The heterodimeric IL-12 p70 showed brain region-specific differences, with higher levels in the hippocampus than in the entorhinal cortex. **c** The molar ratio of IL-12/23 p40 to IL-12 p70 (p40:p70) was elevated in the temporal cortex compared with the hippocampus and entorhinal cortex (^§^ *p* < 0.02, Tukey’s HSD). **d** IL-12 p70:p40 molar ratios were depressed in the entorhinal cortex (compared to the hippocampus and temporal cortex, **p ≤ 0.01 Tukey’s HSD), despite higher p70 levels in the hippocampal specimens. For graphics details, see Fig. 1

Though IL-12/23 p40 levels showed neither brain region- nor epilepsy-related differences (Fig. 3a), the heterodimer IL-12 p70 exhibited both brain region-specific and epilepsy-related differences. IL-12 p70 was increased in the hippocampus compared to the entorhinal cortex (Fig. 3b, *p* < 0.005 Tukey’s HSD). A bimodal distribution might be present for IL-12 p70 but could not be confirmed because most measurable values were close to the lower limit of quantification. Epileptic brain had higher levels of IL-12 p70 than nonepileptic cases (Table 4, epilepsy effect *p* < 0.03, *p* < 0.05 Tukey’s HSD).

Brain levels of IL-12/23 p40 were much greater than that of IL-12 p70 (Fig. 3a–d, Additional file 2: Table S3B). Mean p40 to p70 molar ratios (Fig. 3c, Additional file 2: Figure S2AA) were 13 ± 4 in the hippocampus, 23 ± 5 in the entorhinal cortex, and 31 ± 6 in the temporal cortex, suggesting that only a small proportion of the total p40 tissue content was in IL-12 p70. In addition, the mean p40:p70 molar ratio was higher in the temporal cortex than in the hippocampus (Fig. 3c, repeated measures MANOVA *p* = 0.0271, *p* < 0.02 Tukey’s HSD). Moreover, the IL-12 p70:p40 molar ratio showed lower values in the entorhinal cortex compared with other tissues (Fig. 3d, Additional file 2: Figure S2Z, repeated measures MANOVA, brain region effect *p* = 0.0007, *p* ≤ 0.01 Tukey’s HSD). Both of the latter findings were consistent with elevated hippocampal IL-12 p70 levels (Fig. 3b).

**Fig. 4:**
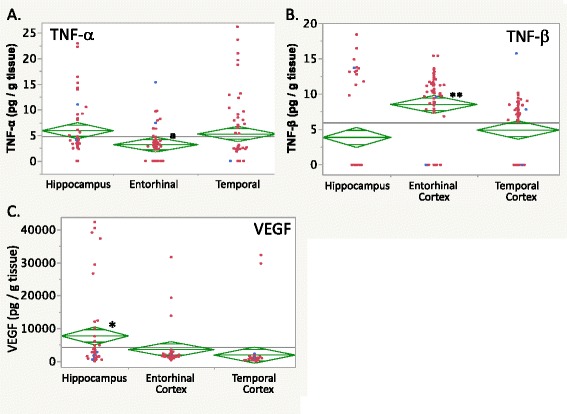
**a** TNF-α levels in the human brain did not show region-specific differences by post hoc testing. However, on a case-by-case analysis (*a*), the entorhinal cortex TNF-α levels were lower than in the hippocampus or temporal cortex for 59 % of all individuals. **b** TNF-β brain levels were higher in the entorhinal cortex, and higher than in the hippocampus or temporal cortex for 80 % of all individual cases. **c** VEGF levels were greater in the hippocampus than in the entorhinal or temporal cortex. For graphics details, see Fig. 1

TNF-α showed no brain region-specific or epilepsy-related differences (Table 3). Nonetheless, case data showed that the entorhinal cortical levels were lower than those in the hippocampus and temporal cortex in most individuals (69 and 52 %, respectively, Additional file 2: Figure S2S). In contrast, TNF-β brain levels were highest in the entorhinal cortex overall (Fig. 4b, *p* < 0.001 Tukey’s HSD). Data showed the entorhinal cortical levels to be higher than in the hippocampus and temporal cortex in 76 and 83 % of cases, respectively (Additional file 2: Figure S2T).

#### Vascular mediators

Hippocampal VEGF levels were two- and fourfold greater than in the entorhinal and temporal cortical regions, respectively (Table 3, Fig. 4c, *p* < 0.01 Tukey’s HSD). Nonparametric analyses suggested graded VEGF levels, with the hippocampus showing the highest levels followed by the entorhinal cortex, and lastly, the temporal cortex (*p* < 0.0002 Wilcoxon/Kruskal-Wallis). No epilepsy-related differences were observed; however, there was increased VEGF variation in epileptic tissue specimens (Additional file 2: Figure S2V, top right) that may have obscured differences between epileptic and nonepileptic VEGF levels (Additional file 2: Tables S3C, E, I).

**Fig. 5:**
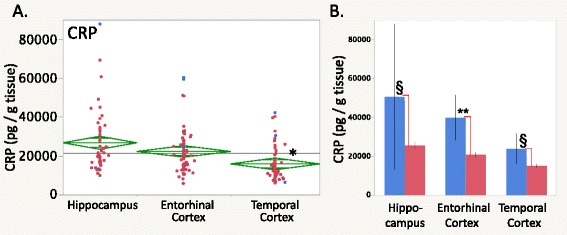
**a** CRP (C-reactive protein) levels were the lowest in the temporal cortex compared to the hippocampus and entorhinal cortex. **b** CRP levels were higher in the nonepileptic hippocampus and entorhinal cortex. For graphics details, see Fig. 1

Brain levels of C-related protein (CRP) were lower in the temporal cortex than in the hippocampus or entorhinal cortex (Fig. 5a, *p* < 0.01 Tukey’s HSD). Moreover, two-way ANOVA showed 50, 53, and 63 % lower CRP levels in the epileptic hippocampus, entorhinal cortex, and temporal cortex, respectively (Fig. 5b, Table 4, epilepsy effect *p* < 0.0001, all *p* values <0.05 Tukey’s HSD).

**Fig. 6:**
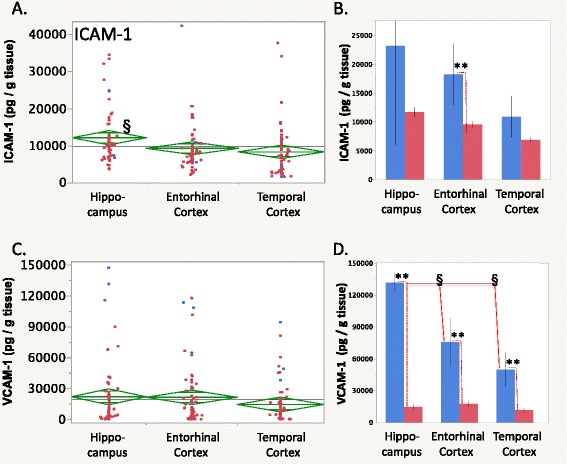
**a**, **b** ICAM-1 and (**c**, **d**) VCAM-1 levels in the human brain. **a** ICAM-1 levels were higher in the hippocampus than in the entorhinal or temporal cortex (brain region repeated measures MANOVA *p* < 0.0007, *p* <0.03 Tukey’s HSD). **b** In nonepileptic cases, the entorhinal cortex ICAM-1 levels were higher than in the epileptic cases (epilepsy × brain region MANOVA *p* < 0.0007, *p* < 0.001 Tukey’s HSD). **c** VCAM-1 levels showed no brain region-specific differences over all cases. **d** VCAM-1 levels were higher in nonepileptic cases in all three regions (*dashed lines*, epilepsy × brain region interaction *p* < 0.0001). In the nonepileptic hippocampus, VCAM-1 levels were elevated compared with other brain regions (*solid lines*, *p* < 0.05 Tukey’s HSD). For graphics details, see Fig. 1. **p < 0.001, ^§^ *p* < 0.05, post hoc Tukey’s HSD

Intercellular adhesion molecule (ICAM)-1 levels were highest in the hippocampus compared to the cortical regions overall (Table 3, Fig. 6a, *p* < 0.03 Tukey’s HSD). While there was no significant epilepsy-related effect, there were significant epilepsy × brain region repeated measures interactions (*p* = 0.0008). ICAM-1 levels in the nonepileptic entorhinal cortex were higher than in the epileptic cases (Fig. 6b, *p* < 0.001 Tukey’s HSD).

Vascular cell adhesion molecule (VCAM)-1 levels did not appear different across brain regions overall (Fig. 6c) but showed multiple effects among the nonepileptic cases (Table 4, epilepsy effect *p* < 0.0001, brain region effect *p* < 0.0001, epilepsy × brain region interaction *p* < 0.0001). Specifically, VCAM-1 levels were greater in each of the nonepileptic brain region cases compared to the epileptic cases (Fig. 6d, *p* < 0.001 Tukey’s HSD). Also, VCAM-1 levels in the nonepileptic hippocampus were greater than in either the nonepileptic entorhinal cortex or temporal cortex (Fig. 6d, Table 4, *p* < 0.05 Tukey’s HSD).

### Secondary analyses

Secondary independent variables were also considered in the analyses of these brain mediator levels. Differences based upon gender, side of the brain resected, epilepsy duration, age at surgery, presumptive epilepsy risk factors, surgical electrode placement prior to resection (“phase 2”), and Engel outcome classifications were evaluated. Moreover, pathology and imaging findings were also investigated as potential covariates.

#### Gender, side of brain, and epilepsy duration

None of the mediator levels differed with respect to patient gender, side of the brain affected, or time since seizure onset.

#### Age at surgery (years)

Age at surgery was a significant predictor only for the hippocampal VCAM-1 (linear regression, *R* ^2^ = 0.340, *p* < 0.02) and IL-8 (*R* ^2^ = 0.294, *p* < 0.05), both increasing with age (Additional file 2: Figure S4A).

These findings brought into question the observed brain region- and epilepsy-related differences. For VCAM-1, a difference of 26 years in the mean age between epileptic and nonepileptic groups yielded a difference of 39,650 pg/g tissue in the hippocampal VCAM-1 levels, according to the predicted VCAM-1 vs. age fit line (Additional file 2: Figure S4A). Nevertheless, the difference in the hippocampal VCAM-1 levels between nonepileptic and epileptic cases was over 200 % of this value (Table 4, Fig. 6d). Brain region × epilepsy × age interactions were significant (three-way ANOVA main effect *p* <0.0001, triple interaction *p* = 0.013). Thus, age- and epilepsy-related differences were confirmed for the hippocampal VCAM-1 levels. Interestingly, although no other brain region showed a significant correlation between VCAM-1 and age, the entorhinal cortex exhibited ~150 % and the temporal cortex ~95 % of the predicted differences between nonepileptic and epileptic cases according to the hippocampal VCAM-1 vs. age fit line.

The findings for IL-8 differences were also supportive of epilepsy-related differences in the hippocampus. Whereas the nonepileptic hippocampal IL-8 levels were 300 % for those expected by age alone (according to the predicted IL-8 vs. age fit line, Additional file 2: Figure S4A), the entorhinal and temporal cortical levels were closer to predicted age-related levels (88 and −53 %, respectively). Finally, analyses of epilepsy status × age for each brain region showed significant effects for IL-8 levels only in the hippocampus (main effect *p* < 0.0001, epilepsy effect *p* = 0.0002, age effect *p* = 0.554, interaction *p* = 0.642).

#### Epilepsy risk factors

Previously identified epilepsy risk factor(s), presumed to have contributed to patients' recurrent seizures (Table 2), were analyzed to find associations with inflammation-related mediators in the brain. Other than the expected brain region differences (brain region × epilepsy risk factor ANOVAs), several risk factor associations appeared significant.

Multivariate screening analyses revealed that high VEGF levels in the hippocampus were associated with mild closed head injury (mCHI); elevated IP-10 and IL-12/23 p40 levels in the entorhinal cortex were associated with the infection risk factor (Additional file 2: Figure S4B). No temporal cortical mediator differences showed significant associations with any presumptive epilepsy risk factor(s). Univariate analyses suggested several other associations. Overall IL-1α levels were higher in the infection subgroup compared to CHI or febrile risk factor subgroups; overall IL-6 levels were higher in the CHI subgroup compared to the febrile subgroup; and overall MIP-1β levels were higher in the mCHI subgroup compared to the developmental, infection, febrile, or lesional risk factor subgroups (Additional file 2: Figure S4B).

#### Phase 2 electrode placement

Several associations emerged between the mediators assayed and prior placement of intracranial surface electrodes for localization of epileptogenic foci. Cases that had electrode placement prior to resection (*n* = 31) exhibited higher levels of eotaxin, IL-1α, MCP-1, MCP-4, CRP, and ICAM-1 over all brain regions combined and, specifically, in both cortical regions (Additional file 2: Table S4I). There were no mediator level differences noted in the hippocampus, which might suggest that electrode placement had minimal effect on nonadjacent brain structures.

#### Engel classification

**Fig. 7:**
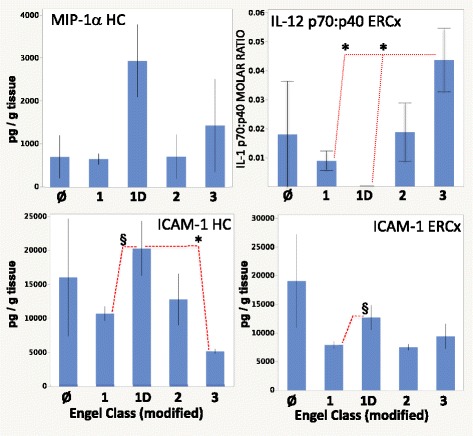
Differences in selected mediator levels correlated with a modified Engel outcome scale. Engel classifications were simplified to 1A, 1D, “2,” and “3” (see Table 1, classifications 1A, 1D, 2A - 2D and 3A, respectively). The nonepileptic group is represented by “Ø”. *HC* hippocampus, *ERCx* entorhinal cortex, *TCx* temporal cortex. *Dashed lines* show epileptic subgroup differences, **p < 0.001, *p ≤ 0.01, ^§^ *p* < 0.05 Tukey’s HSD vs. subgroups indicated

Using a modified Engel classification score (Table 1), several mediators appeared to be associated with Engel outcome subgroups. The majority of associations observed were between the nonepileptic group and the epileptic subgroups; these differences were not described here because they were reported above. Such differences may reflect less on outcome after surgery and more on neurophysiologic differences between nonepileptic and epileptic cases. There were, nonetheless, several instances of differences between Engel subgroups (Fig. 7, brain region × modified Engel main effects *p* ≤ 0.002). Hippocampal MIP-1α and ICAM-1 levels were greater in the Engel - 1D subgroup than those in the Engel-1A subgroup (Fig. 7, top row; Engel effect *p* = 0.0060 and *p* = 0.0412, respectively, *p* ≤ 0.02 Tukey’s HSD). The same is held for ICAM-1 in the entorhinal cortex (brain region × modified Engel interaction *p* = 0.0213, *p* < 0.05 Tukey’s HSD). In the temporal cortex, MCP-4 levels were increased in the Engel-2 subgroup compared with those in better outcome Engel-1A and -1D subgroups (Fig. 7, bottom row, left; brain region × modified Engel interaction *p* = 0.0091, *p* < 0.01 and *p* < 0.04 Tukey’s HSD, respectively). In addition, the molar ratio of IL-12 p70 (bioactive IL-12) to its p40 subunit was increased in the worst outcome (Engel-3) subgroup, compared to those in both the Engel-1A and -1D subgroups (Fig. 7, bottom row, right; brain region × modified Engel interaction *p* = 0.0280, *p* < 0.05 Tukey’s HSD).

#### Pathology and imaging reports

Of the 55 cases with pathology and imaging report data, 84 % had observed hippocampal pathology, with two thirds of these showing hippocampal sclerosis. Of the 55 cases, 44 % showed cortical pathology. Interestingly, 56 % had hippocampal with no cortical findings, but only 15 % had cortical with no hippocampal findings.

An analysis was performed to interrogate differences of inflammatory-related mediator levels with respect to neuropathological findings (hippocampus only, cortex only, or both). The phase 2 status was considered salient to this analysis because half the cases underwent prior surface electrode placement. Phase 2 status was taken into account using two-way ANOVAs. Both pathology and MRI reports were tested separately with the following results. Cases in which both the hippocampal and cortical abnormalities were reported by the pathologist showed significantly (*p* < 0.05 Tukey’s HSD) increased levels of MIP-1α (hippocampus), IL-17A (temporal cortex), and ICAM-1 (temporal cortex). MIP-1α and ICAM-1 results were complicated by significant interaction between neuropathologic findings and phase 2 status (phase 2^+^ cases had higher levels). The IL-17A finding appeared more robust, as MR imaging showing abnormalities in both regions was also associated with increased IL-17A levels in the temporal cortex. In addition, MR imaging abnormalities in both regions were associated with elevated levels of TARC (entorhinal cortex) and IP-10 (temporal cortex).

Cases in which only the hippocampal abnormalities were reported by the pathologist were associated with increased IL-1α levels (entorhinal cortex), and a trend (*p* ≈ 0.05) was noted in the MR imaging-evident hippocampus-only analysis as well. Thus, abnormal findings in both the cortex and hippocampus may be associated with increased proinflammatory mediator levels such as IL-17A and IP-10 in the temporal cortex or IL-1α and TARC in the entorhinal cortex. Involvement of the hippocampus only may be associated with increased IL-1α levels in the entorhinal cortex. Intriguingly, no mediator differences were observed in cases with pathology or MR abnormalities in the cortex only.

### Mediator-to-mediator correlations

Another approach to understanding brain inflammatory regulation is to identify groups of mediators with associated expression profiles. Robust mediator-to-mediator associations were observed in 141 pairwise comparisons across the hippocampus, entorhinal cortex, and temporal cortex (*p* < 0.0001, see “Methods” section). These correlations (Additional file 2: Figure S4C) were distributed rather evenly, with each brain region having approximately one third of the associations. Of the 141, 70 % were between different mediators within a single brain region, 24 % were between different mediators in different brain regions, and 6 % were individual mediators between different brain regions. Only two negative correlations were observed between region-specific mediators. The hippocampal CRP and IL-12 p70 levels were inversely associated, and the entorhinal cortex TNF-α levels were inversely associated with hippocampal TNF-β levels.

Of the correlations of different mediators within a single brain region, half were in the temporal cortex and one quarter each were in the hippocampus and entorhinal cortex. For correlations of different mediators in different regions, 57 % were between the hippocampus and temporal cortex, 36 % were between the hippocampus and entorhinal cortex, and 7 % were between the entorhinal and temporal cortices. These proportions may suggest differential control of inflammation-related mediator levels within/between these regions.

Individual mediator levels that correlated between different brain regions were as follows: VCAM-1 levels correlated between all three brain regions; MCP-1, IL-1α, IL-2, and VEGF levels correlated between the entorhinal and temporal cortices; ICAM-1 levels between the hippocampus and entorhinal cortex; as well as IP-10 levels between the hippocampus and temporal cortex. These interregional associations indicate predominantly extrinsic (i.e., cerebral or systemic) control of these brain mediator levels.

Interestingly, of the 141 mediator-to-mediator correlations observed in these three brain regions, the majority (78 %) were accounted for by eight mediators, namely, eotaxin (22), ICAM-1 (17), MCP-1 (16), IL-4 (14), IL-6 (14), IL-8 (11), MIP-1α (9), and VCAM-1 (8). This observation implies a hierarchical dynamic in which mediators with multiple correlations may regulate, directly or indirectly, the levels of other mediators.

Conversely, there were several mediators that had three or fewer correlations with mediators in any other tissue (Additional file 2: Figure S4C, bottom). These included GM-CSF and IFN-γ, for which most tissue values were below the level of detection, IL-1α and VEGF which were self-correlated between the temporal and entorhinal cortices, as well as TNF-β. Moreover, in specific brain regions, a few mediators had zero or one correlation with other mediators.

### Blood mediator levels vs. brain tissue content

Although usually considered intravascular mediators, cytokines and chemokines in CSF and, by inference, in the central nervous system (CNS) may be more concentrated than those found in the systemic circulation. For example, during the first 10 days after severe head trauma, patient CSF levels of IL-6 and TNF-α (but not CRP) were greater than in the blood [34]. Also, at least in lupus patients, MCP-1 and GM-CSF were present at much higher levels in neural tissue than in the serum [35]. In epilepsy patients, there were clear transients of leukocyte and mediator changes immediately postictally [19] leaving open the question as to whether there were transient changes in the brain or at the site(s) of epileptogenesis.

In order to rule out brain mediator content in the present study being entirely due to blood levels, brain levels were compared with published blood levels (Additional file 2: Table S4J–K) and potential differences were identified. Not only were median MCP-1 levels in the brain greater than in peripheral blood, but IP-10, MIP-1β, IL-1α, IL-8, IL-17A, VEGF, CRP, and ICAM-1 levels appeared to exceed maximal blood levels by two- to sixfold or more. In contrast, Yoshio et al. [35] found no differences between serum and CSF levels for IP-10 and IL-8 and greater levels in serum than CSF for MIP-1β, IL-17A, and VEGF (IL-1α, CRP, and ICAM-1 were not assayed). These comparisons would seem to reveal the existence of neural-based degradation mechanisms for these mediators.

## Discussion

### Mediator levels

Human epilepsy disorders exhibit peripheral cytokine changes that may originate at sites of neural dysfunction and contribute to seizure propagation (reviewed in [12]). Sinha et al. [15] analyzed serum IL-1β, IL-2, IL-4, IL-6, IFN-γ, or TNF-α detectability in 100 postictal patients and 100 healthy controls. None of the age- and sex-matched control subjects had detectable levels of any of these cytokines, whereas among epilepsy patients, the overall incidence was 74 %. Of the nine epilepsy patients who had a lumbar puncture, CSF levels of these cytokines were detectable in subject(s) with no serum levels. Significantly, none of the subjects who were serum-positive for a cytokine were CSF-negative for that cytokine. Serum IL-6, IL-1β, TNF-α, and MIP-1α levels were elevated in AED-resistant epilepsy patients and, in patients undergoing resection of the epileptogenic region, IL-1β, TNF-α, and MIP-1α levels decreased after 8 weeks postoperatively [11]. These results implied that the epileptogenic brain tissue removed was either a direct or indirect source of these mediators. Until now, there has been a paucity of data on human brain tissue inflammation-related mediator levels [10].

In the current study, however, none of these mediators appeared to be elevated proximal to the site of epileptogenesis. In fact, IL-6, IL-1β, and MIP-1α levels were higher in the temporal cortex than in the hippocampus, and TNF-α showed no regional or epilepsy-related differences, though levels in entorhinal cortex may have been lower than either the hippocampus or temporal cortex (see Additional file 2: Figure S2S). If both studies are valid, and the patient samples represent the greater population, then it must be concluded that the resected tissue had been an indirect stimulus for production of these mediators in the blood.

Proinflammatory mediator levels were hypothesized to be higher in the hippocampus at the site of epileptogenesis. This was true for eotaxin, IFN-γ, IL-2, IL-4, IL-12 p70, IL-17A, TNF-α, and ICAM-1. Interestingly, Sinha et al. [15] found that blood levels of most of these were more detectable in epilepsy patients than in age-matched controls. Eotaxin (CCL11) recruits eosinophils by activating their CCR3 receptors. CCL11 transport across the blood-brain barrier (BBB) resulted in region-specific alterations of eotaxin brain levels [36] and age-related increases in humans have been implicated in cognitive decline in a novel mouse model [37]. IFN-γ, an important activator of macrophages and inducer of major histocompatibility class II immune/inflammatory activities, is associated with a number of autoinflammatory and autoimmune diseases. Its brain levels were virtually undetectable except in 10/49 hippocampus specimens exclusively from epileptic cases. IL-17A, also highest in the hippocampus, has synergistic effects with IFN-γ, IL-1, and TNF-α, acting as a proinflammatory mediator not unlike IFN-γ [38]. TNF-α levels were among the lowest measured in this study (Additional file 2: Table S3B). Some variability in the convulsive effect of TNF-α has been noted previously and its relatively reduced levels in the entorhinal cortex may attest to this variability. The proconvulsive effect may be concentration-dependent, as with its role in *Shigella dysenteriae*-related seizures at low concentrations and an anticonvulsive role at high concentrations [39]. Lower picomolar concentrations may preferentially affect the p55 receptor pathway, increasing synaptic activity [40], and promoting epileptogenicity in the longer term. ICAM-1 signaling is proinflammatory via recruitment of macrophages and leukocytes across the BBB [22, 41]. In a mouse model of mTLE, ICAM-1 levels were locally induced in the hippocampus [22].

Neurosurgical resection of the epileptic focus significantly reduced elevated blood levels of IL-1β, TNF-α, and MIP-1α in mTLE patients [11]. TNF-α levels were highest in the hippocampus, consistent with the implications of prior findings. However, brain levels of IL-1β and MIP-1α were lower in the hippocampus compared to the temporal cortex. Moreover, none of these mediators showed any epilepsy-associated increases, as might have been predicted from that study [11].

MIP-1α, IL-β, and IL-8 levels were highest in the temporal cortex. MIP-1α belongs to the C–C chemokine family (CCL3) and is involved in the recruitment and activation of macrophages, monocytes, and neutrophils. IL-8 (CXCL8) functions in inflammatory cell chemotaxis and phagocytosis, as well as angiogenesis. IL-8 has also been postulated, through NF-kB- and TNF-α-related mechanisms, to contribute to local inflammatory mechanisms in response to oxidative insults [42]. IL-β and IL-α showed graded levels with cortical levels exceeding those in the hippocampus. IL-1β is involved in a plethora of inflammatory activities, including the induction of many other proinflammatory mediators; the induction of cyclooxygenase-2 (PTGS2) by this cytokine in the CNS is just one response to inflammation. IL-1β secreted by hypoxic astrocytes upregulated MCP-1 and ICAM-1 levels that are thought to play a crucial role in leukocyte recruitment [43]. IL-α also mediates numerous inflammation-related activities, including TNF induction. Both are acute-phase cytokines that operate in the picomolar-femtomolar range. MIP-1β, which acts as a chemoattractant for natural killer cells, monocytes, and other immune cells, was also greater in the temporal cortex than in the entorhinal cortex.

Intriguingly, CRP levels were lower in the temporal cortex than in the hippocampus or entorhinal cortex. As a well-documented inflammatory biomarker, its levels follow those of proinflammatory cytokines; this may reflect an increased inflammatory load proximal to the site(s) of epileptogenesis. Paradoxically, IL-10, an anti-inflammatory cytokine, was found at higher levels in the hippocampus than in the entorhinal cortex but not significantly different from temporal cortical levels.

In the entorhinal cortex, IL-α and TNF-β levels were highest, compared to the hippocampus and temporal cortex. TNF-β (﻿aka﻿﻿ lymphotoxin alpha) is a highly inducible, cell surface molecule that mediates a variety of inflammatory responses, as well as apoptotic cell death.

Findings of neuroanatomic mediator level differences suggest brain-specific regulation; however, these might be confounded by blood levels, with brain tissue levels reflecting only differences in vascularization. If true, it could be expected that more vascularized brain tissue would regularly demonstrate higher mediator levels, but this was not the case. Moreover, a review of the recent literature on blood and CSF inflammatory mediator levels indicated, at least for many mediators, that individual CSF levels were greater than blood levels [35, 44, 45]. In comparing brain tissue levels in this study with consensus blood levels from multiple studies (Additional file 2: Table S4J, K), median brain levels exceeded median blood levels for IP-10 (~threefold), MCP-1 (~fourfold), MIP-1β (~threefold), IL-8 (~fivefold), IL-17A (~fourfold), VEGF (~threefold), CRP (>tenfold), and ICAM-1 (~sixfold). Thus, the neuroanatomic differences observed were likely due to brain-related regulatory mechanisms rather than contamination from the blood or CSF.

#### Epilepsy-related differences

Differences in inflammation-related mediators between epileptic and nonepileptic subjects were further hypothesized to suggest some significance for AED-resistant epileptogenicity. Caution must be taken when comparing these epilepsy cases with nonepilepsy cases as the nonepileptic subjects were (i) emergent neurosurgical patients and not healthy controls, (ii) comprised only four cases, and (iii) had an age distribution (64.5 ± 6.3) different from that of the epileptic group (38.5 ± 1.5)(nonetheless, there was no instance where an epilepsy-related difference could be fully explained by the age difference). Moreover, no data were available regarding circulating AED levels (or other medications) at the time of surgery for these cases, so confounding influences on mediator levels due to drug effects cannot be ruled out.

Eotaxin levels were lower in epilepsy specimens overall and particularly in the entorhinal cortex. Interestingly, phase 2 patients appeared to have higher eotaxin levels than cases without prior electrode placement. Higher levels in the hippocampus would suggest eotaxin is involved in both acute (nonepileptic) and chronic (epileptic) neuroinflammation. Although IP-10 levels were also reduced in the entorhinal cortex of epilepsy cases, this finding is suspect due to several high nonoutlier values in the nonepileptic group.

Proinflammatory cytokines IL-1β and IL-6 were higher in the nonepileptic cases. Both are acutely upregulated, so it is likely the emergent conditions warranting neurosurgical intervention contributed to the elevations. It is interesting to note that, for both mediators, the primary region of increase was in the hippocampus, suggesting that local control of these cytokines dominated, perhaps in the early phase of neuroinflammation.

Epileptic brain exhibited higher levels of IL-12 p70 overall; most of the nonepileptic specimens had no measurable levels. IL-12 p70 was also elevated in the cerebral cortex of pediatric epilepsy patients, compared to nonepileptic controls [46]. The ratio of bioactive IL-12 p70 (p40:p35 heterodimer) to its p40 subunit appeared elevated both in the hippocampus and temporal cortex, indicating a more active IL-12 in these regions relative to the entorhinal cortex. IL-12/23 p40 levels were much greater than IL-12 p70 levels (Additional file 2: Table S3B), indicating that the manifestation of IL-12 bioactivity would be more dependent upon p35 subunit availability and/or the rate of heterodimer formation. In the periphery, IL-12 p70 stimulates T cell proliferation and differentiation, as well as natural killer cell activation. It also promotes induction of IFN-γ and TNF-α in T cells, through which it may block the formation of new blood vessels. Elevated brain IL-12 p70 in epileptics may be associated with transient leukocyte changes observed postictally, manifested by increased lymphocytes, neutrophils, NK cells, and NK-like T cells, with decreased T cells and CD4^+^/CD8^+^ ratios [19]. These peripheral changes were all resolved by 24 h postictally. Elevated IL-12 p70 may also relate to alterations in specific blood cytokines and leukocyte numbers in adult epileptics that were differentially ameliorated by various AEDs [13].

Vascular mediators CRP, ICAM-1, and VCAM-1 all showed epilepsy-related differences. Epileptic CRP levels were about half those of nonepileptics in the hippocampus and temporal cortex and 63 % in the entorhinal cortex. This was likely due to the emergent medical condition(s) of the nonepileptic cases. Unexpectedly, epileptic ICAM-1 levels in the entorhinal cortex were about half those of nonepileptic cases. Interestingly, blood levels of ICAM-5, a related gene located predominantly on neural cells, were ~fivefold lower in a group of epileptic patients than in age-matched controls [47]. ICAMs are endothelial-, leukocyte-, and tissue-associated proteins important in cell-cell adhesion and leukocyte extravasation [41, 48]. In this study, ICAM-1 levels were highest in the hippocampus, particularly among the epilepsy cases. Conversely, VCAM-1 levels were much lower in the epileptic cases across all three brain regions (11 % in the hippocampus, 23 % in the entorhinal cortex, 24 % in the temporal cortex). Moreover, the regional VCAM-1 differences noted in the nonepileptic cases were not apparent in the larger group of epileptic cases. While nonepileptic cases comprised a very small, nonage-matched sample, this might have implications for epilepsy-related changes in hippocampal VCAM-1. Not much is known about the contribution of human VCAM-1 to neuroinflammation, though its levels increased acutely in the blood and CSF after brain injuries [49, 50]. Again, the emergent nature of the nonepileptic cases might explain these differences.

Though the epilepsy-related differences will require further investigation to corroborate, several other notable findings were derived from these results. Distinct regional levels of inflammation-related mediators likely have significance in neuroinflammatory physiology. For example, correlations of mediator levels within and between individual brain regions reveal the influences of intrinsic and extrinsic controls on neuroinflammatory regulation. These are summarized below.

#### Mediator-to-mediator correlations

Of the inflammation-related mediators with significant correlation(s), 70 % were with other mediators within a single brain region. Intraregional correlations support the notion of local control of multiple mediators, whereas correlations between different brain regions support more external mechanisms. Half of the observed correlations within a single brain region were in the temporal cortex, suggesting greater local regulatory mechanisms, compared to those of the hippocampus and entorhinal cortex.

Correlations of different mediators in different regions suggested more external control mechanisms (e.g., by diffusible factors or distributed neural cell inputs). Over 90 % of these types of correlations were between the hippocampus and the cortical brain regions (temporal cortex, 57 %; entorhinal cortex, 36 %). Only 7 % of interregional correlations of different mediators were between the entorhinal and temporal cortices. Although it cannot be known from these data in which direction controls may be exerted, the preponderance of hippocampal involvement along with their neuroanatomical relationships suggest a focus of hippocampal control of inflammation-related mediator levels between these regions.

Only IL-1α, IP-10, MCP-1, IL-2, VEGF, ICAM-1, and VCAM-1 levels were correlated between different brain regions. Of these, except for VCAM-1, tissue level differences were observed. IL-1α was greatest in the entorhinal cortex and diminished from the temporal cortex to the hippocampus; IP-10 was also lowest in the hippocampus, whereas MCP-1 was greater in the hippocampus and temporal cortex than in the entorhinal cortex; and IL-2, VEGF, and ICAM-1 were higher in the hippocampus than in both cortical regions. Thus, individual mediators showing tissue level differences *and* interregional correlations suggest the existence of common, external regulatory mechanisms with local gain controls.

### Functional implications

Systemic inflammatory cells, as well as activated intrinsic neuroinflammatory cells, likely contribute to neurochemical and neurophysiologic dysfunction in the affected brain regions. Inflammatory mediators produced by both types of cells can uniquely affect the brain, with little or no manifestation in the periphery [23]. It has been proposed that glial cells establish a cytokine/chemokine network in the ischemic brain—“activated microglia produce… various types of cytokines… which activate astrocytes to synthesize chemokines…. Chemokines in turn activate and/or recruit microglial cells in the injured site” [43]. Cytokines IL-1 and TNF-α, as well as chemokines MCP-1, RANTES, and IL-8 were implicated in these networks [43].

Interestingly, the majority (110/141) of mediator-to-mediator associations observed in this study were accounted for by only eight mediators, namely, eotaxin (22), ICAM-1 (17), MCP-1 (16), IL-4 (14), IL-6 (14), IL-8 (11), MIP-1α (9), and VCAM-1 (8). This observation implies a hierarchical dynamic in which mediators with multiple correlations may regulate, directly or indirectly, the levels of other mediators. These could be one basis of neuroinflammatory functional networks.

## Conclusions

Both brain region-specific and epilepsy-associated differences in inflammation-related mediator levels were detected. The hippocampus had the majority of regional increases. Correlations in mediator levels within and between brain regions indicated local and global regulation, respectively. The hippocampus showed the majority of interregional associations, suggesting a focus of inflammatory control between these regions. Thus, it would be important to further characterize the range of inflammation-related mediator levels in the normal brain and in neurologic disorders like epilepsy, with recognized inflammatory components. Finally, further characterization of differences between epilepsies and patient responses to medications would clarify whether AED-resistant seizures resulted from underlying differences in the inflammatory status of the affected brain regions.

## Additional files

Additional file 1: Patient data repository. (XLSX 79 kb)
Additional file 2: Supplemental data. (PDF 5924 kb)

## Abbreviations

AED: Antiepileptic drug
ANOVA: Analysis of variance
BBB: Blood-brain barrier
CHI: Moderate-to-severe closed head injury
CNS: Central nervous system
CRP: C-reactive protein
CSF: Cerebral spinal fluid
EDTA: Ethylenediaminetetraacetic acid
ERCx: Entorhinal cortex
GM-CSF: Granulocyte-macrophage colony-stimulating factor
HC: Hippocampus
ICAM: Intercellular adhesion molecule
IFN: Interferon
IL: Interleukin
IP-10: Interferon gamma-induced protein 10
LLOD: Lower limit of detection
LLOQ: Lower limit of quantification
MANOVA: Multiple analyses of variance
mCHI: Mild closed head injury
MCP: Monocyte chemoattractant protein
MIP: Macrophage inflammatory protein
MSD: Meso Scale Discovery Corp.
mTLE: Mesial temporal lobe epilepsy
n.s.d.: Not significantly different
SEM: Standard error of the mean
TARC: Thymus- and activation-regulated chemokine
TNF: Tumor necrosis factor
Tukey’s HSD: Tukey’s honest significant difference test, the Tukey-Kramer method
VCAM: Vascular cell adhesion molecule
VEGF: Vascular endothelial growth factor
