# Supplementary material for: Brain region and epilepsy-associated differences in inflammatory mediator levels in medically refractory mesial temporal lobe epilepsy
Source: J Neuroinflammation. 2016 Oct 13;13:270. doi: 10.1186/s12974-016-0727-z (PMC5064886; doi:10.1186/s12974-016-0727-z)
Supplement: Additional file 2: — Supplemental data. (PDF 5924 kb) [file 12974_2016_727_MOESM2_ESM.pdf]

**SUPPLEMENTAL DATA for “Brain region and epilepsy-associated differences in inflammatory mediator levels in medically refractory epilepsy”**

Kenneth I. Strauss, Ph.D., College of Human Medicine, Michigan State University

Kost Elisevich, M.D., Ph.D., Department of Clinical Neurosciences, Spectrum Health System, Grand Rapids, Michigan; Division of Neurosurgery, Michigan State University

## SUPPLEMENTAL DATA

Individual case details can be found in the supplemental data file **Patient_Data_Repository.xlsx**, available online.

**S1. Custom VPLEX Assays**

**Table S1A.** Assay Analytes and Tissue Dilutions

**S2. Mediator Case Data and Distributions (Figures 2A – 2AA):** Human hippocampus, entorhinal and temporal cortex inflammation-related mediators

**S3. Mediator Levels Variability**

**Table S3B**. Brain Inflammation-related Mediator Relative Tissue Concentrations

**Table S3C**. Mediator Levels Overall Coefficients of Variation

**Table S3D**. Mediator Coefficients of Variation by Tissues

**Table S3E**. Mediator Coefficients of Variation by Epilepsy Status

**Table S3F**. Mediator Coefficients of Variation by Tissues and Epilepsy Status

**Table S3G**. GM-CSF Measurements Homogeneity Analyses

**Table S3H**. IFN-γ Measurements Homogeneity Analyses

**S4. Miscellaneous Supplemental Material**

**Table S4I.** Phase-2 and Brain Inflammation-related Mediator Associations

**Table S4J-K.** Inflammation-related Mediator Brain & Consensus Blood Levels
Comparisons of median brain and estimated blood levels of inflammation-related mediators (table of citations referenced in **Table S4K**)

**Figure 4A. Age-Related Correlations**: VCAM-1 and IL-8

**Figure 4B. Epilepsy Risk Factor Associations**

**Figure 4C. Relational Correlations of Mediators and Between Tissues**
Highly correlated inflammation-related mediator levels in human hippocampus, entorhinal and temporal cortices

## 1. Custom VPLEX Assays

**Table S1A. Assay Analytes and Tissue Dilutions**

| Human Chemokine Panel 1 1:10 dilution |
|---|
| Human Eotaxin |
| Human IP-10 |
| Human MCP-1 |
| Human MCP-4 |
| Human MIP-1α |
| Human MIP-1β |
| Human TARC |
| **Human Cytokine Panel 1 1:6 dilution** |
| Human GM-CSF |
| Human IL-12/23 p40 |
| Human IL-17A |
| Human IL-1α |
| Human TNF-β |
| Human VEGF |

| Human Proinflammatory Panel 1 1:3 dilution |
|---|
| Human IFN-γ |
| Human IL-10 |
| Human IL-12p70 |
| Human IL-1β |
| Human IL-2 |
| Human IL-4 |
| Human IL-6 |
| Human IL-8 |
| Human TNF-α |
| **Human Vascular Injury 1:4 dilution** |
| Human CRP |
| Human ICAM-1 |
| Human VCAM-1 |

Each multiplex assay plate was prepared as recommended by the manufacturer (MSD). Brain soluble protein extracts were diluted (see above), enough for the four VPLEX plates in duplicate, in Buffer H containing Cømplete protease inhibitors and 0.2% fraction V bovine serum albumin, and added to the appropriate wells. Assays were carried out according to manufacturer’s instructions and results read on the Sector 6000 electroluminescence plate reader (MSD).

**2. Mediator Case Data and Distributions: Human hippocampus, entorhinal and temporal cortex inflammatory-related mediators (Figures 2A – 2AA)**

***Left top***: Summary statistics on analyte assay data (pg / g tissue). All case values included.

***Right top***: Nonepileptic (blue) and epileptic (red) specimen data (pg / g tissue). All values shown, except where a single outlier is more than 5-fold greater than the overall mean. Outlier(s) were removed based upon multivariate analysis Jacknife distance ($\alpha$ = 0.005). Differences between the number of observations in the text and supplemental data indicate outlier removal.

***Left Bottom***: Region-specific data (pg / g tissue) showing group mean at the diamonds' center line, upper and lower triangles indicate 95% confidence interval. The grand mean for all data (excluding outliers) is indicated by a horizontal line on each graph.

***Right Bottom***: Case data by region, showing tissue levels (pg / g tissue) for each case, are presented to show possible trends across brain regions. A single case, 32 (nonepileptic), exhibited unusually high values in multiple mediators and all three brain regions. Many of the values for this case had already been removed during the outlier analysis phase of data processing.

# A. Eotaxin

## Hippocampus

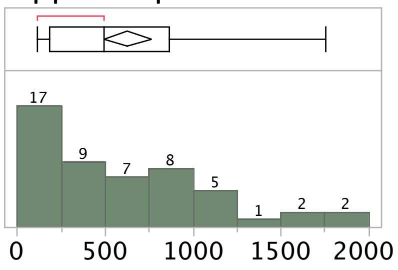


| Summary Statistics | |
|---|---|
| Mean | 627.39695 |
| Std Dev | 479.60428 |
| Std Err Mean | 67.158032 |
| Upper 99.5% Mean | 824.63768 |
| Lower 99.5% Mean | 430.15623 |
| N | 51 |
| Median | 495.28437 |

## Entorhinal Cortex

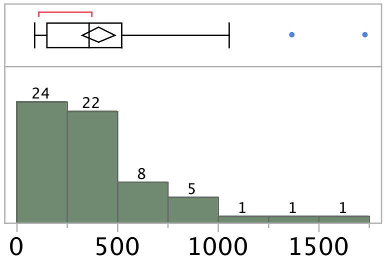


| Summary Statistics | |
|---|---|
| Mean | 404.03588 |
| Std Dev | 316.47896 |
| Std Err Mean | 40.192869 |
| Upper 99.5% Mean | 521.10682 |
| Lower 99.5% Mean | 286.96493 |
| N | 62 |
| Median | 356.17724 |

## Temporal Cortex

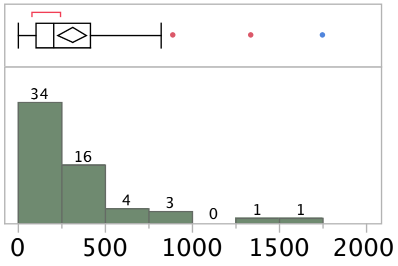


| Summary Statistics | |
|---|---|
| Mean | 306.71176 |
| Std Dev | 322.81264 |
| Std Err Mean | 42.026626 |
| Upper 99.5% Mean | 429.36203 |
| Lower 99.5% Mean | 184.06149 |
| N | 59 |
| Median | 203.0078 |

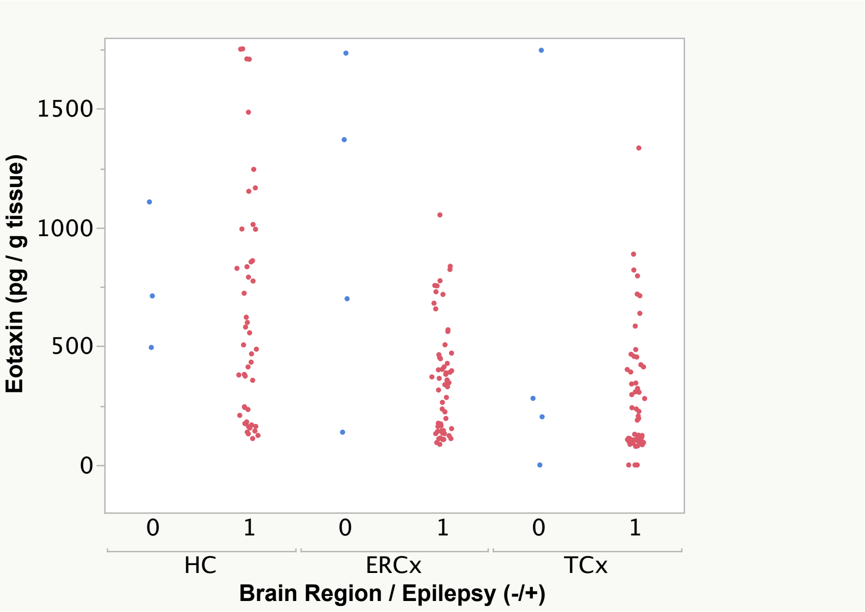


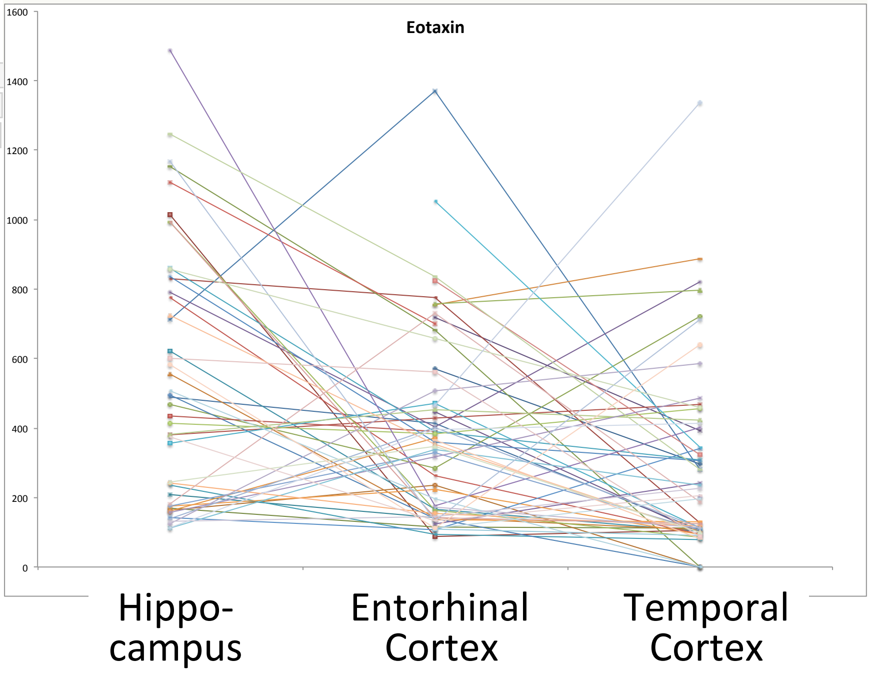

# B. IP-10

## Hippocampus

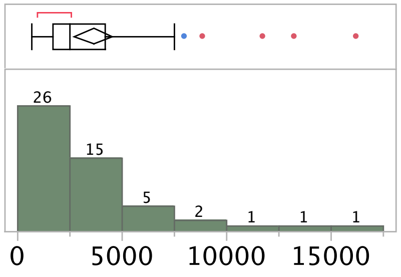


| Summary Statistics | |
|---|---|
| Mean | 3597.3037 |
| Std Dev | 3233.4141 |
| Std Err Mean | 452.76854 |
| Upper 99.5% Mean | 4927.0686 |
| Lower 99.5% Mean | 2267.5388 |
| N | 51 |
| Median | 2453.7824 |

## Entorhinal Cortex

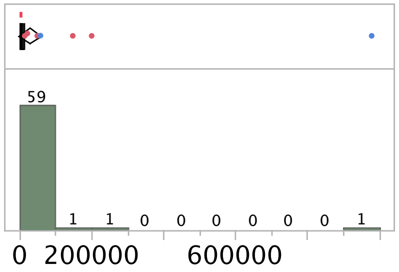


| Summary Statistics | |
|---|---|
| Mean | 27346.426 |
| Std Dev | 126966.83 |
| Std Err Mean | 16124.803 |
| Upper 99.5% Mean | 74313.613 |
| Lower 99.5% Mean | −19620.76 |
| N | 62 |
| Median | 3661.8556 |

## Temporal Cortex

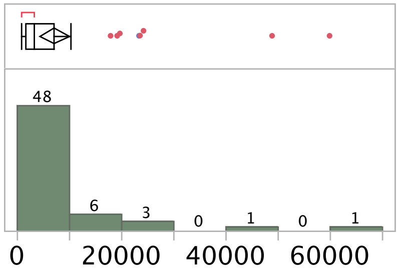


| Summary Statistics | |
|---|---|
| Mean | 7004.3431 |
| Std Dev | 10854.599 |
| Std Err Mean | 1413.1484 |
| Upper 99.5% Mean | 11128.468 |
| Lower 99.5% Mean | 2880.2186 |
| N | 59 |
| Median | 3029.3689 |

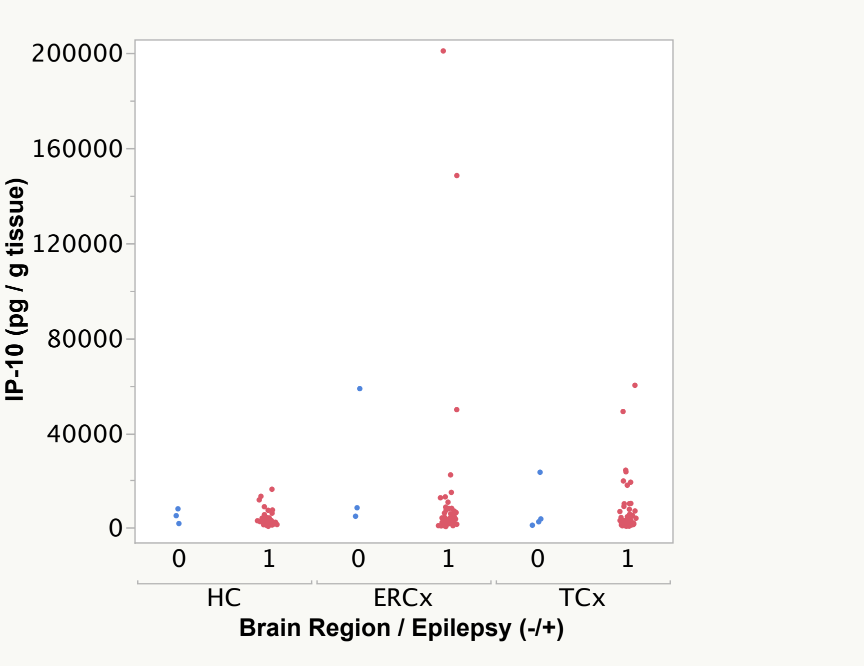


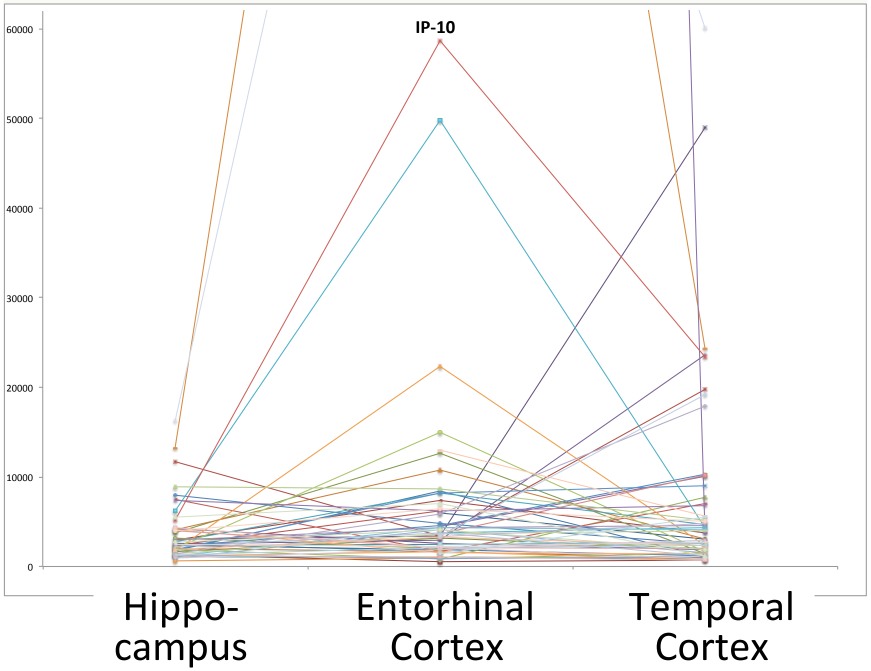

# C. MCP-1

## Hippocampus

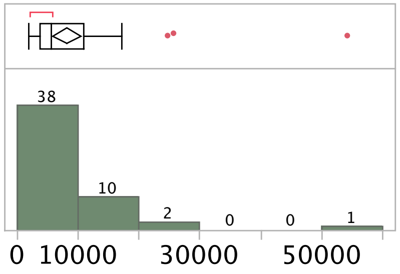


| Summary Statistics | |
|---|---|
| Mean | 8051.2261 |
| Std Dev | 8565.9279 |
| Std Err Mean | 1199.4698 |
| Upper 99.5% Mean | 11574.026 |
| Lower 99.5% Mean | 4528.4263 |
| N | 51 |
| Median | 5371.3781 |

## Entorhinal Cortex

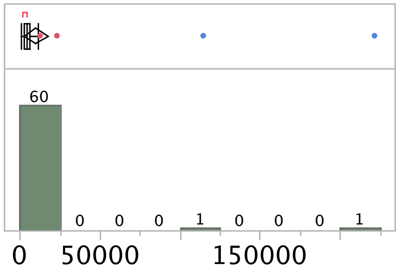


| Summary Statistics | |
|---|---|
| Mean | 9828.9269 |
| Std Dev | 30919.751 |
| Std Err Mean | 3926.8124 |
| Upper 99.5% Mean | 21266.668 |
| Lower 99.5% Mean | −1608.815 |
| N | 62 |
| Median | 3840.2336 |

## Temporal Cortex

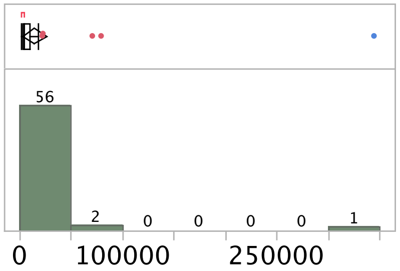


| Summary Statistics | |
|---|---|
| Mean | 14138.823 |
| Std Dev | 45838.378 |
| Std Err Mean | 5967.6485 |
| Upper 99.5% Mean | 31554.775 |
| Lower 99.5% Mean | −3277.129 |
| N | 59 |
| Median | 4424.941 |

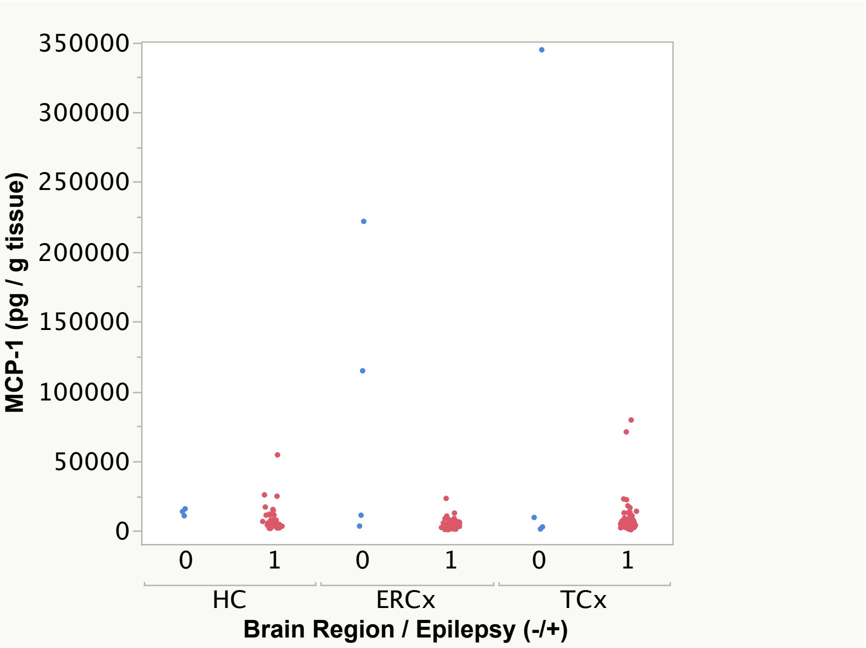


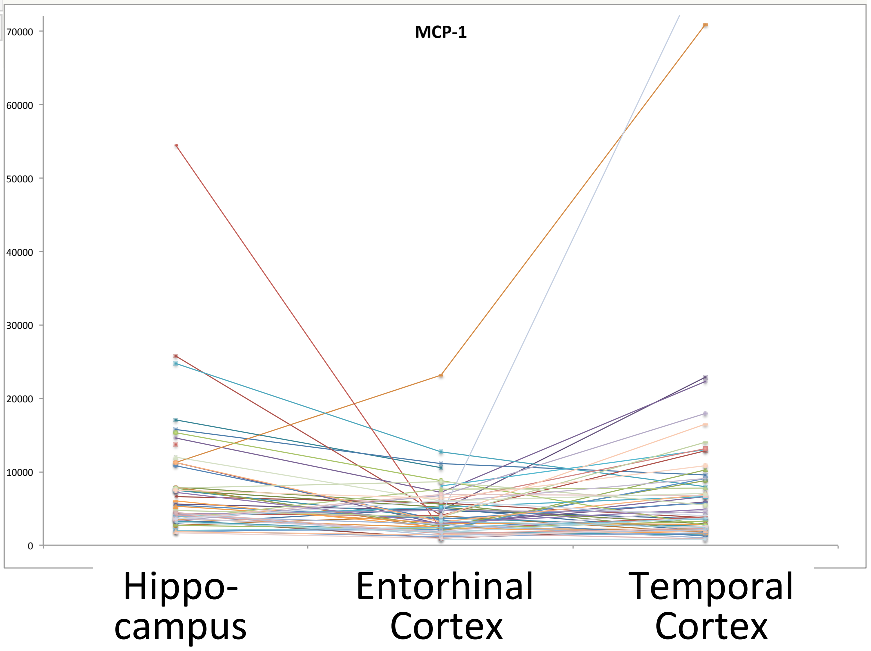


Individual cases where ERCx is lowest

| ERCx < HC | ERCx < TCx | **Total** |
|---|---|---|
| 37 | 21 | |
| 73% | 36% | **53%** |

# D. MCP-4

## Hippocampus

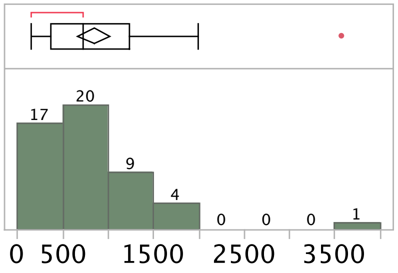


| Summary Statistics | |
|---|---|
| Mean | 839.44975 |
| Std Dev | 637.46631 |
| Std Err Mean | 89.263138 |
| Upper 99.5% Mean | 1101.6124 |
| Lower 99.5% Mean | 577.28712 |
| N | 51 |
| Median | 713.79987 |

## Entorhinal Cortex

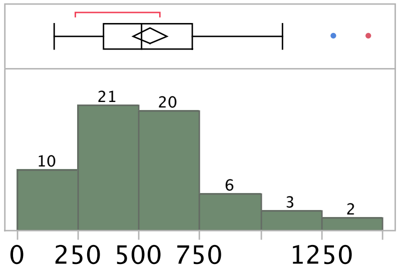


| Summary Statistics | |
|---|---|
| Mean | 544.63204 |
| Std Dev | 275.70603 |
| Std Err Mean | 35.0147 |
| Upper 99.5% Mean | 646.62038 |
| Lower 99.5% Mean | 442.64369 |
| N | 62 |
| Median | 508.82556 |

## Temporal Cortex

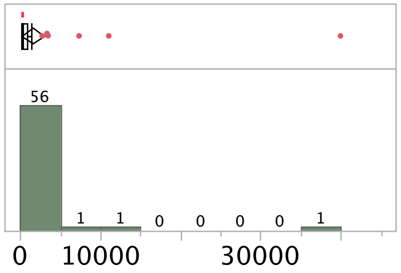


| Summary Statistics | |
|---|---|
| Mean | 1518.4119 |
| Std Dev | 5388.1495 |
| Std Err Mean | 701.47732 |
| Upper 99.5% Mean | 3565.5994 |
| Lower 99.5% Mean | -528.7757 |
| N | 59 |
| Median | 335.92794 |

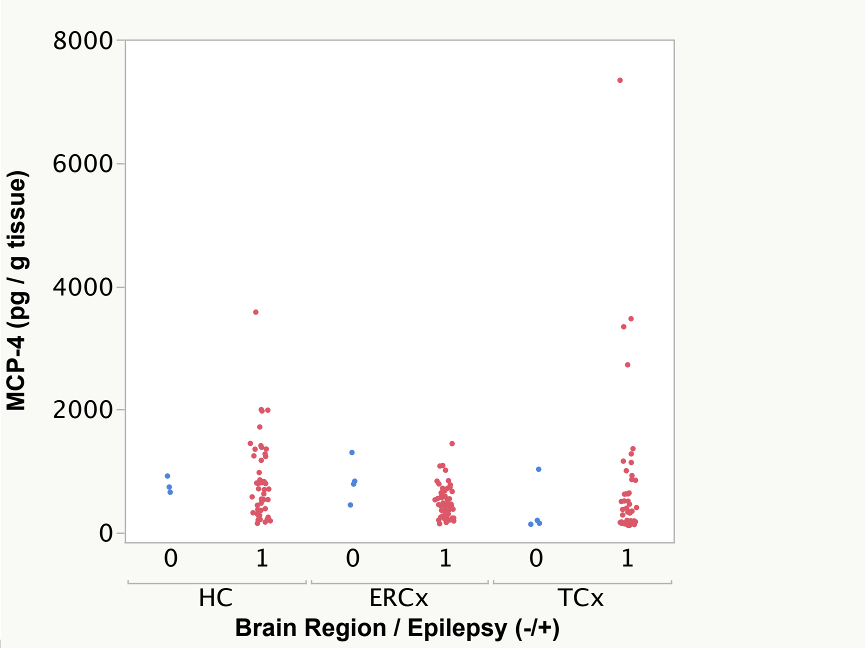


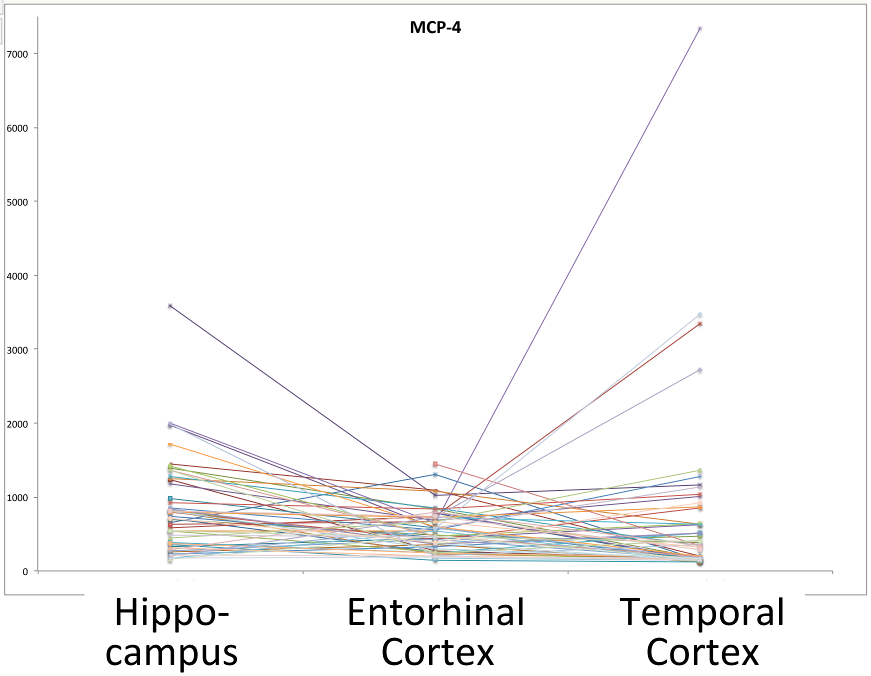


Individual cases where ERCx is lowest

| ERCx < HC | ERCx < TCx | **Total** |
|---|---|---|
| 39 | 34 | |
| 76% | 59% | **67%** |

## E. MIP-1α

### Hippocampus

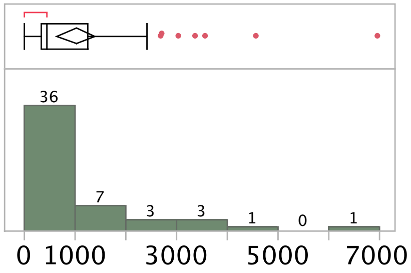


| Summary Statistics | |
|---|---|
| Mean | 1007.8384 |
| Std Dev | 1348.5148 |
| Std Err Mean | 188.82984 |
| Upper 99.5% Mean | 1562.4249 |
| Lower 99.5% Mean | 453.25194 |
| N | 51 |
| Median | 436.096 |

### Entorhinal Cortex

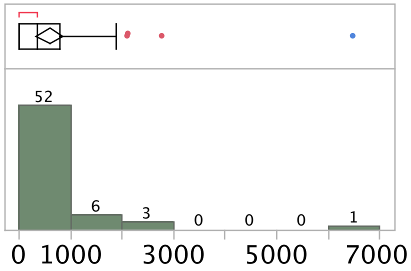


| Summary Statistics | |
|---|---|
| Mean | 589.93033 |
| Std Dev | 975.30528 |
| Std Err Mean | 123.86389 |
| Upper 99.5% Mean | 950.71233 |
| Lower 99.5% Mean | 229.14833 |
| N | 62 |
| Median | 347.99526 |

### Temporal Cortex

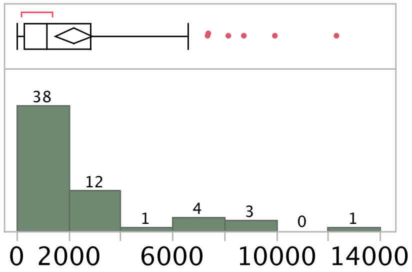


| Summary Statistics | |
|---|---|
| Mean | 2186.9905 |
| Std Dev | 2770.6965 |
| Std Err Mean | 360.71396 |
| Upper 99.5% Mean | 3239.6961 |
| Lower 99.5% Mean | 1134.2849 |
| N | 59 |
| Median | 1162.137 |

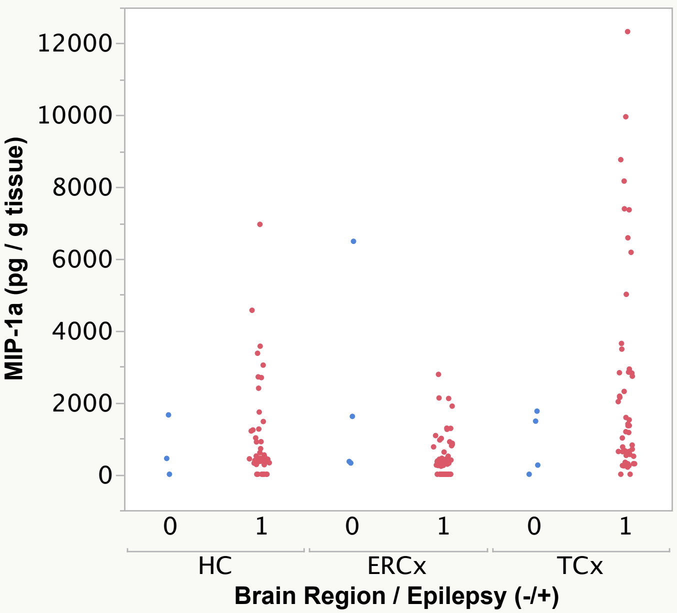


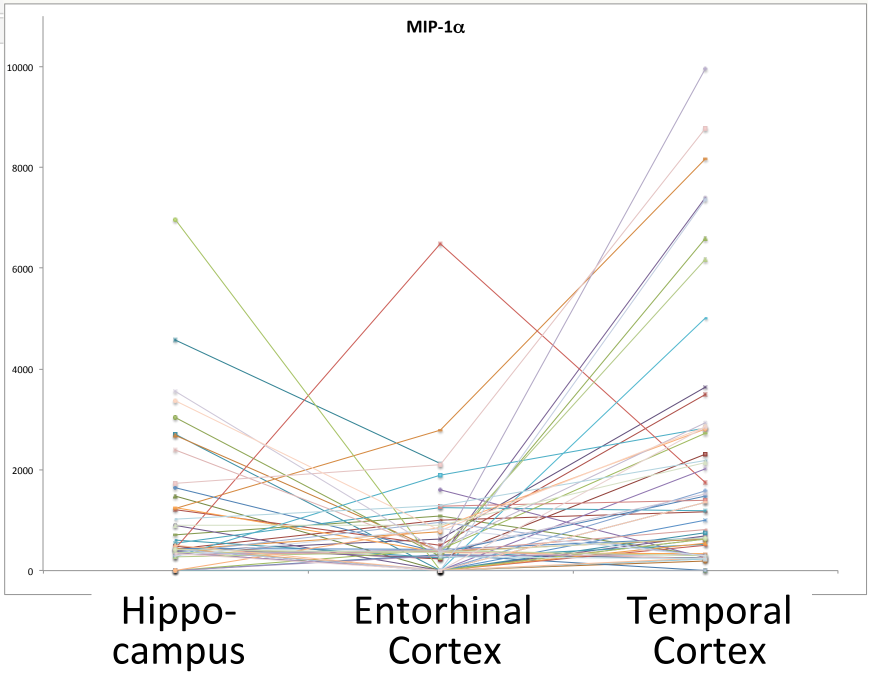

# F. MIP-1β

## Hippocampus

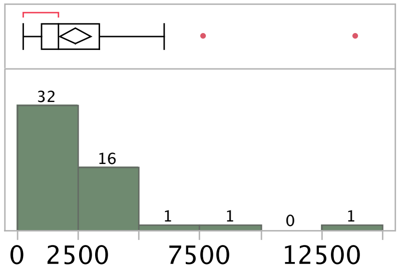


| Summary Statistics | |
|---|---|
| Mean | 2361.731 |
| Std Dev | 2217.6256 |
| Std Err Mean | 310.5297 |
| Upper 99.5% Mean | 3273.7455 |
| Lower 99.5% Mean | 1449.7164 |
| N | 51 |
| Median | 1671.8643 |

## Entorhinal Cortex

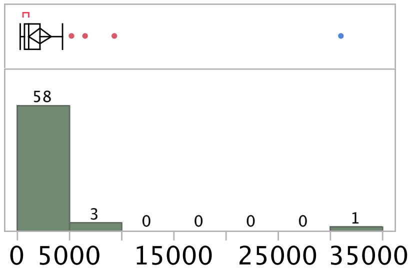


| Summary Statistics | |
|---|---|
| Mean | 2111.3537 |
| Std Dev | 4077.0412 |
| Std Err Mean | 517.78475 |
| Upper 99.5% Mean | 3619.5205 |
| Lower 99.5% Mean | 603.18685 |
| N | 62 |
| Median | 1018.9196 |

## Temporal Cortex

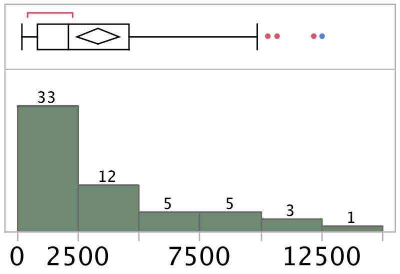


| Summary Statistics | |
|---|---|
| Mean | 3306.2185 |
| Std Dev | 3359.3066 |
| Std Err Mean | 437.34447 |
| Upper 99.5% Mean | 4582.5622 |
| Lower 99.5% Mean | 2029.8748 |
| N | 59 |
| Median | 2052.3525 |

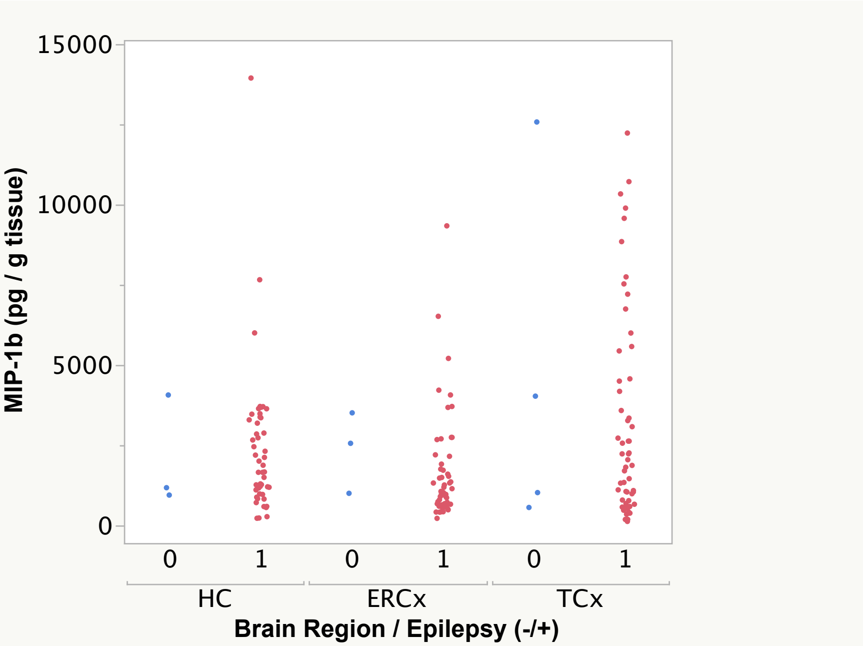


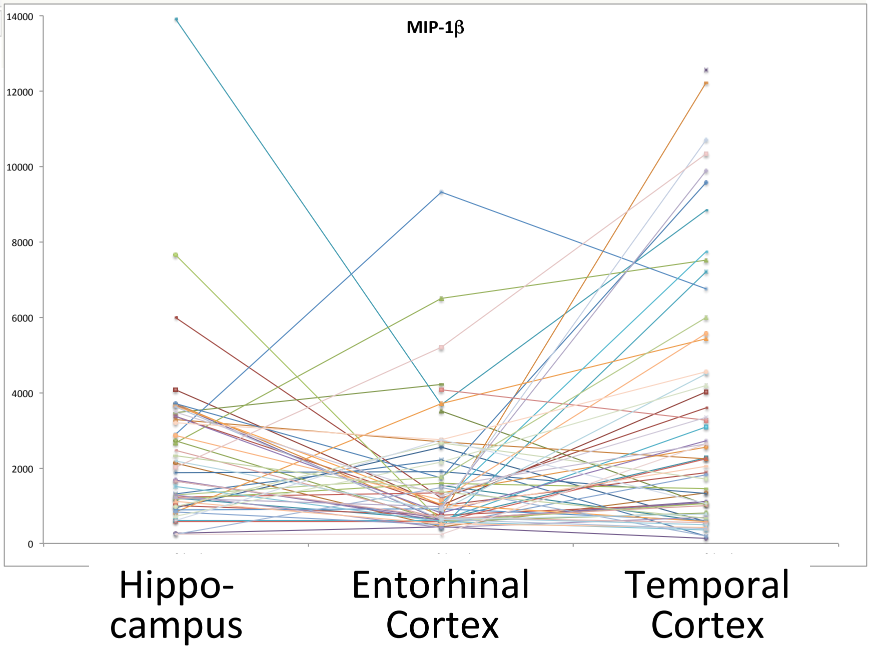

# G. TARC

## Hippocampus

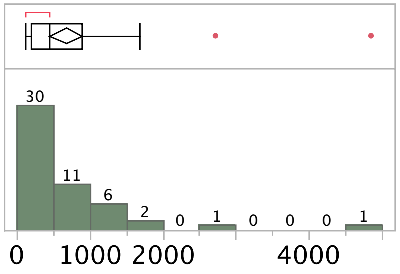


| Summary Statistics | |
|---|---|
| Mean | 665.85435 |
| Std Dev | 795.59463 |
| Std Err Mean | 111.40553 |
| Upper 99.5% Mean | 993.0484 |
| Lower 99.5% Mean | 338.66031 |
| N | 51 |
| Median | 426.77107 |

## Entorhinal Cortex

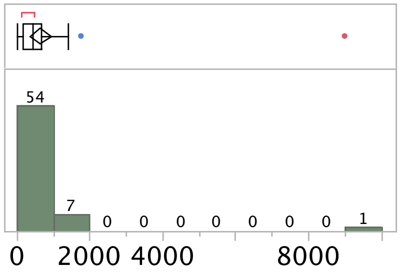


| Summary Statistics | |
|---|---|
| Mean | 637.9818 |
| Std Dev | 1143.4874 |
| Std Err Mean | 145.22305 |
| Upper 99.5% Mean | 1060.9772 |
| Lower 99.5% Mean | 214.98637 |
| N | 62 |
| Median | 430.47761 |

## Temporal Cortex

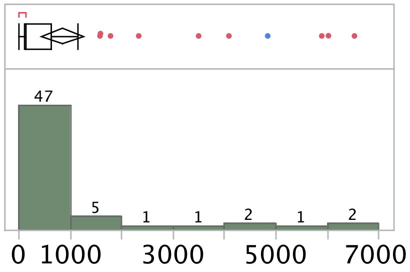


| Summary Statistics | |
|---|---|
| Mean | 854.89899 |
| Std Dev | 1576.1304 |
| Std Err Mean | 205.1947 |
| Upper 99.5% Mean | 1453.7381 |
| Lower 99.5% Mean | 256.05992 |
| N | 59 |
| Median | 128.32976 |

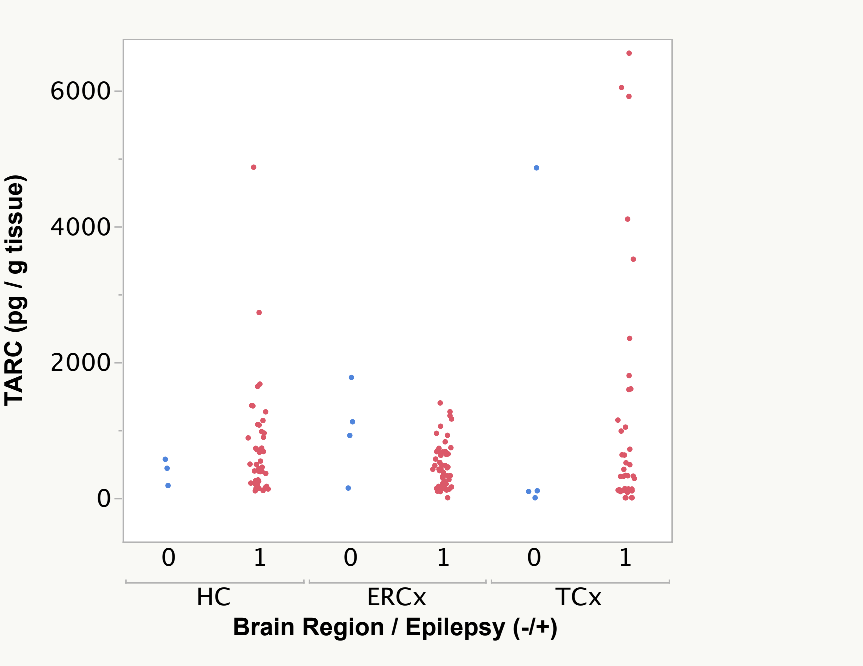


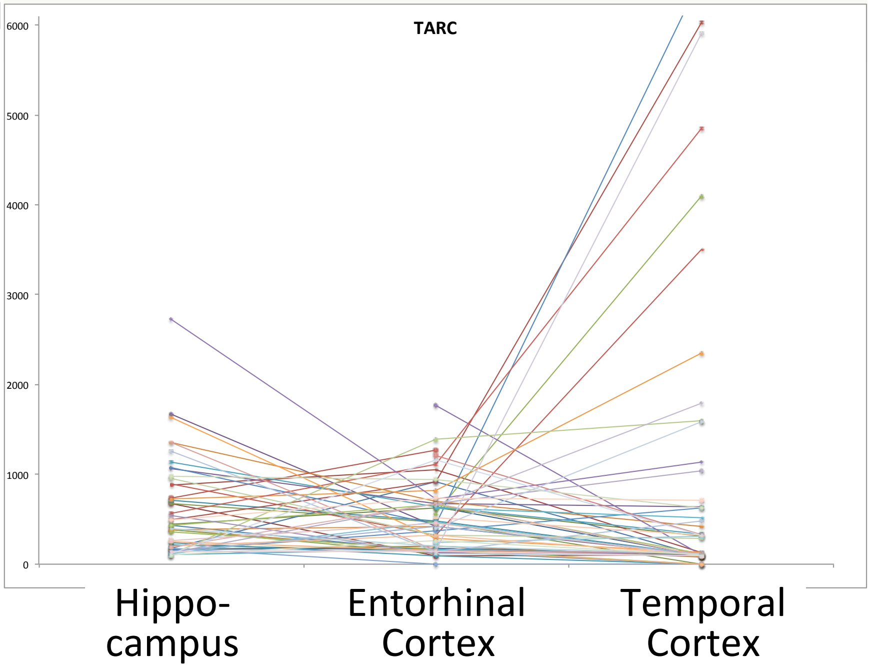

# H. GM-CSF

## Hippocampus

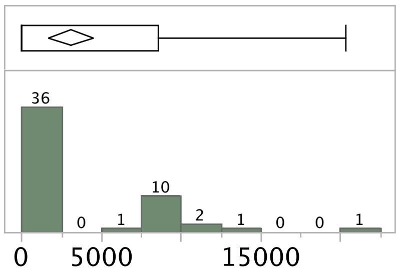


| Summary Statistics | |
|---|---|
| Mean | 3062.07 |
| Std Dev | 5068.9838 |
| Std Err Mean | 709.79971 |
| Upper 99.5% Mean | 5146.7262 |
| Lower 99.5% Mean | 977.41372 |
| N | 51 |
| Median | 0 |

## Entorhinal Cortex

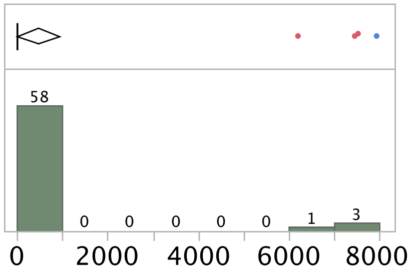


| Summary Statistics | |
|---|---|
| Mean | 469.58711 |
| Std Dev | 1810.3773 |
| Std Err Mean | 229.91815 |
| Upper 99.5% Mean | 1139.2764 |
| Lower 99.5% Mean | −200.1022 |
| N | 62 |
| Median | 0 |

## Temporal Cortex

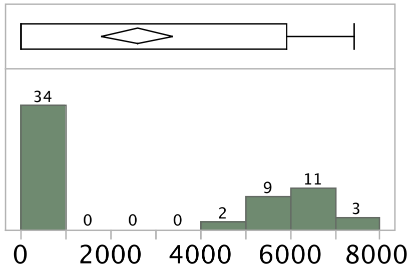


| Summary Statistics | |
|---|---|
| Mean | 2578.7104 |
| Std Dev | 3075.8234 |
| Std Err Mean | 400.4381 |
| Upper 99.5% Mean | 3747.3467 |
| Lower 99.5% Mean | 1410.074 |
| N | 59 |
| Median | 0 |

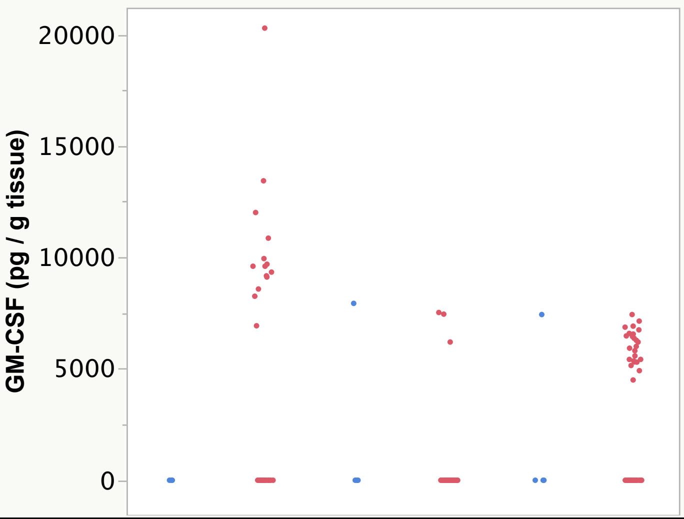


| GM-CSF LEVELS | HIPPOCAMPUS | | ENTORHINAL CORTEX | | TEMPORAL CORTEX | |
|---|---|---|---|---|---|---|
| Non-/Epileptic+ | - | + | - | + | - | + |
| n | 3 | 48 | 4 | 58 | 4 | 55 |
| (pg / g tissue) | **0** | **3253 ± 746** | **1984 ± 1984** | **366 ± 208** | **1859 ± 1859** | **2631 ± 412** |

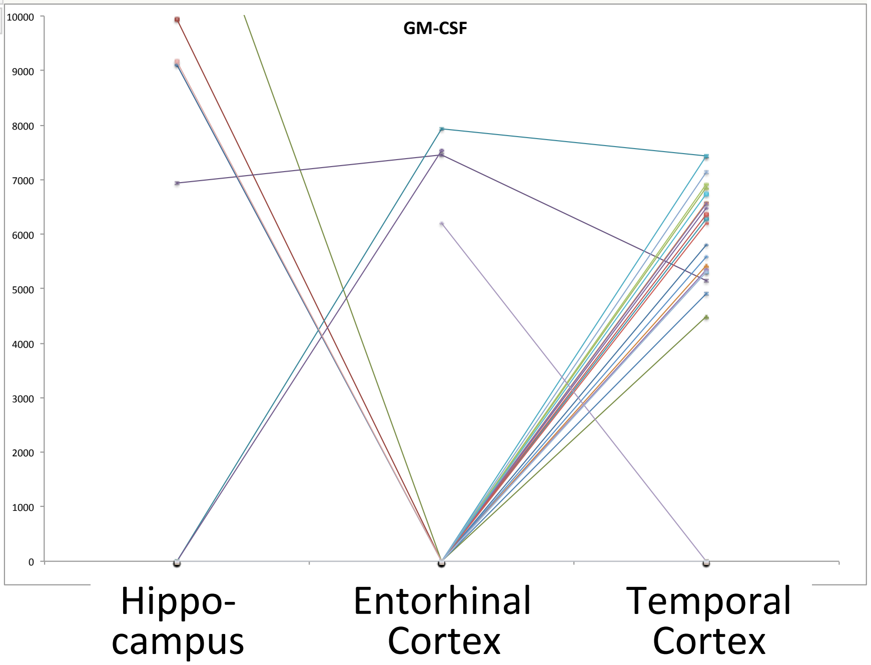

# I. IL-1α

## Hippocampus

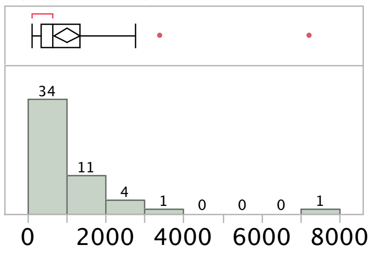


| Summary Statistics | |
|---|---|
| Mean | 991.79428 |
| Std Dev | 1157.6925 |
| Std Err Mean | 162.10937 |
| Upper 99.5% Mean | 1467.9037 |
| Lower 99.5% Mean | 515.68487 |
| N | 51 |
| Median | 607.24832 |

## Entorhinal Cortex

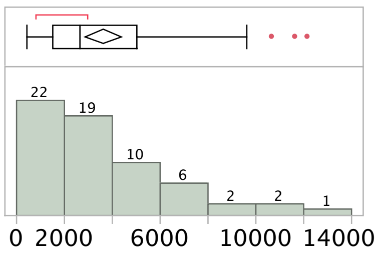


| Summary Statistics | |
|---|---|
| Mean | 3616.1414 |
| Std Dev | 2926.1712 |
| Std Err Mean | 371.62412 |
| Upper 99.5% Mean | 4698.5819 |
| Lower 99.5% Mean | 2533.701 |
| N | 62 |
| Median | 2639.8208 |

## Temporal Cortex

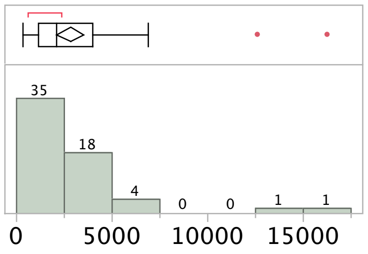


| Summary Statistics | |
|---|---|
| Mean | 2808.4881 |
| Std Dev | 2714.781 |
| Std Err Mean | 353.43439 |
| Upper 99.5% Mean | 3839.9491 |
| Lower 99.5% Mean | 1777.0271 |
| N | 59 |
| Median | 2089.163 |

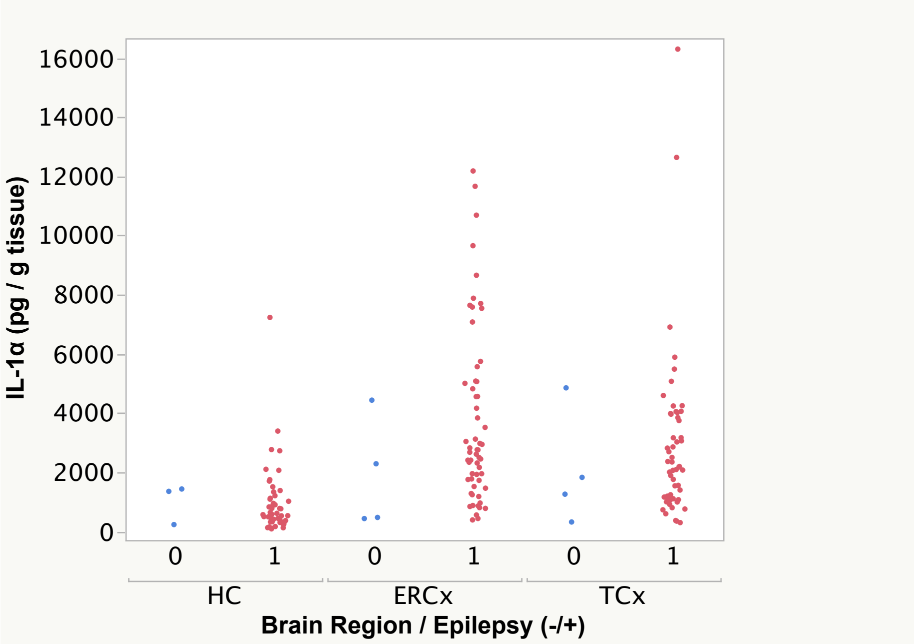


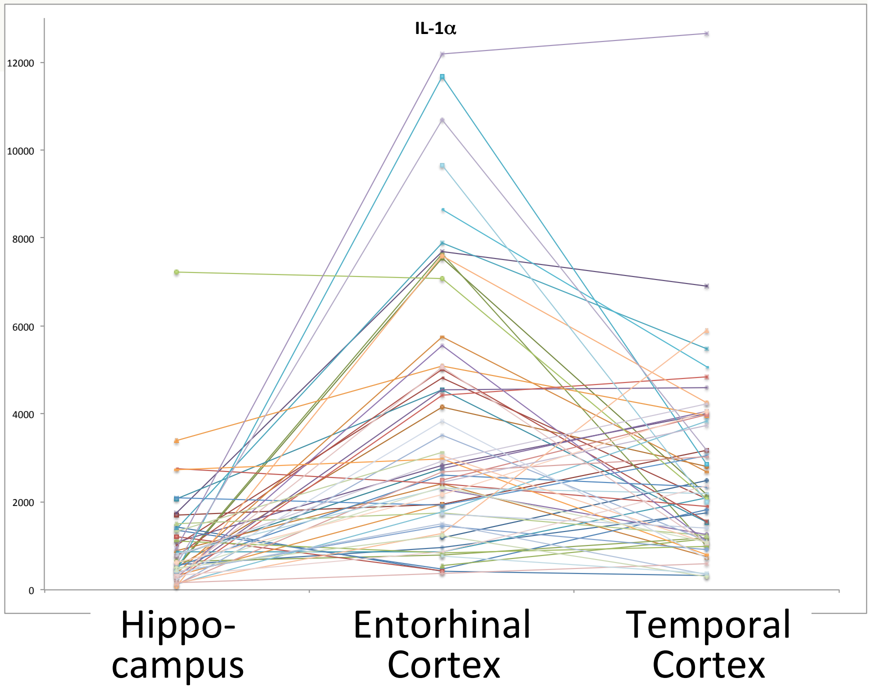

J. IL-1β

Hippocampus

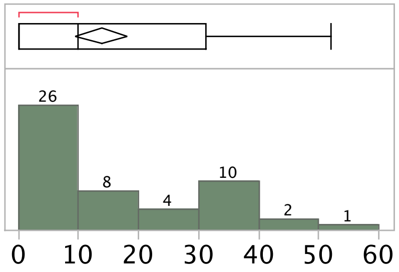


| Summary Statistics | |
|---|---|
| Mean | 13.832381 |
| Std Dev | 15.433618 |
| Std Err Mean | 2.1611388 |
| Upper 99.5% Mean | 20.179568 |
| Lower 99.5% Mean | 7.485194 |
| N | 51 |
| Median | 9.8099062 |

Entorhinal Cortex

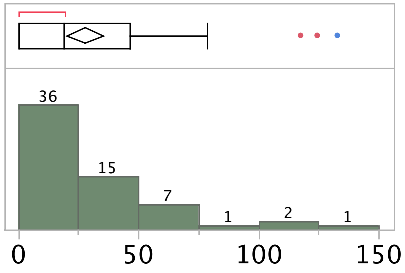


| Summary Statistics | |
|---|---|
| Mean | 27.81259 |
| Std Dev | 30.56279 |
| Std Err Mean | 3.8814782 |
| Upper 99.5% Mean | 39.118285 |
| Lower 99.5% Mean | 16.506894 |
| N | 62 |
| Median | 18.858961 |

Temporal Cortex

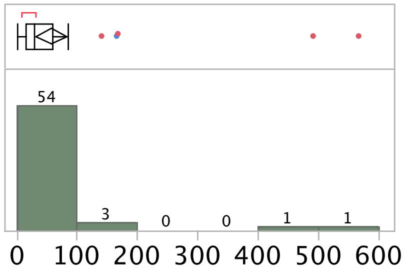


| Summary Statistics | |
|---|---|
| Mean | 56.275181 |
| Std Dev | 97.146367 |
| Std Err Mean | 12.64738 |
| Upper 99.5% Mean | 93.185224 |
| Lower 99.5% Mean | 19.365139 |
| N | 59 |
| Median | 28.803695 |

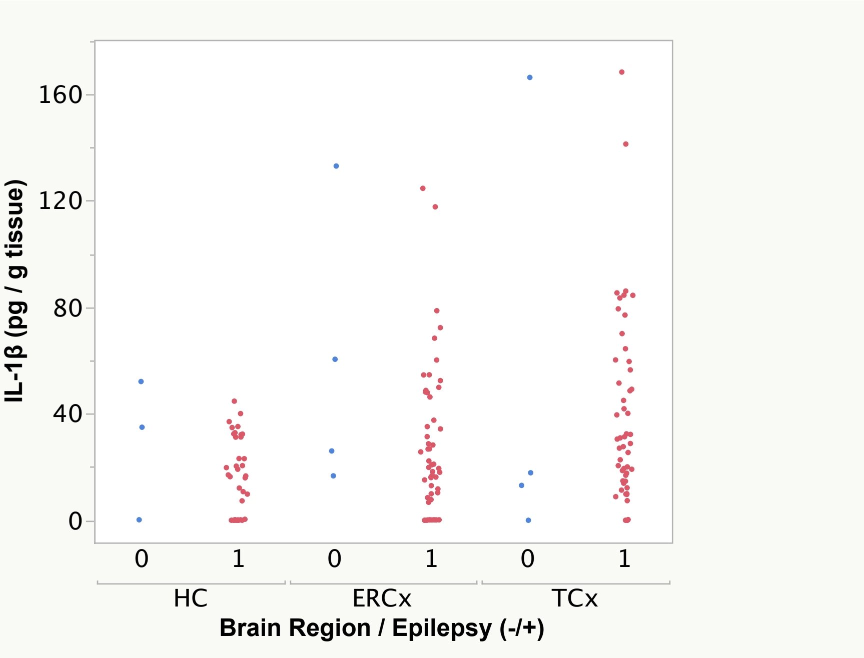


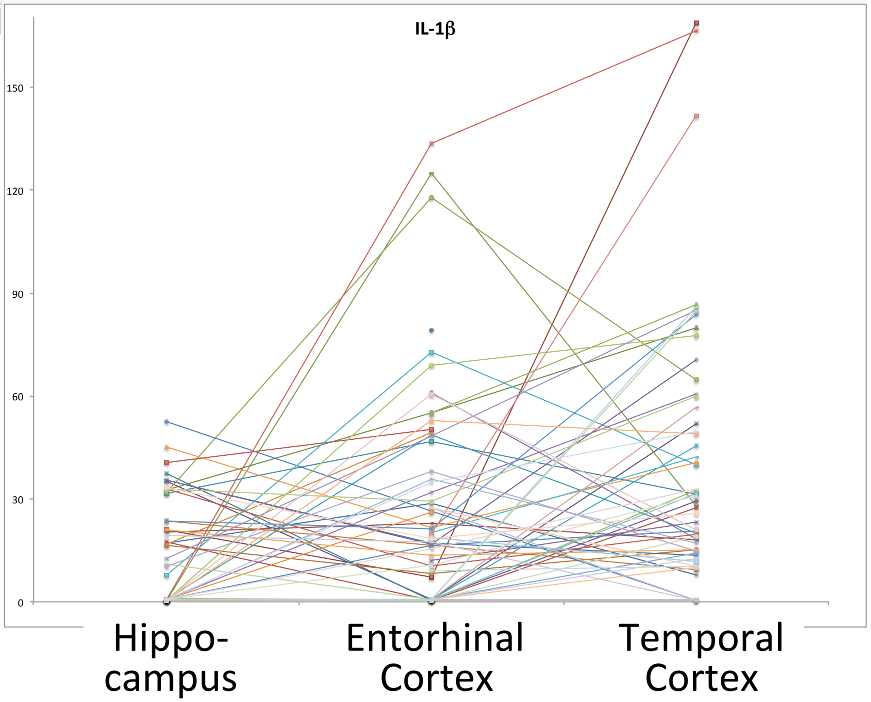

# K. IL-2

## Hippocampus

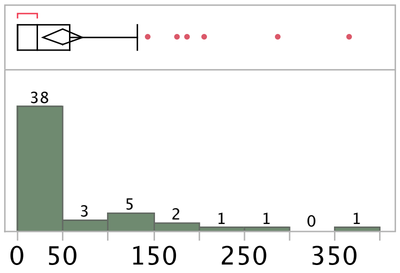


| Summary Statistics | |
|---|---|
| Mean | 49.624907 |
| Std Dev | 78.613536 |
| Std Err Mean | 11.008097 |
| Upper 99.5% Mean | 81.955293 |
| Lower 99.5% Mean | 17.294522 |
| N | 51 |
| Median | 22.276405 |

## Entorhinal Cortex

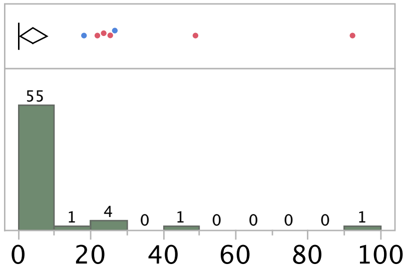


| Summary Statistics | |
|---|---|
| Mean | 4.1611047 |
| Std Dev | 14.39908 |
| Std Err Mean | 1.828685 |
| Upper 99.5% Mean | 9.487569 |
| Lower 99.5% Mean | −1.16536 |
| N | 62 |
| Median | 0 |

## Temporal Cortex

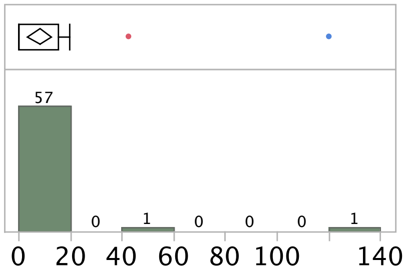


| Summary Statistics | |
|---|---|
| Mean | 7.9718566 |
| Std Dev | 17.409582 |
| Std Err Mean | 2.2665345 |
| Upper 99.5% Mean | 14.586498 |
| Lower 99.5% Mean | 1.3572149 |
| N | 59 |
| Median | 0 |

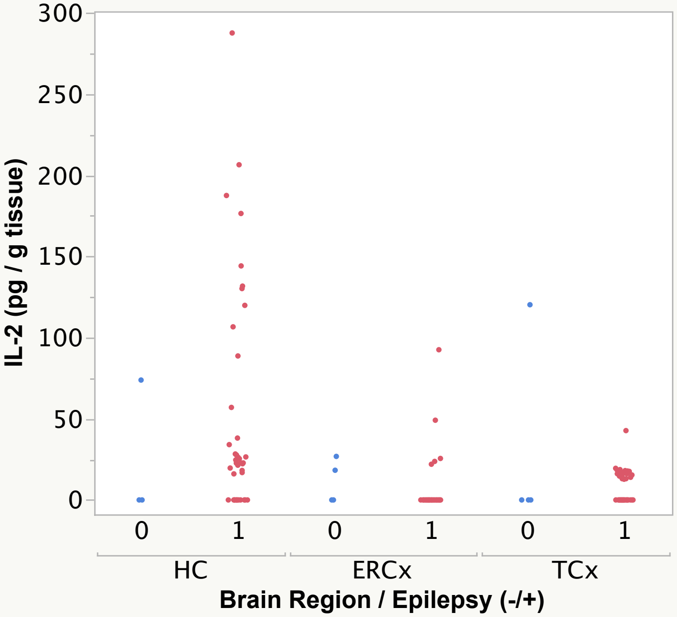


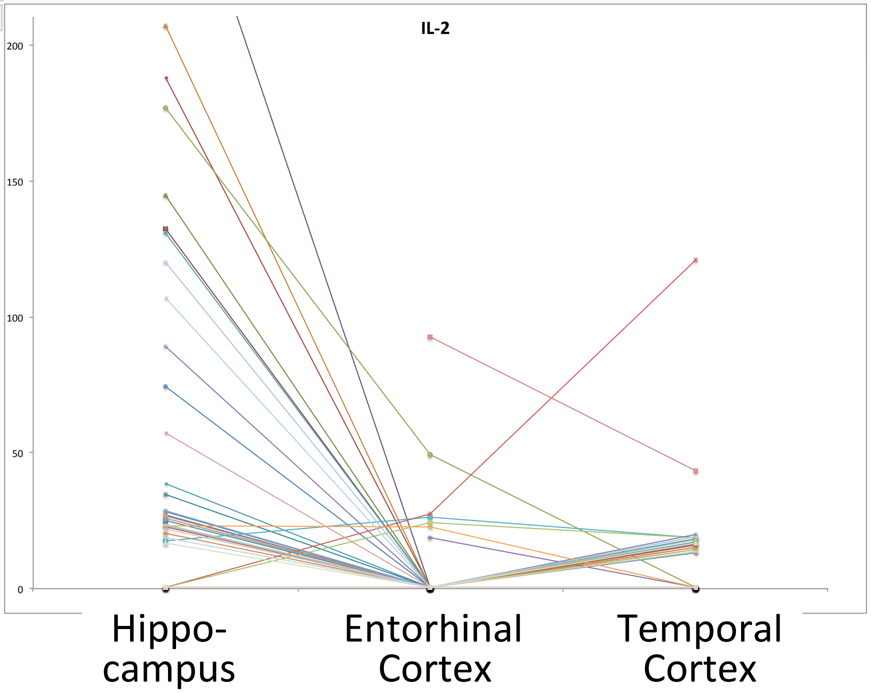

# L. IL-4

## Hippocampus

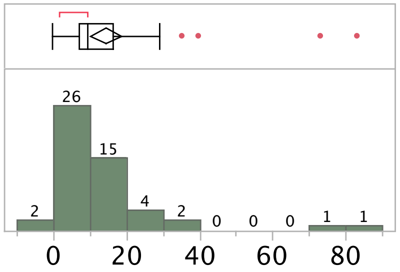


| Summary Statistics | |
|---|---|
| Mean | 14.261259 |
| Std Dev | 15.47387 |
| Std Err Mean | 2.1667751 |
| Upper 99.5% Mean | 20.625 |
| Lower 99.5% Mean | 7.897518 |
| N | 51 |
| Median | 8.9967163 |

## Entorhinal Cortex

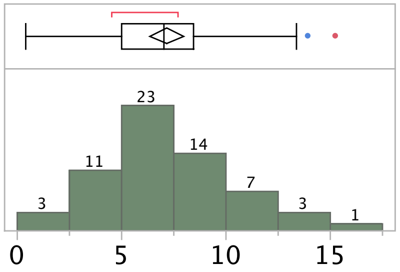


| Summary Statistics | |
|---|---|
| Mean | 7.1697472 |
| Std Dev | 3.1402124 |
| Std Err Mean | 0.3988074 |
| Upper 99.5% Mean | 8.3313652 |
| Lower 99.5% Mean | 6.0081293 |
| N | 62 |
| Median | 7.0182008 |

## Temporal Cortex

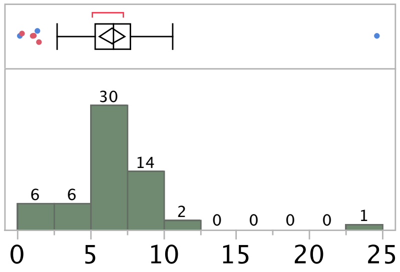


| Summary Statistics | |
|---|---|
| Mean | 6.4481769 |
| Std Dev | 3.3879187 |
| Std Err Mean | 0.4410694 |
| Upper 99.5% Mean | 7.7353915 |
| Lower 99.5% Mean | 5.1609622 |
| N | 59 |
| Median | 6.5068842 |

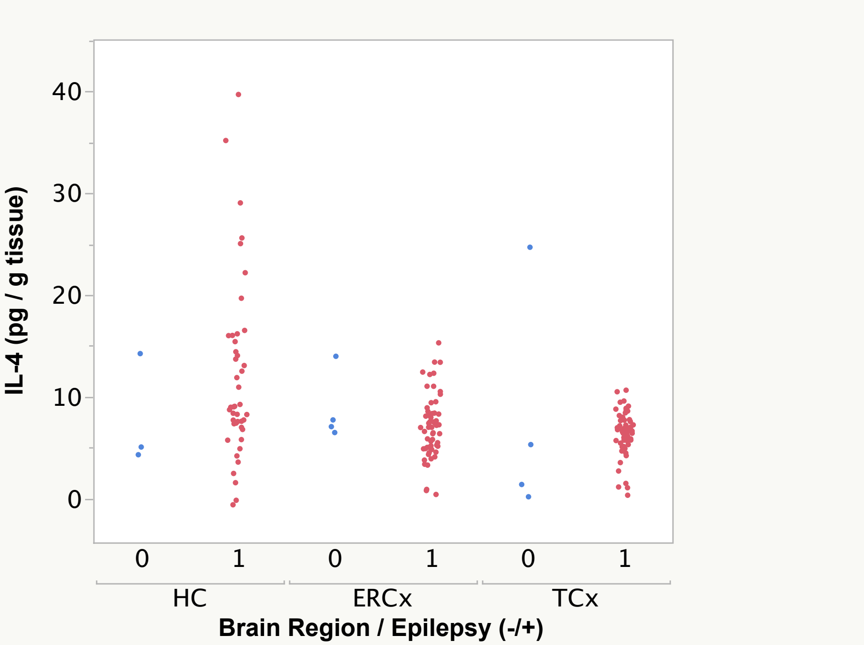


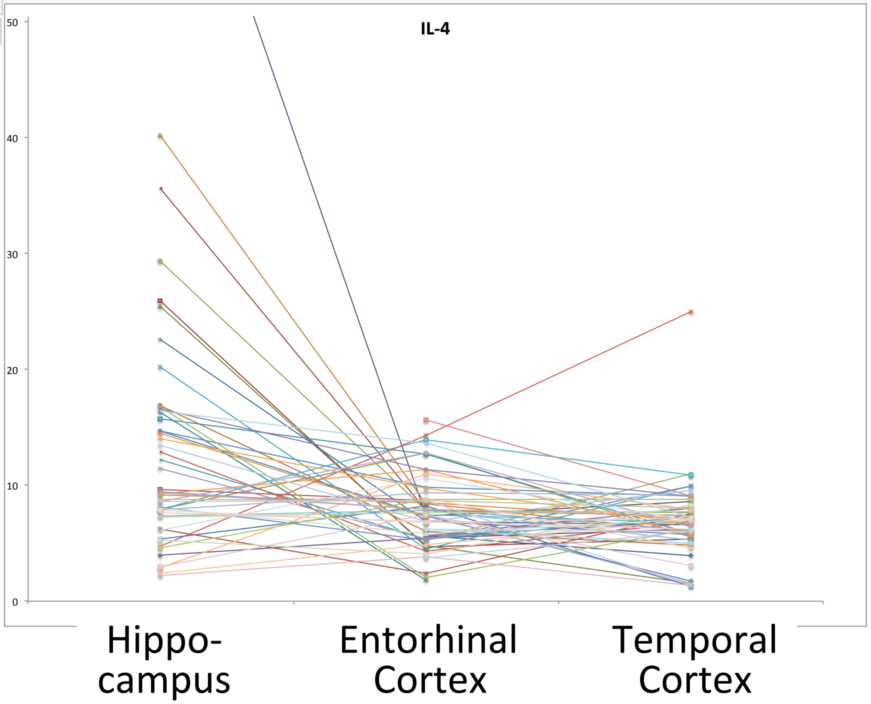

# M. IL-6

## Hippocampus

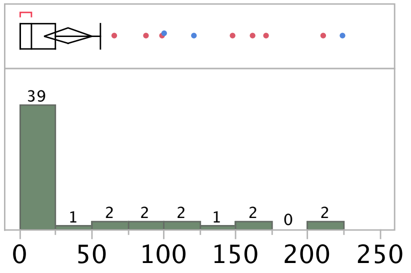


| Summary Statistics | |
|---|---|
| Mean | 33.358479 |
| Std Dev | 58.234582 |
| Std Err Mean | 8.1544726 |
| Upper 99.5% Mean | 57.307872 |
| Lower 99.5% Mean | 9.4090857 |
| N | 51 |
| Median | 8.4504144 |

## Entorhinal Cortex

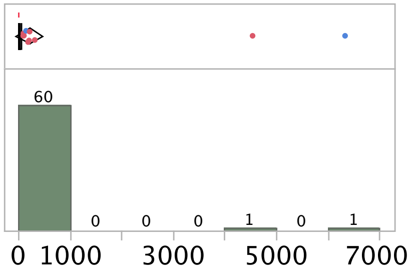


| Summary Statistics | |
|---|---|
| Mean | 209.28161 |
| Std Dev | 979.0813 |
| Std Err Mean | 124.34345 |
| Upper 99.5% Mean | 571.46043 |
| Lower 99.5% Mean | −152.8972 |
| N | 62 |
| Median | 8.3029382 |

## Temporal Cortex

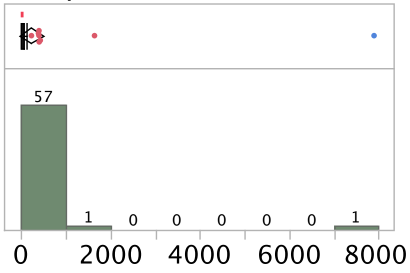


| Summary Statistics | |
|---|---|
| Mean | 220.35951 |
| Std Dev | 1043.3167 |
| Std Err Mean | 135.82827 |
| Upper 99.5% Mean | 616.75997 |
| Lower 99.5% Mean | −176.041 |
| N | 59 |
| Median | 15.527197 |

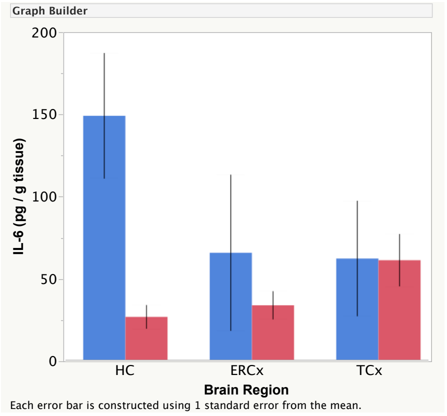


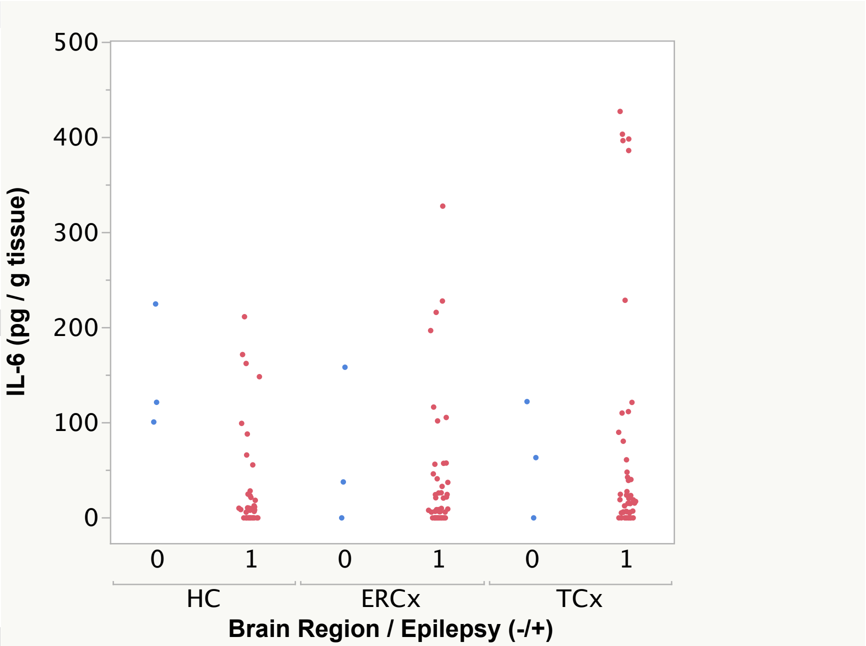


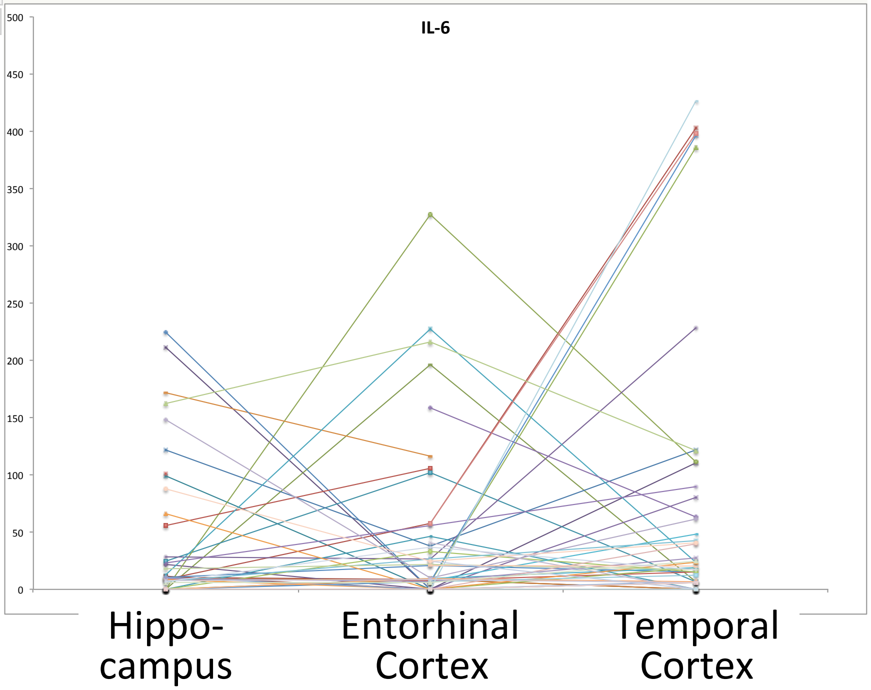

# N. IL-8

## Hippocampus

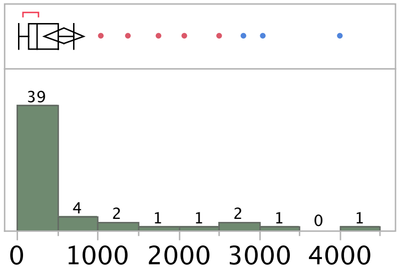


| Summary Statistics | |
|---|---|
| Mean | 569.66385 |
| Std Dev | 860.36765 |
| Std Err Mean | 120.47557 |
| Upper 99.5% Mean | 923.49627 |
| Lower 99.5% Mean | 215.83143 |
| N | 51 |
| Median | 242.83711 |

## Entorhinal Cortex

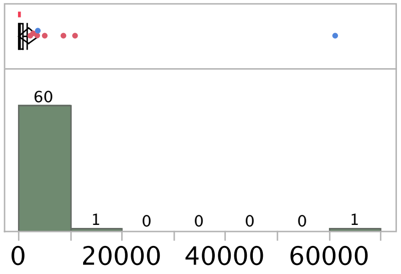


| Summary Statistics | |
|---|---|
| Mean | 1905.9706 |
| Std Dev | 7907.0708 |
| Std Err Mean | 1004.199 |
| Upper 99.5% Mean | 4830.9305 |
| Lower 99.5% Mean | −1018.989 |
| N | 62 |
| Median | 241.63866 |

## Temporal Cortex

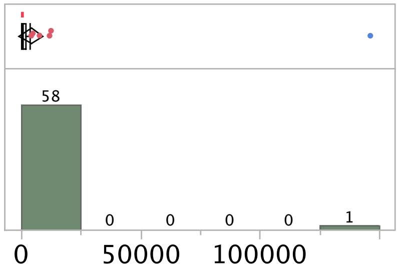


| Summary Statistics | |
|---|---|
| Mean | 3928.4278 |
| Std Dev | 19024.88 |
| Std Err Mean | 2476.8283 |
| Upper 99.5% Mean | 11156.79 |
| Lower 99.5% Mean | −3299.934 |
| N | 59 |
| Median | 484.88101 |

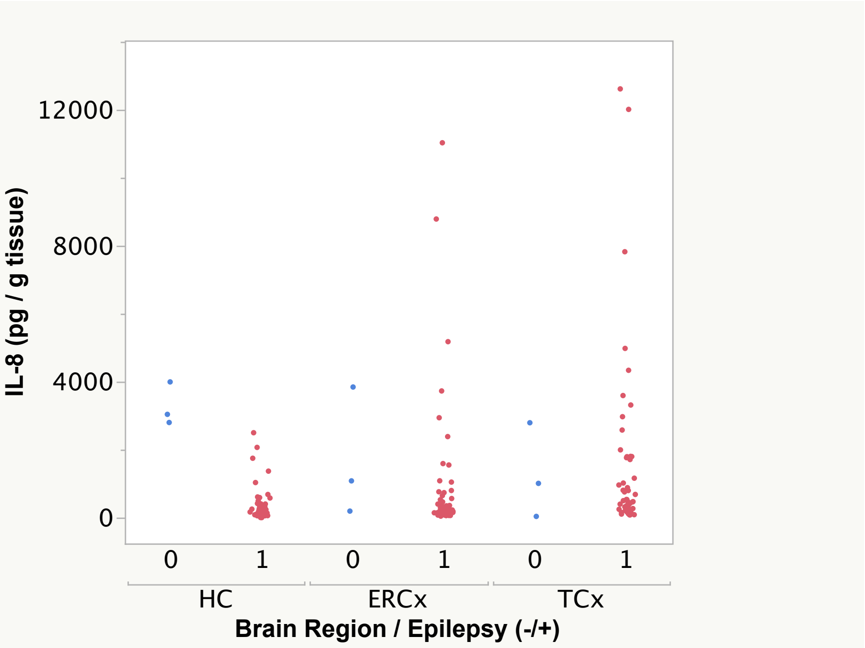


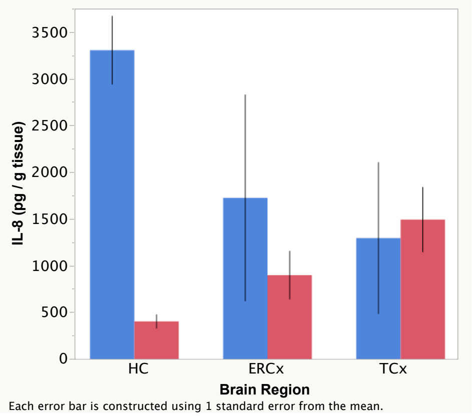


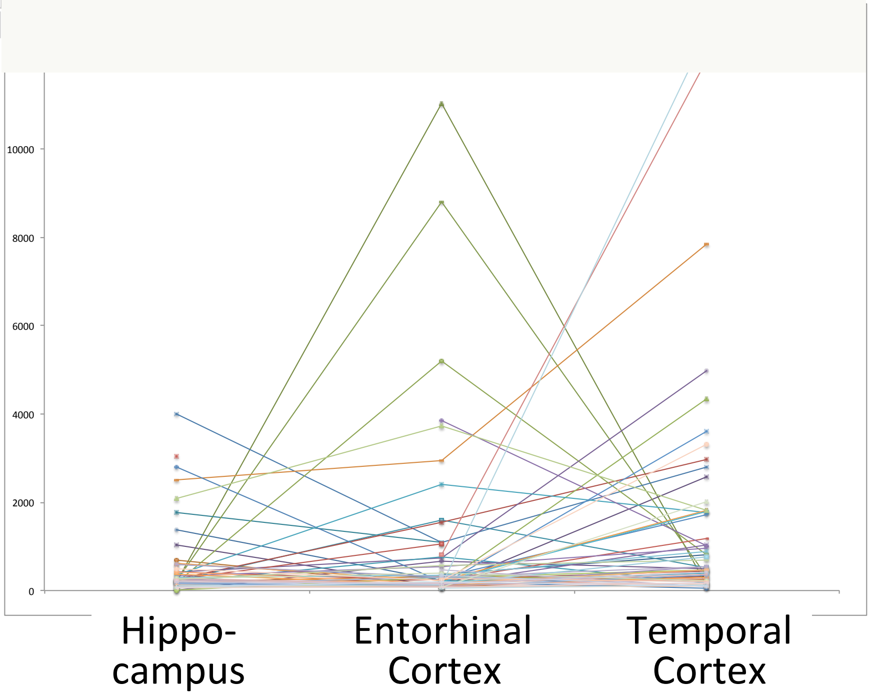

# O. IL-10

## Hippocampus

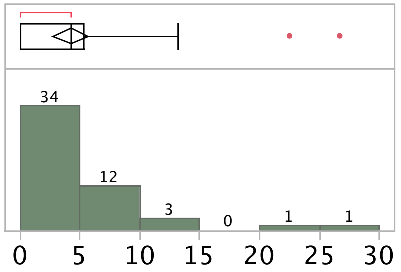


| Summary Statistics | |
|---|---|
| Mean | 4.211043 |
| Std Dev | 5.3194884 |
| Std Err Mean | 0.7448774 |
| Upper 99.5% Mean | 6.3987211 |
| Lower 99.5% Mean | 2.0233649 |
| N | 51 |
| Median | 4.2476791 |

## Entorhinal Cortex

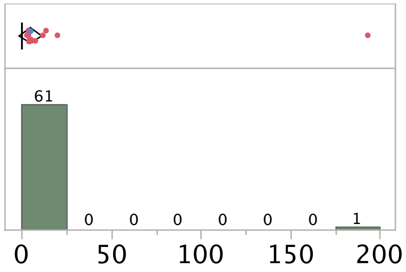


| Summary Statistics | |
|---|---|
| Mean | 4.5855709 |
| Std Dev | 24.620203 |
| Std Err Mean | 3.1267689 |
| Upper 99.5% Mean | 13.693002 |
| Lower 99.5% Mean | −4.521861 |
| N | 62 |
| Median | 0 |

## Temporal Cortex

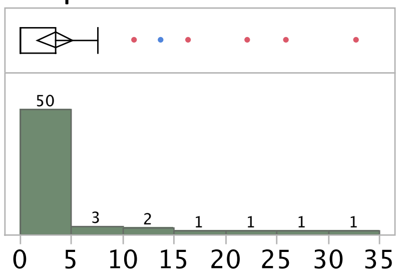


| Summary Statistics | |
|---|---|
| Mean | 3.3735657 |
| Std Dev | 6.5513844 |
| Std Err Mean | 0.8529176 |
| Upper 99.5% Mean | 5.8627157 |
| Lower 99.5% Mean | 0.8844157 |
| N | 59 |
| Median | 0 |

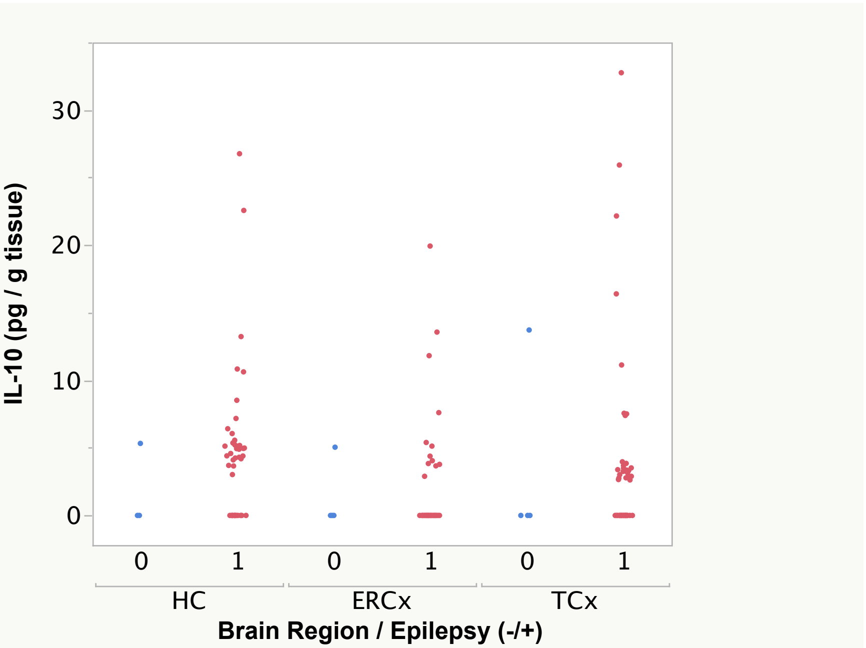


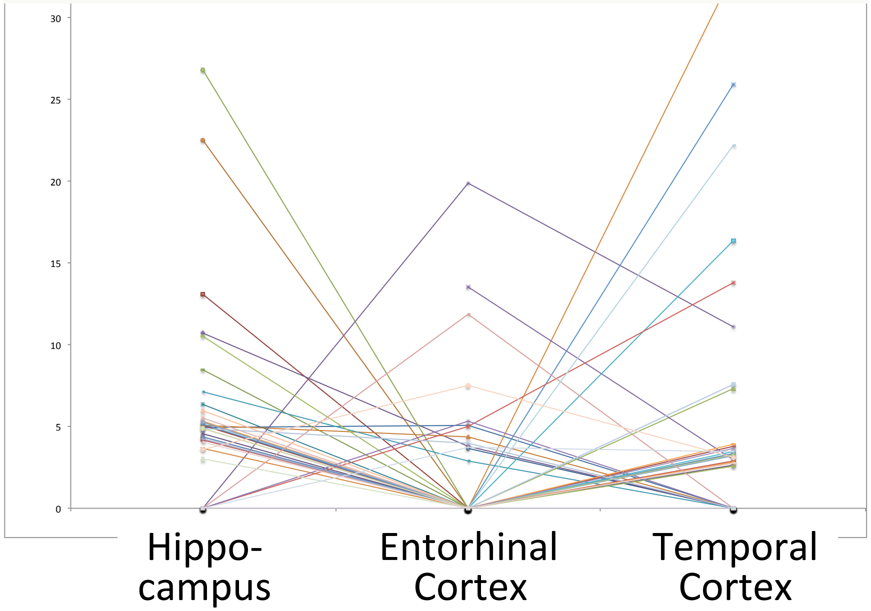

## P. IL-12/23 p40 [see supplemental data 2AA for IL-12/23p40:IL-12p70 ratio]

### Hippocampus

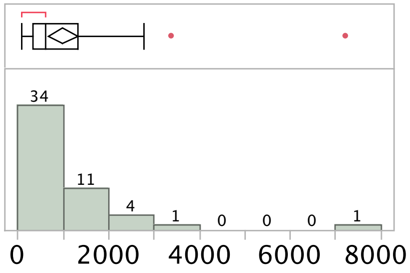


| Summary Statistics | |
|---|---|
| Mean | 991.60372 |
| Std Dev | 1157.6942 |
| Std Err Mean | 162.10961 |
| Upper 99.5% Mean | 1467.7138 |
| Lower 99.5% Mean | 515.49362 |
| N | 51 |
| Median | 607.24832 |

### Entorhinal Cortex

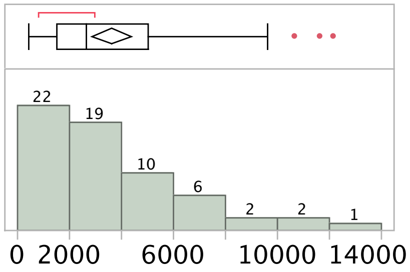


| Summary Statistics | |
|---|---|
| Mean | 3616.1477 |
| Std Dev | 2926.1655 |
| Std Err Mean | 371.62339 |
| Upper 99.5% Mean | 4698.5861 |
| Lower 99.5% Mean | 2533.7094 |
| N | 62 |
| Median | 2639.96 |

### Temporal Cortex

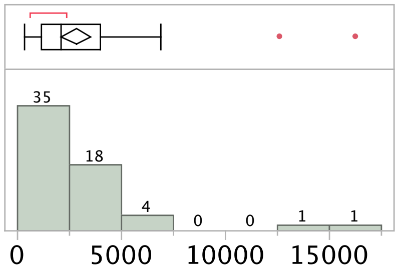


| Summary Statistics | |
|---|---|
| Mean | 2808.4707 |
| Std Dev | 2714.7821 |
| Std Err Mean | 353.43453 |
| Upper 99.5% Mean | 3839.932 |
| Lower 99.5% Mean | 1777.0093 |
| N | 59 |
| Median | 2089.0416 |

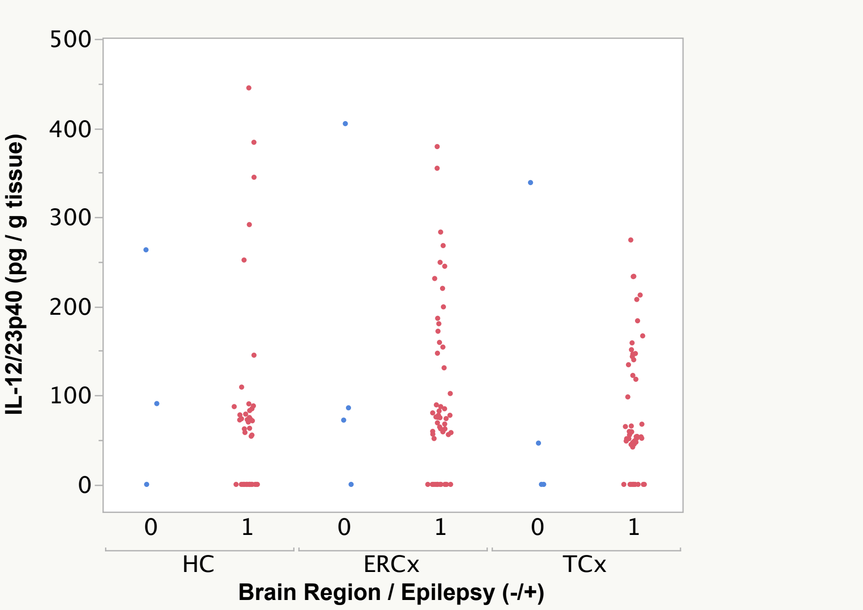


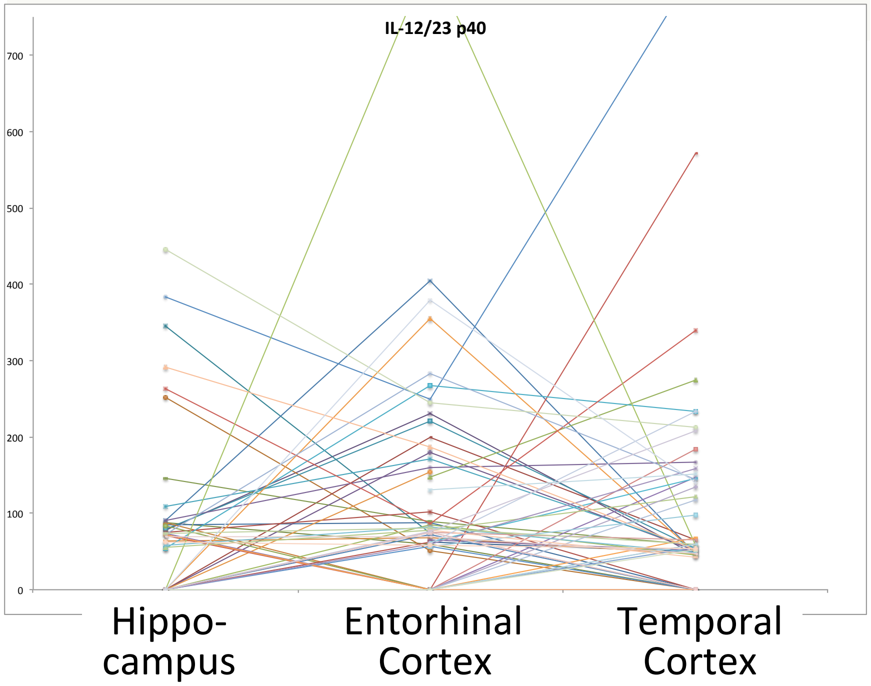

# Q. IL-12 p70

[see supplemental data 2Z for IL-12p70:IL-12/23p40 ratio]

## Hippocampus

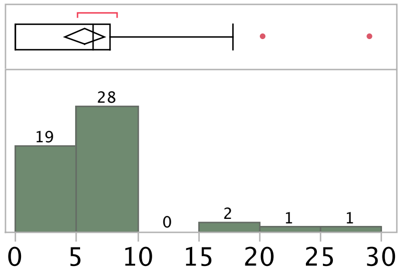


| Summary Statistics | |
|---|---|
| Mean | 5.6366689 |
| Std Dev | 5.8279984 |
| Std Err Mean | 0.816083 |
| Upper 99.5% Mean | 8.0334753 |
| Lower 99.5% Mean | 3.2398624 |
| N | 51 |
| Median | 6.3309553 |

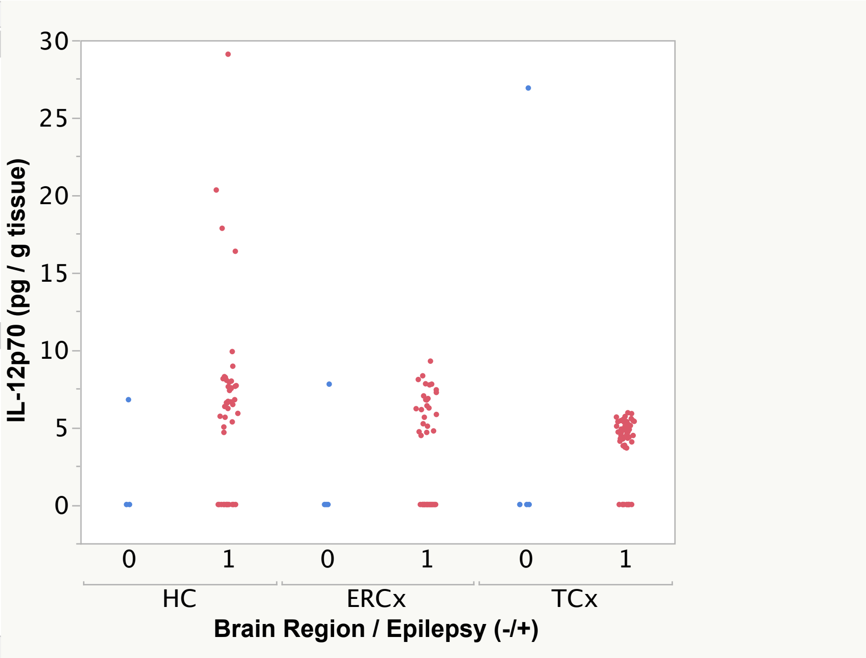


## Entorhinal Cortex

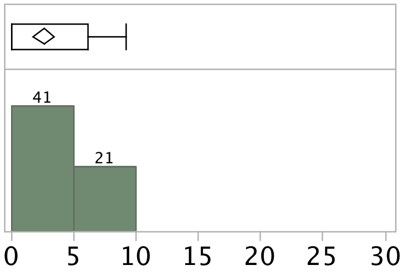


| Summary Statistics | |
|---|---|
| Mean | 2.6456181 |
| Std Dev | 3.3462496 |
| Std Err Mean | 0.4249741 |
| Upper 99.5% Mean | 3.8834527 |
| Lower 99.5% Mean | 1.4077835 |
| N | 62 |
| Median | 0 |

## Temporal Cortex

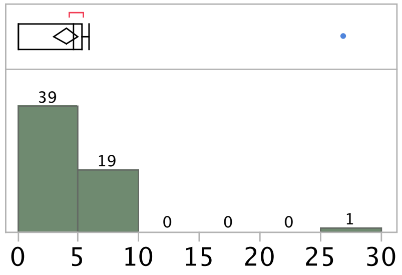


| Summary Statistics | |
|---|---|
| Mean | 3.9873365 |
| Std Dev | 3.739264 |
| Std Err Mean | 0.4868107 |
| Upper 99.5% Mean | 5.4080422 |
| Lower 99.5% Mean | 2.5666309 |
| N | 59 |
| Median | 4.5896543 |

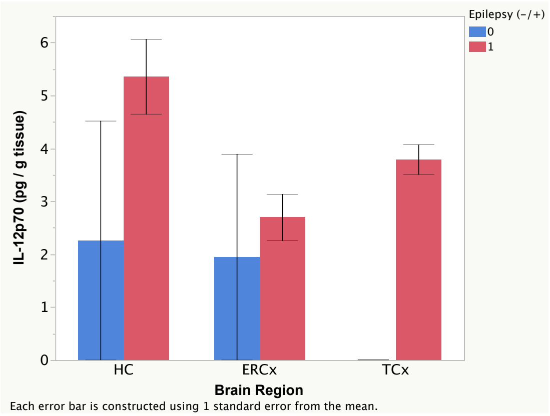


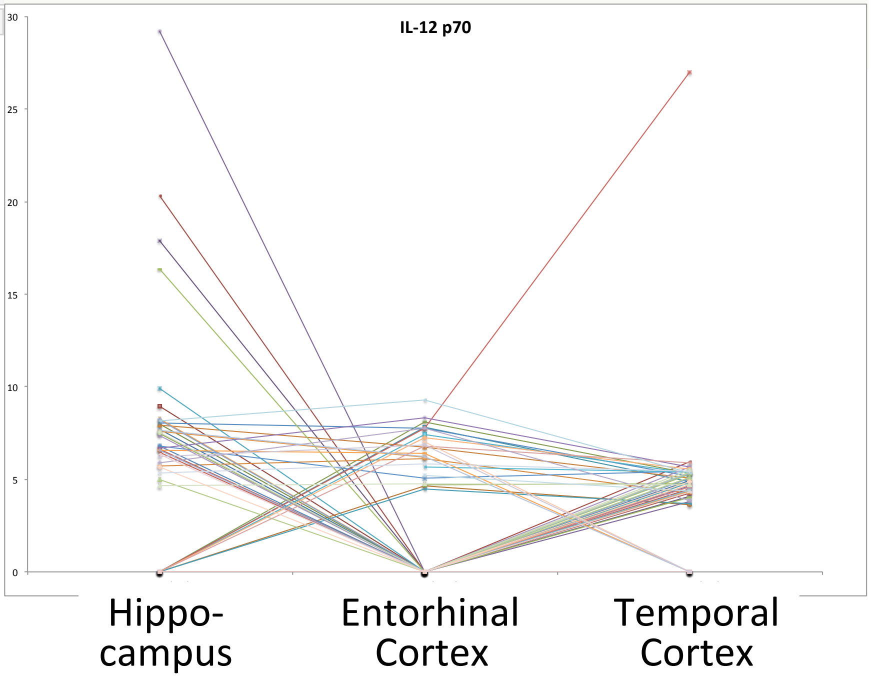

# R. IL-17A

## Hippocampus

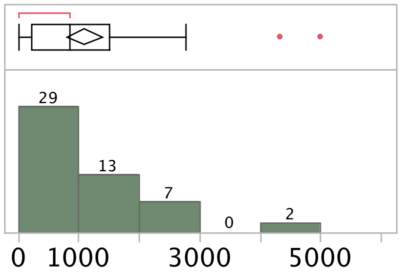


| Summary Statistics | |
|---|---|
| Mean | 1094.9051 |
| Std Dev | 1059.5481 |
| Std Err Mean | 148.36641 |
| Upper 99.5% Mean | 1530.6519 |
| Lower 99.5% Mean | 659.15826 |
| N | 51 |
| Median | 855.98746 |

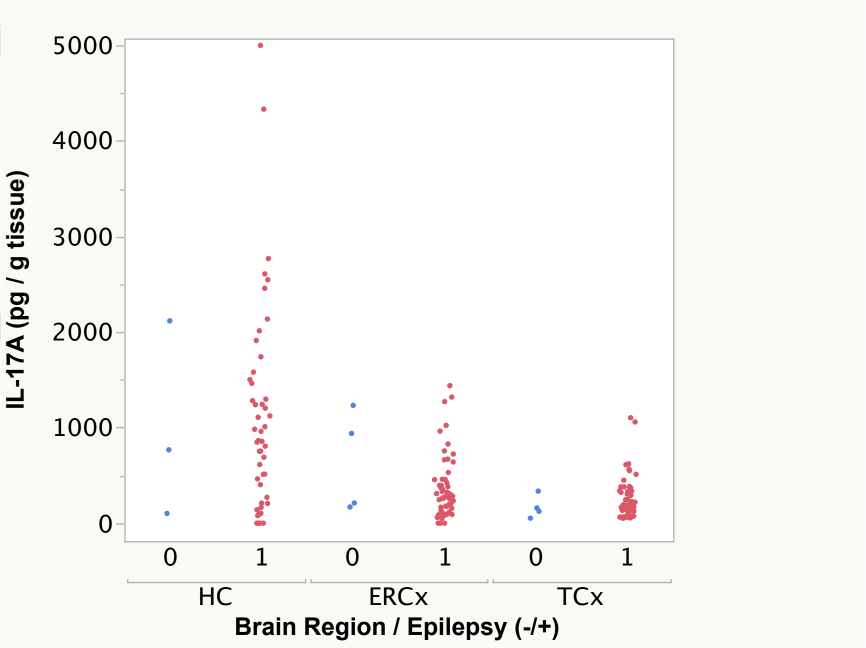


## Entorhinal Cortex

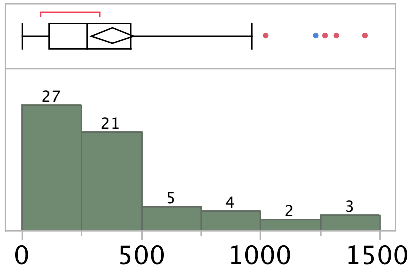


| Summary Statistics | |
|---|---|
| Mean | 378.2263 |
| Std Dev | 348.22324 |
| Std Err Mean | 44.224395 |
| Upper 99.5% Mean | 507.03999 |
| Lower 99.5% Mean | 249.41261 |
| N | 62 |
| Median | 269.85302 |

## Temporal Cortex

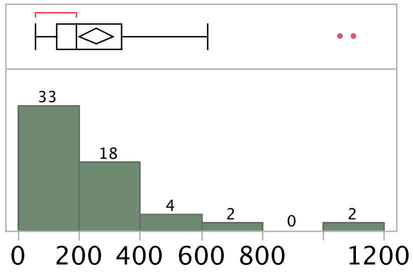


| Summary Statistics | |
|---|---|
| Mean | 252.44311 |
| Std Dev | 216.17428 |
| Std Err Mean | 28.143494 |
| Upper 99.5% Mean | 334.57693 |
| Lower 99.5% Mean | 170.30929 |
| N | 59 |
| Median | 187.04788 |

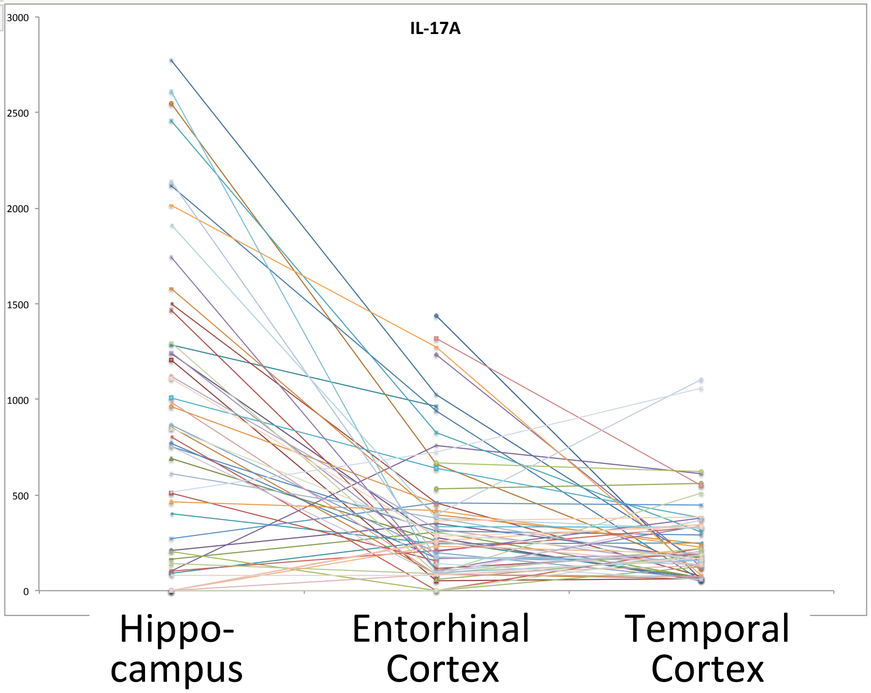

# S. TNF-α

## Hippocampus

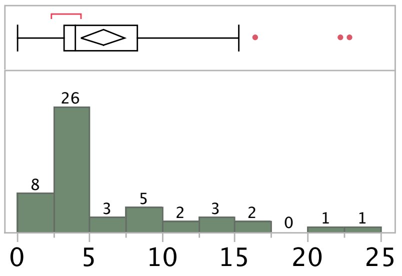


| Summary Statistics | |
|---|---|
| Mean | 5.8935805 |
| Std Dev | 5.3182121 |
| Std Err Mean | 0.7446986 |
| Upper 99.5% Mean | 8.0807337 |
| Lower 99.5% Mean | 3.7064274 |
| N | 51 |
| Median | 4.0127758 |

## Entorhinal Cortex

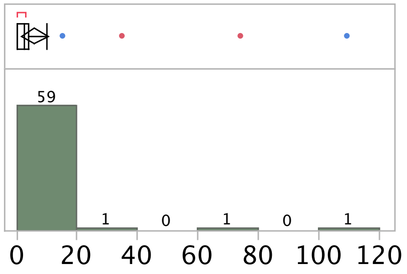


| Summary Statistics | |
|---|---|
| Mean | 6.0244851 |
| Std Dev | 16.912055 |
| Std Err Mean | 2.1478331 |
| Upper 99.5% Mean | 12.280542 |
| Lower 99.5% Mean | −0.231571 |
| N | 62 |
| Median | 2.7473985 |

## Temporal Cortex

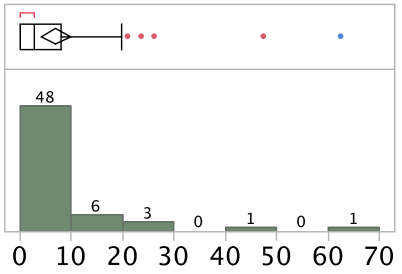


| Summary Statistics | |
|---|---|
| Mean | 6.9439251 |
| Std Dev | 11.121801 |
| Std Err Mean | 1.4479352 |
| Upper 99.5% Mean | 11.169571 |
| Lower 99.5% Mean | 2.718279 |
| N | 59 |
| Median | 2.6231055 |

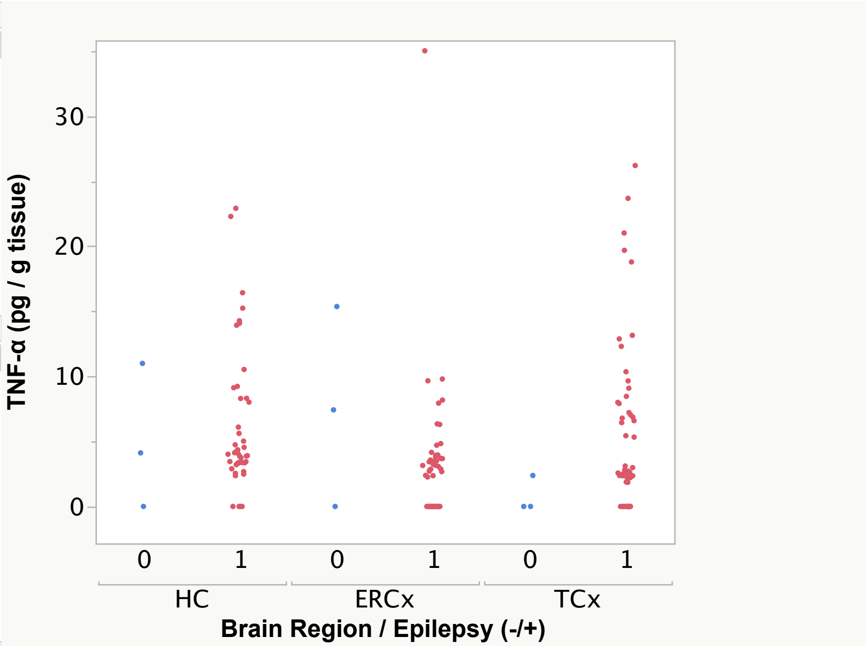


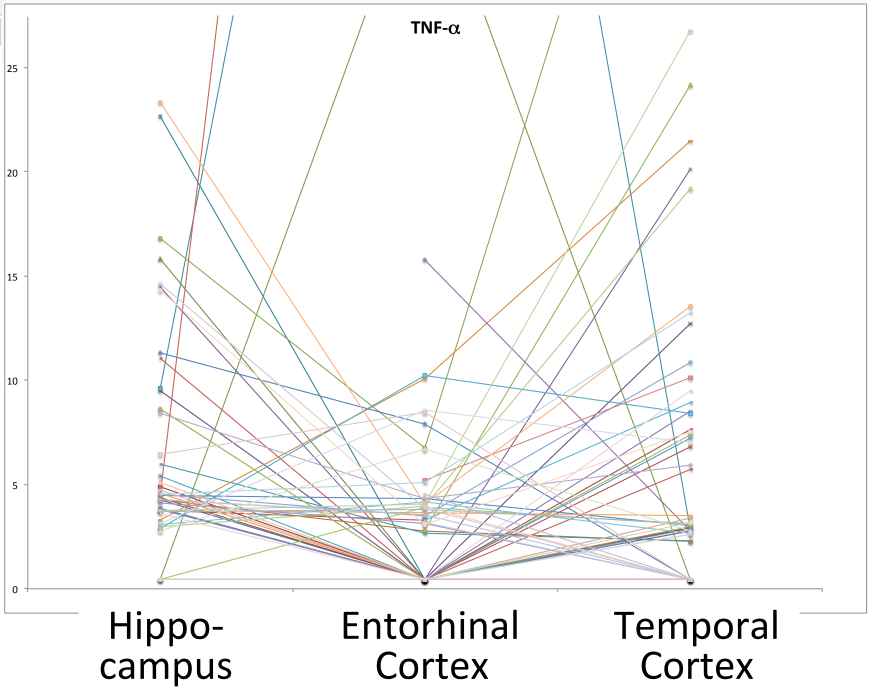


Individual cases where ERCx is **lowest**

| ERCx < HC | ERCx < TCx | **Total** |
|---|---|---|
| 35 | 30 | |
| 69% | 52% | **60%** |

# T. TNF-β

## Hippocampus

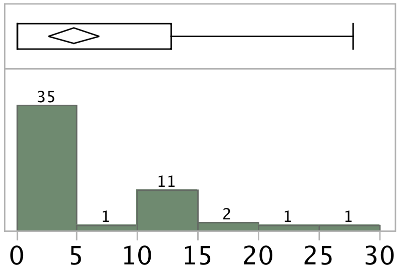

| Summary Statistics | |
|---|---|
| Mean | 4.6765331 |
| Std Dev | 7.4026206 |
| Std Err Mean | 1.0365742 |
| Upper 99.5% Mean | 7.7209143 |
| Lower 99.5% Mean | 1.6321518 |
| N | 51 |
| Median | 0 |

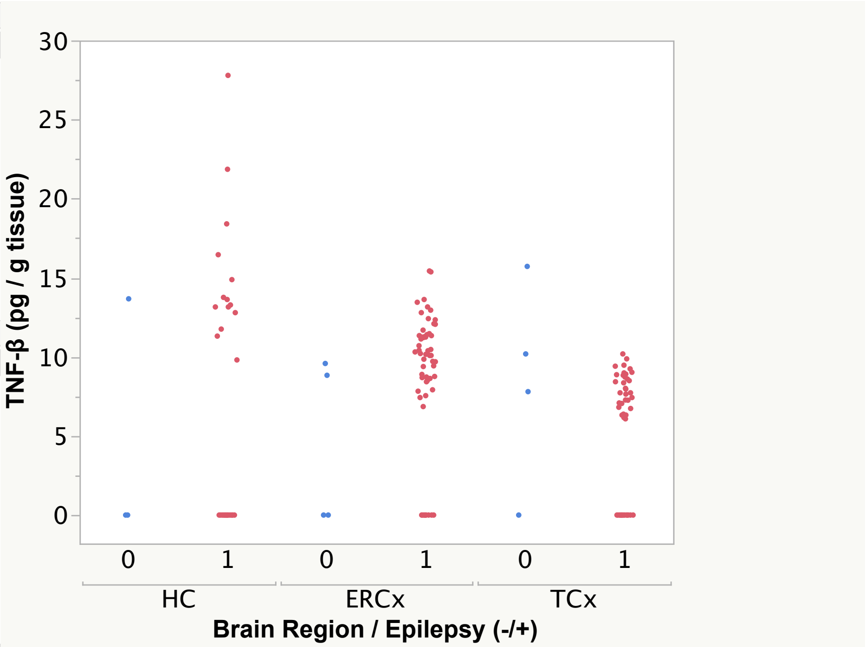


## Entorhinal Cortex

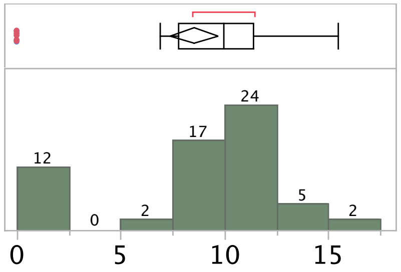

| Summary Statistics | |
|---|---|
| Mean | 8.5208356 |
| Std Dev | 4.5503767 |
| Std Err Mean | 0.5778984 |
| Upper 99.5% Mean | 10.204097 |
| Lower 99.5% Mean | 6.837574 |
| N | 62 |
| Median | 9.9696702 |

## Temporal Cortex

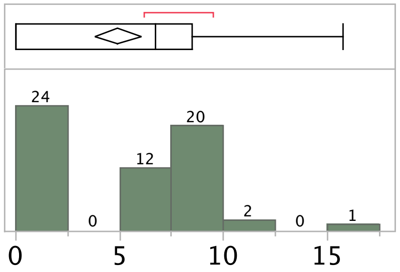

| Summary Statistics | |
|---|---|
| Mean | 4.9002616 |
| Std Dev | 4.3051228 |
| Std Err Mean | 0.5604793 |
| Upper 99.5% Mean | 6.5359611 |
| Lower 99.5% Mean | 3.2645621 |
| N | 59 |
| Median | 6.7361784 |

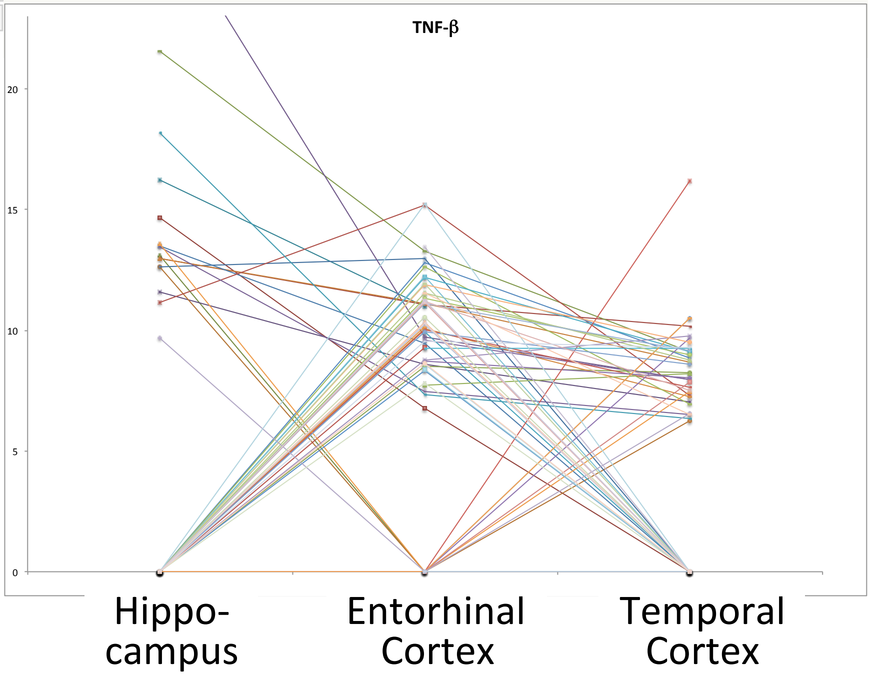


Individual cases where ERCx is **highest**

| ERCx > HC | ERCx > TCx | **Total** |
|---|---|---|
| 37 | 49 | |
| 76% | 83% | **80%** |

# U. IFN-γ

## Hippocampus

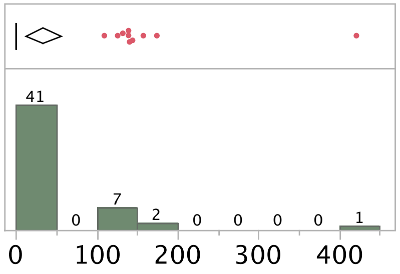


| Summary Statistics | |
|---|---|
| Mean | 33.071243 |
| Std Dev | 77.752997 |
| Std Err Mean | 10.887597 |
| Upper 99.5% Mean | 65.047726 |
| Lower 99.5% Mean | 1.0947612 |
| N | 51 |
| Median | 0 |

## Entorhinal Cortex

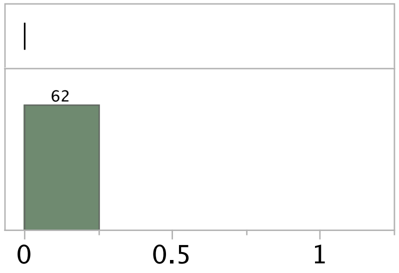


| Summary Statistics | |
|---|---|
| Mean | 0 |
| Std Dev | 0 |
| Std Err Mean | 0 |
| Upper 99.5% Mean | 0 |
| Lower 99.5% Mean | 0 |
| N | 62 |
| Median | 0 |

## Temporal Cortex

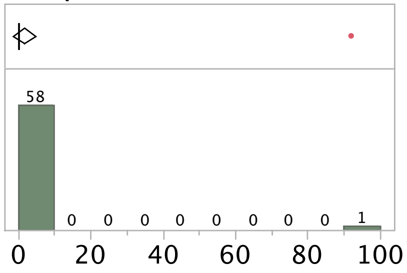


| Summary Statistics | |
|---|---|
| Mean | 1.5624119 |
| Std Dev | 12.001114 |
| Std Err Mean | 1.5624119 |
| Upper 99.5% Mean | 6.1221461 |
| Lower 99.5% Mean | −2.997322 |
| N | 59 |
| Median | 0 |

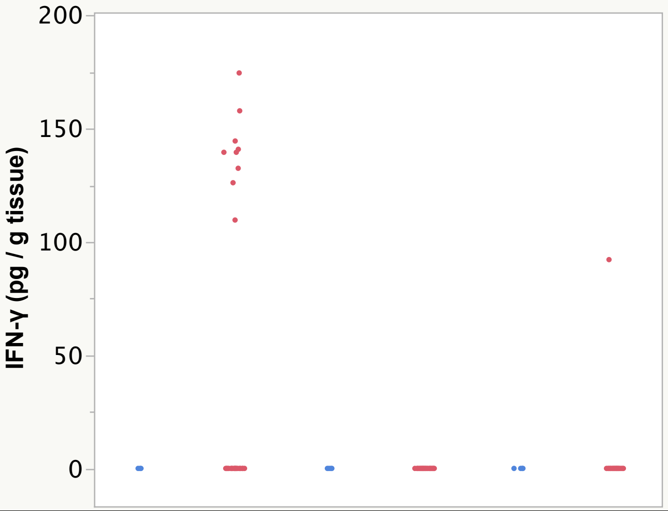


| IFN-γ LEVELS | HIPPOCAMPUS | | ENTORHINAL CORTEX | | TEMPORAL CORTEX | |
|---|---|---|---|---|---|---|
| Non-/Epileptic+ | - | + | - | + | - | + |
| n | 3 | 48 | 4 | 58 | 4 | 55 |
| (pg / g tissue) | **0** | **35.1 ± 11.5** | **0** | **0** | **0** | **1.68 ± 1.68** |

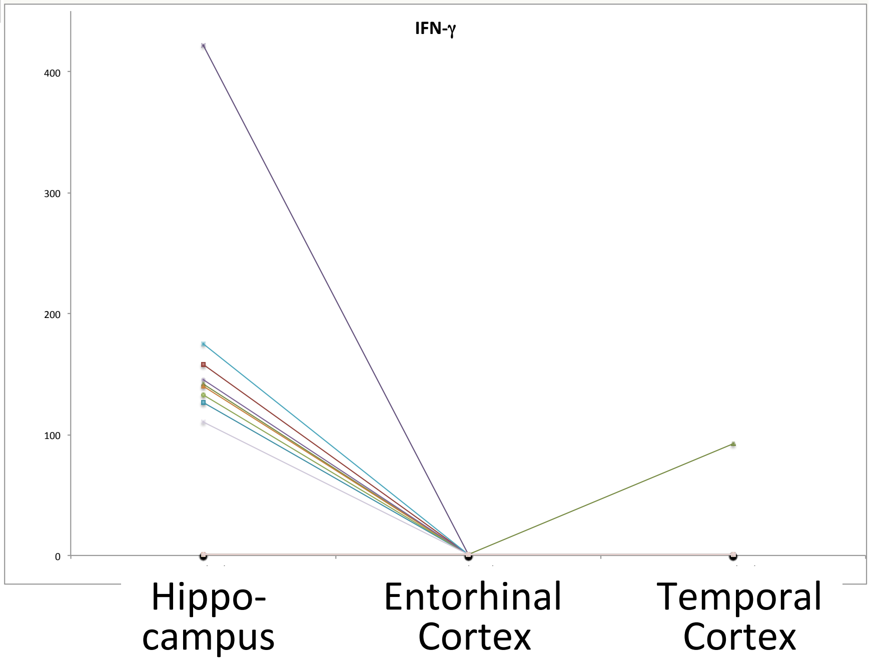

# V. VEGF

## Hippocampus

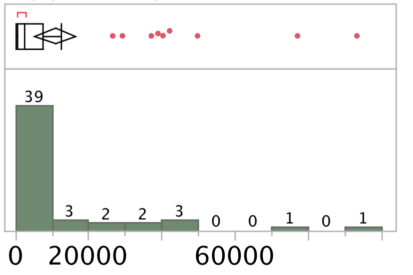


| Summary Statistics | |
|---|---|
| Mean | 10760.95 |
| Std Dev | 19951.405 |
| Std Err Mean | 2793.7555 |
| Upper 99.5% Mean | 18966.109 |
| Lower 99.5% Mean | 2555.7902 |
| N | 51 |
| Median | 2514.8936 |

## Entorhinal Cortex

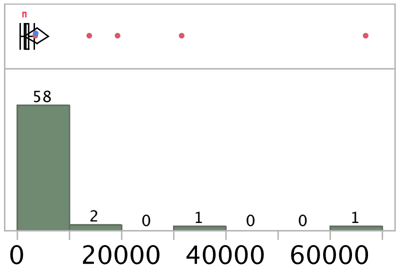


| Summary Statistics | |
|---|---|
| Mean | 3553.3387 |
| Std Dev | 9441.1732 |
| Std Err Mean | 1199.0302 |
| Upper 99.5% Mean | 7045.7891 |
| Lower 99.5% Mean | 60.888255 |
| N | 62 |
| Median | 1417.007 |

## Temporal Cortex

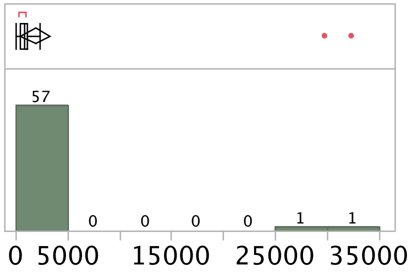


| Summary Statistics | |
|---|---|
| Mean | 1888.8149 |
| Std Dev | 5530.0145 |
| Std Err Mean | 719.94657 |
| Upper 99.5% Mean | 3989.903 |
| Lower 99.5% Mean | -212.2731 |
| N | 59 |
| Median | 871.0026 |

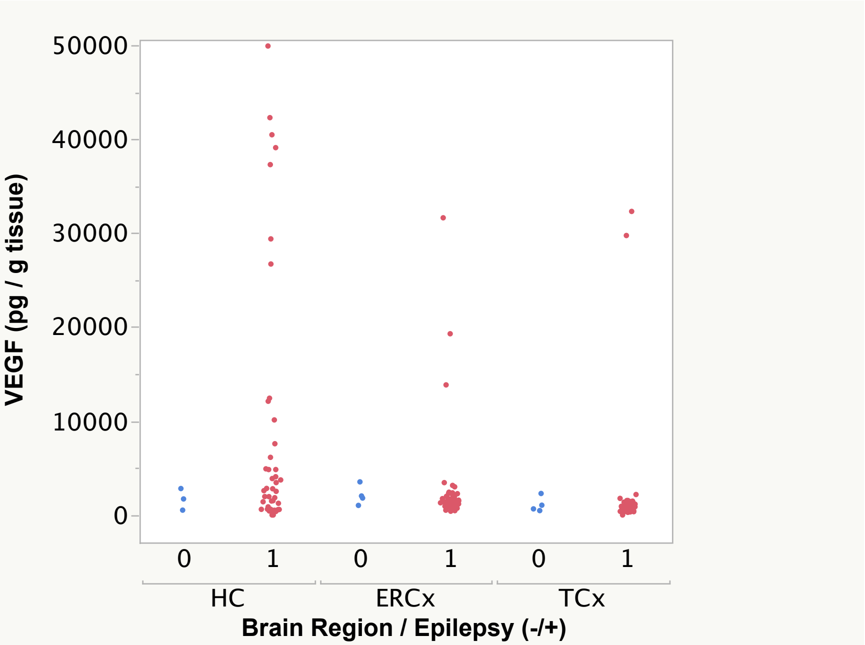


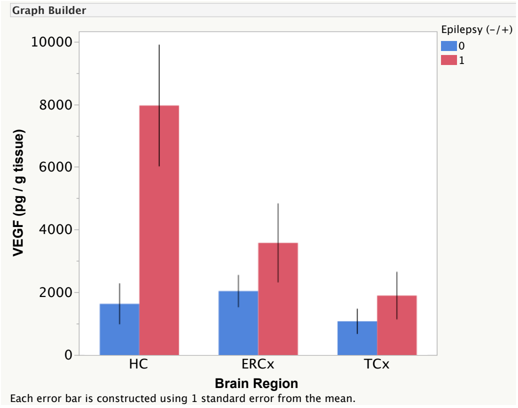


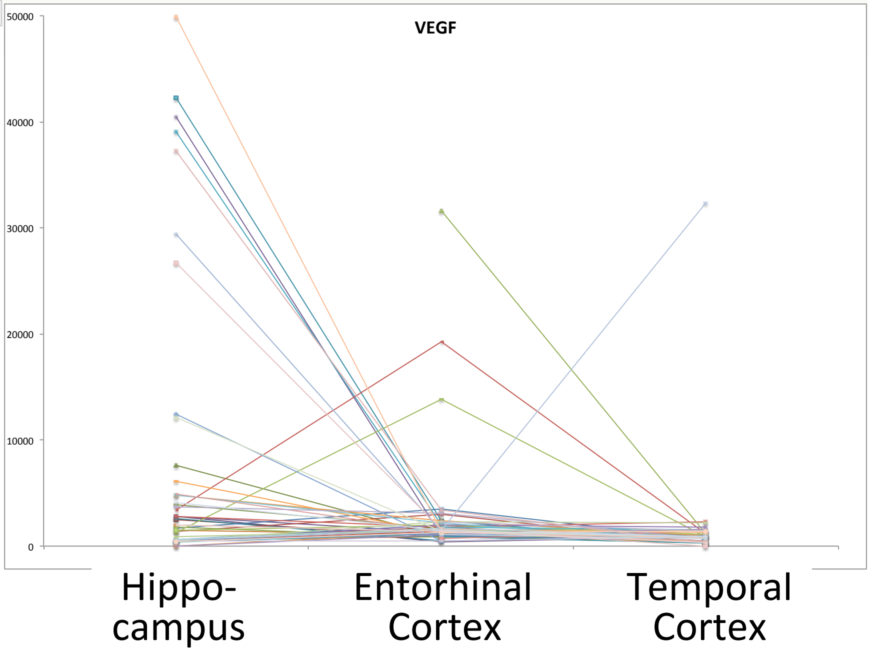

# W. CRP

## Hippocampus

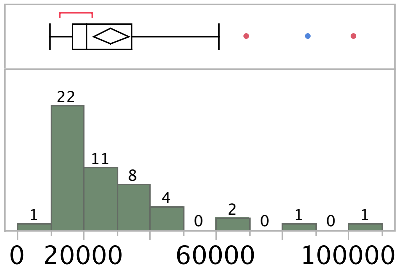


| Summary Statistics | |
|---|---|
| Mean | 28043.072 |
| Std Dev | 18705.723 |
| Std Err Mean | 2645.3888 |
| Upper 99.5% Mean | 35819.8 |
| Lower 99.5% Mean | 20266.344 |
| N | 50 |
| Median | 20676.619 |

## Entorhinal Cortex

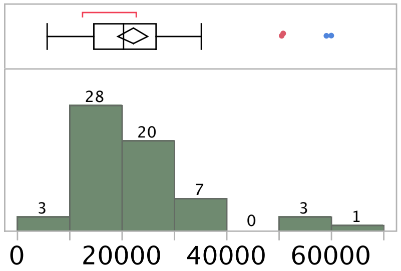


| Summary Statistics | |
|---|---|
| Mean | 22069.276 |
| Std Dev | 11300.121 |
| Std Err Mean | 1435.1168 |
| Upper 99.5% Mean | 26249.383 |
| Lower 99.5% Mean | 17889.17 |
| N | 62 |
| Median | 20093.62 |

## Temporal Cortex

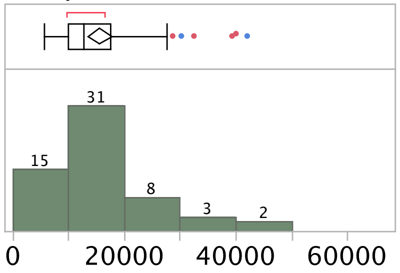


| Summary Statistics | |
|---|---|
| Mean | 15624.415 |
| Std Dev | 8562.1777 |
| Std Err Mean | 1114.7006 |
| Upper 99.5% Mean | 18877.551 |
| Lower 99.5% Mean | 12371.279 |
| N | 59 |
| Median | 12750.651 |

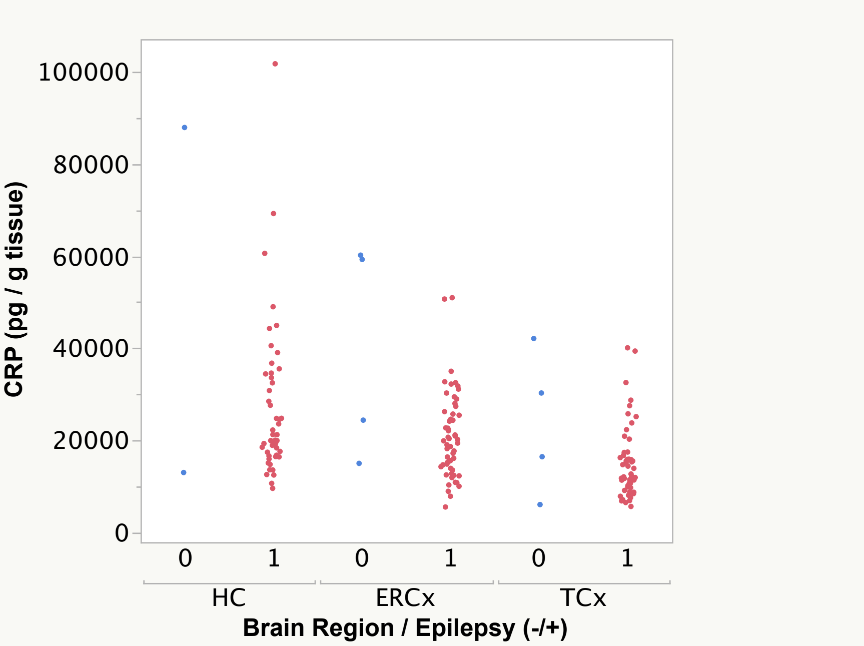


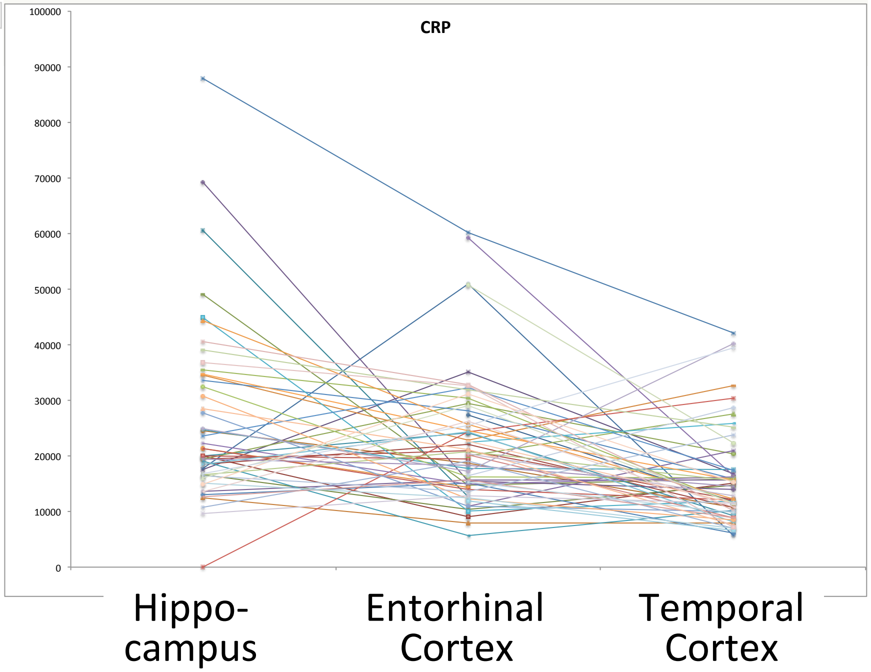

# X. ICAM-1

## Hippocampus

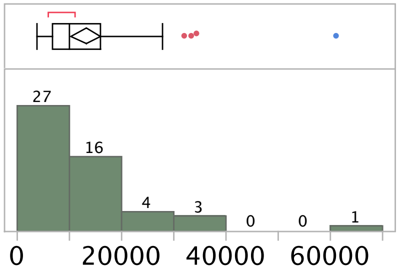


| Summary Statistics | |
|---|---|
| Mean | 13053.495 |
| Std Dev | 10388.719 |
| Std Err Mean | 1454.7117 |
| Upper 99.5% Mean | 17325.931 |
| Lower 99.5% Mean | 8781.0586 |
| N | 51 |
| Median | 9842.5197 |

## Entorhinal Cortex

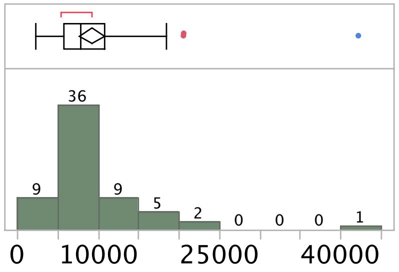


| Summary Statistics | |
|---|---|
| Mean | 9242.5856 |
| Std Dev | 6191.7494 |
| Std Err Mean | 786.35297 |
| Upper 99.5% Mean | 11533.019 |
| Lower 99.5% Mean | 6952.1522 |
| N | 62 |
| Median | 7735.5743 |

## Temporal Cortex

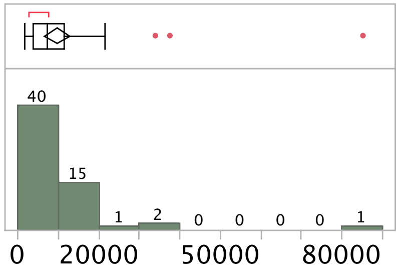


| Summary Statistics | |
|---|---|
| Mean | 9599.2779 |
| Std Dev | 12119.166 |
| Std Err Mean | 1577.781 |
| Upper 99.5% Mean | 14203.865 |
| Lower 99.5% Mean | 4994.6905 |
| N | 59 |
| Median | 7243.9565 |

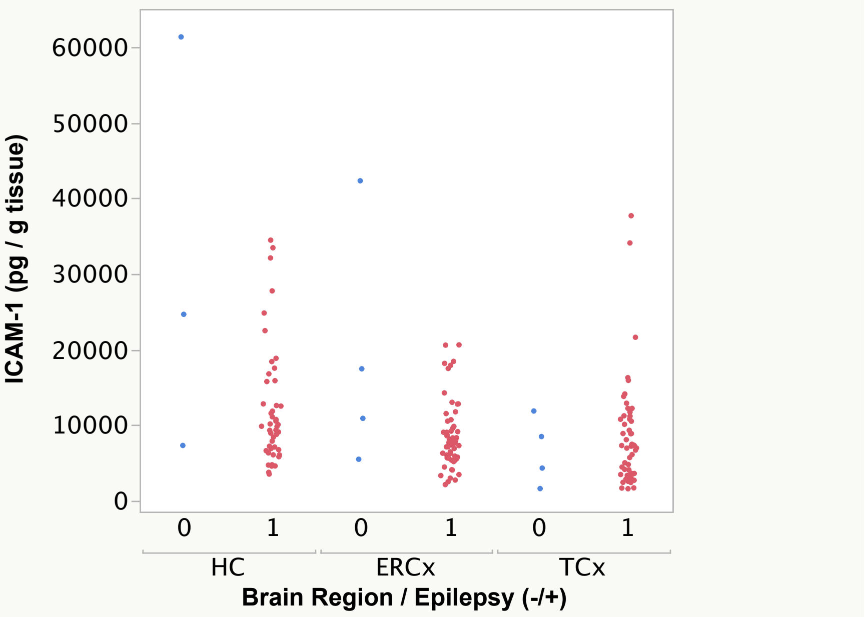


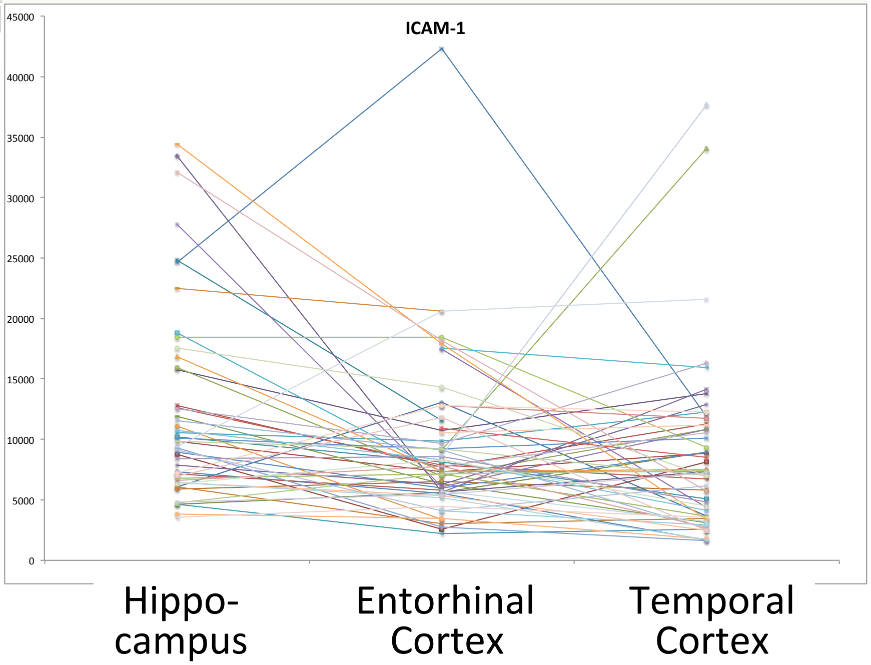

# Y. VCAM-1

## Hippocampus

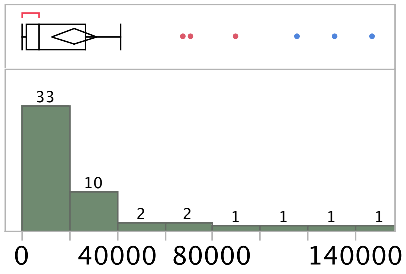


| Summary Statistics | |
|---|---|
| Mean | 21887.106 |
| Std Dev | 33950.217 |
| Std Err Mean | 4753.9813 |
| Upper 99.5% Mean | 35849.378 |
| Lower 99.5% Mean | 7924.8332 |
| N | 51 |
| Median | 7342.8571 |

## Entorhinal Cortex

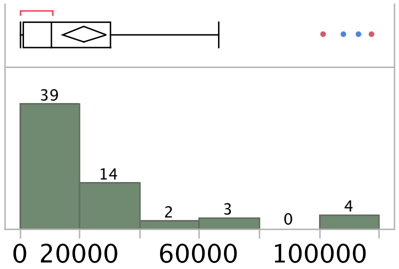


| Summary Statistics | |
|---|---|
| Mean | 21350.517 |
| Std Dev | 28814.645 |
| Std Err Mean | 3659.4636 |
| Upper 99.5% Mean | 32009.544 |
| Lower 99.5% Mean | 10691.489 |
| N | 62 |
| Median | 10324.652 |

## Temporal Cortex

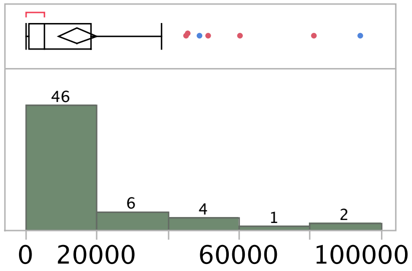


| Summary Statistics | |
|---|---|
| Mean | 14427.377 |
| Std Dev | 20213.632 |
| Std Err Mean | 2631.5908 |
| Upper 99.5% Mean | 22107.396 |
| Lower 99.5% Mean | 6747.3566 |
| N | 59 |
| Median | 5119.3743 |

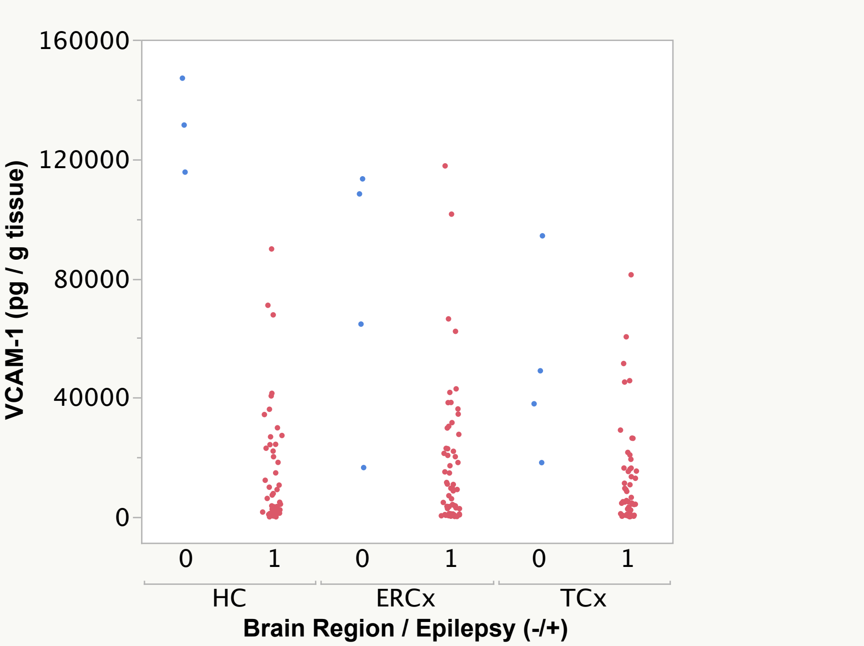


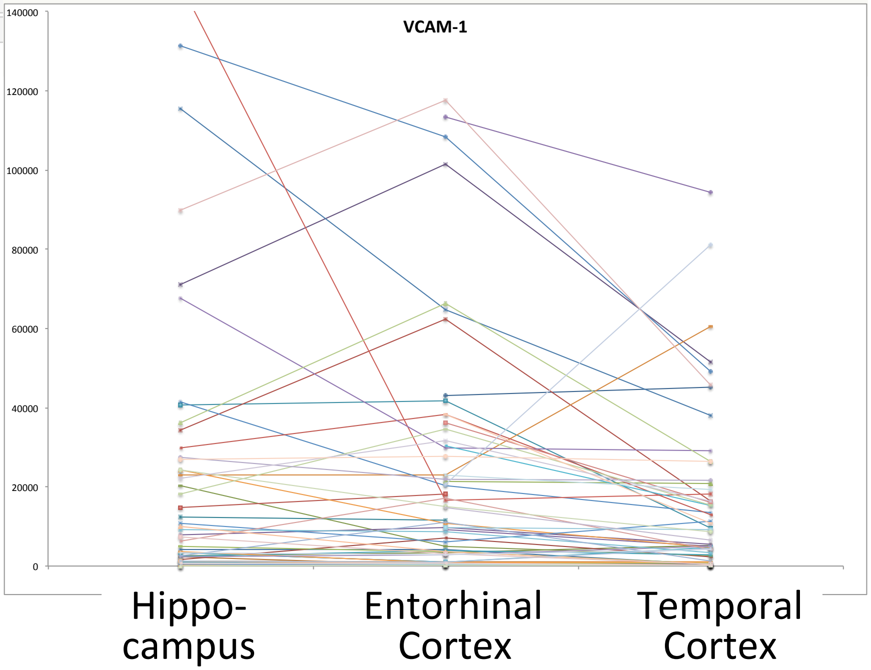

# Z. IL-12 p70:p40 Molar Ratio [See note on ratio calculations, Supp Data 3AA]

## Hippocampus

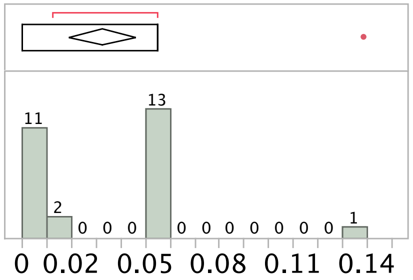


| Summary Statistics | |
|---|---|
| Mean | 0.0323905 |
| Std Dev | 0.0341189 |
| Std Err Mean | 0.0065662 |
| Upper 99.5% Mean | 0.0525284 |
| Lower 99.5% Mean | 0.0122526 |
| N | 27 |
| Median | 0.0548411 |

## Entorhinal Cortex

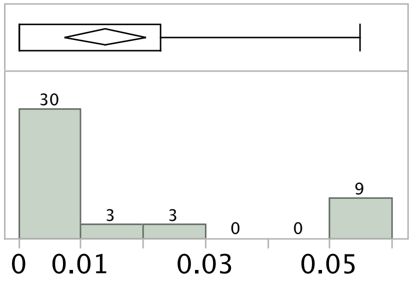


| Summary Statistics | |
|---|---|
| Mean | 0.0139531 |
| Std Dev | 0.0218698 |
| Std Err Mean | 0.0032602 |
| Upper 99.5% Mean | 0.0235886 |
| Lower 99.5% Mean | 0.0043176 |
| N | 45 |
| Median | 0 |

## Temporal Cortex

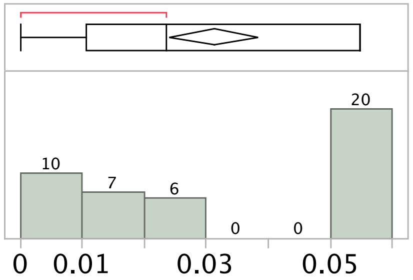


| Summary Statistics | |
|---|---|
| Mean | 0.0312585 |
| Std Dev | 0.0233748 |
| Std Err Mean | 0.0035646 |
| Upper 99.5% Mean | 0.0418204 |
| Lower 99.5% Mean | 0.0206967 |
| N | 43 |
| Median | 0.0234773 |

| MOLAR RATIO | | Hippocampus | Entorhinal Cx | Temporal Cx |
|---|---|---|---|---|
| **IL-12 p70:p40** | Mean | 0.032 | 0.014 | 0.031 |
| | Std Err | 0.007 | 0.003 | 0.004 |
| | N | 27 | 45 | 43 |
| **IL-12 p40:p70** | Mean | 13.2 | 22.6 | 31.5 |
| | Std Err | 3.8 | 5.2 | 5.7 |
| | N | 32 | 25 | 42 |

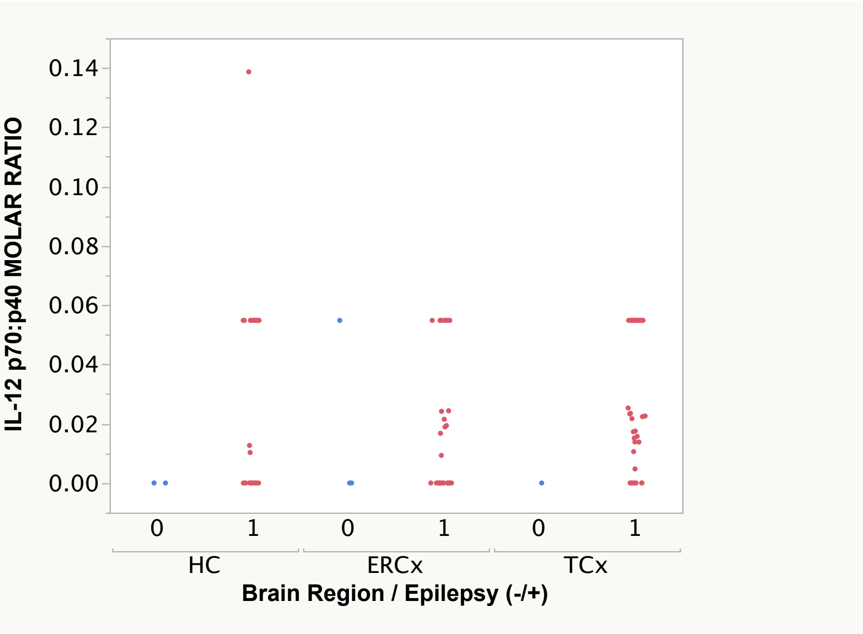


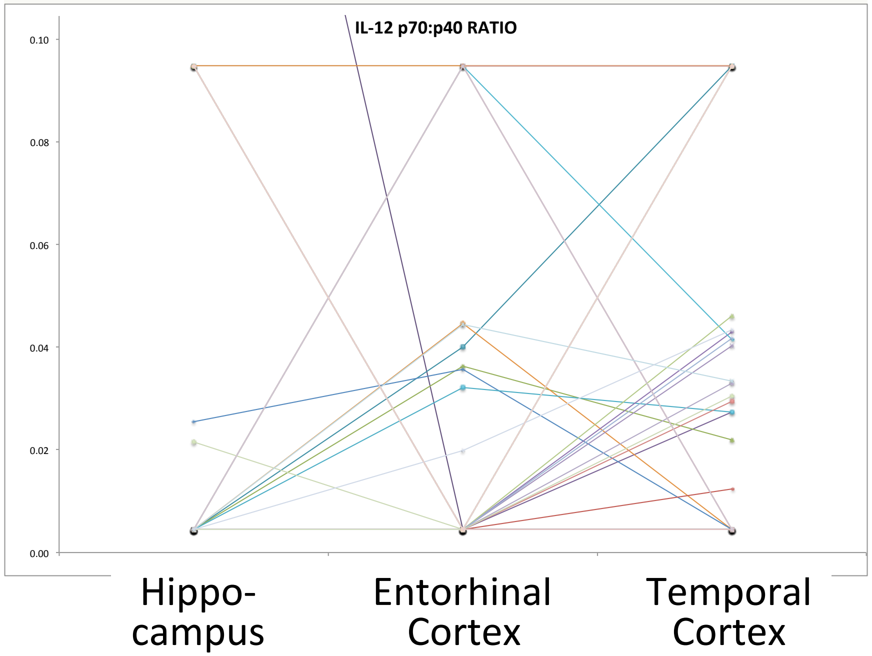

# AA. IL-12 p40:p70 Molar Ratio

## Hippocampus

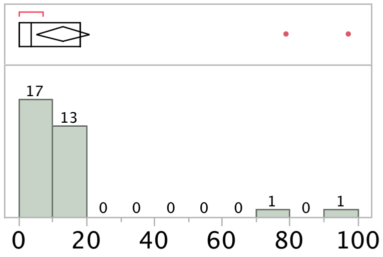


**Summary Statistics**

| | |
|---|---|
| Mean | 13.142482 |
| Std Dev | 21.669841 |
| Std Err Mean | 3.8307229 |
| Upper 99.5% Mean | 24.719378 |
| Lower 99.5% Mean | 1.5655863 |
| N | 32 |
| Median | 3.6056797 |

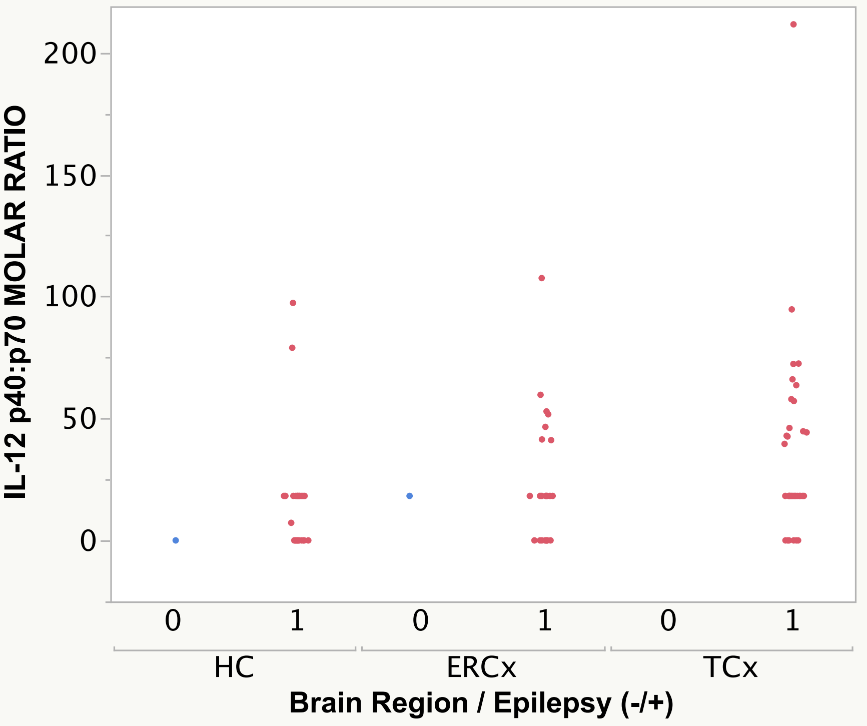


## Entorhinal Cortex

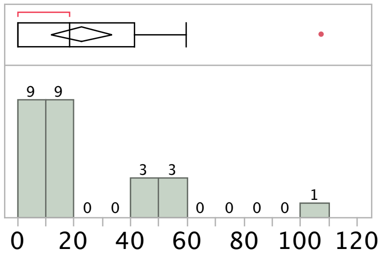


**Summary Statistics**

| | |
|---|---|
| Mean | 22.594093 |
| Std Dev | 26.126967 |
| Std Err Mean | 5.2253935 |
| Upper 99.5% Mean | 38.743243 |
| Lower 99.5% Mean | 6.4449439 |
| N | 25 |
| Median | 18.234493 |

## Temporal Cortex

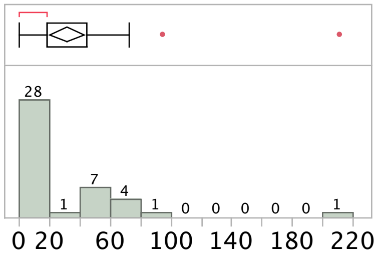


**Summary Statistics**

| | |
|---|---|
| Mean | 31.438186 |
| Std Dev | 36.838506 |
| Std Err Mean | 5.6843048 |
| Upper 99.5% Mean | 48.303298 |
| Lower 99.5% Mean | 14.573073 |
| N | 42 |
| Median | 18.234493 |

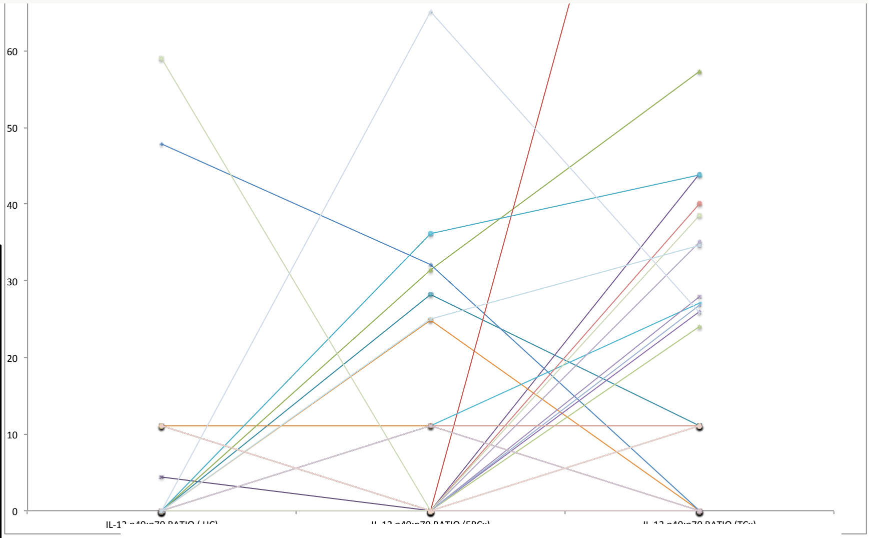


Hippo-campus Entorhinal Cortex Temporal Cortex

IL-12 p40 and p70 molar ratios were deduced by taking the mass ratio and dividing by the molecular weight ratio, based upon amino acid sequences published in the GenBank database. Accession numbers for human p40: AF180563_1, 34,694 daltons; human p35: AF180562_1, 22,540 daltons; thus, estimated a value for p70 of 57,234 daltons.

3. Mediator Variability Measures

**Table S3B. Brain Inflammatory-related Mediator Relative Tissue Concentrations**

| | MEDIATOR | MEDIAN (pg / g tissue) | MEAN (pg / g tissue) | *C.V. (%)* |
|---|---|---|---|---|
| > 5 ng / g tissue | **CRP** | 17490 | 21121 | *60* |
| | **VCAM-1** | 8203 | 19135 | *146* |
| | **ICAM-1** | 7885 | 9755 | *72* |
| 1 – 5 ng / g tissue | **MCP-1** | 4170 | 6942 | *139* |
| | **IP-10** | 2987 | 5700 | *157* |
| | **IL-1α** | 1752 | 2481 | *99* |
| | **MIP-1β** | 1364 | 2429 | *107* |
| | **VEGF** | 1268 | 4175 | *236* |
| 0.1 – 1 ng / g tissue | **MCP-4** | 495 | 677 | *114* |
| | **MIP-1α** | 442 | 1262 | *158* |
| | **TARC** | 358 | 672 | *157* |
| | **Eotaxin** | 340 | 437 | *90* |
| | **IL-8** | 299 | 1008 | *195* |
| | **IL-17A** | 280 | 499 | *113* |
| 5 – 100 pg/ g tissue | **IL-12/23p40** | 61.4 | 87.1 | *117* |
| | **IL-1β** | 19.1 | 27.6 | *114* |
| | **IL-6** | 8.67 | 43.2 | *195* |
| | **TNF-β** | 7.49 | 5.92 | *91* |
| | **IL-4** | 7.11 | 8.22 | *69* |
| 0 – 10 pg / g tissue | **IL-12p70** | 4.53 | 3.71 | *99* |
| | **TNF-α** | 3.19 | 4.70 | *122* |
| | **GM-CSF** | 0 | 1962 | *185* |
| | **IL-2** | 0 | 15.3 | *232* |
| | **IFN-γ** | 0 | 10.3 | *436* |
| | **IL-10** | 0 | 2.95 | *183* |

### Table S3C – F. Mediator Levels by Coefficients of Variation (C.V.)

| | S3C. Overall C.V. | S3D. Variation by Tissue | | | S3E. Variation by Epilepsy Status | | |
|---|---|---|---|---|---|---|---|
| MEDIATOR | Mean C.V. (%) | HC | ERCx | TCx | MEDIATOR | Nonepileptic, 0 | Epileptic, 1 |
| VEGF | 235.5 | 169.2 | 265.7 | 292.8 | VEGF * | 61.3 | 233.1 |
| IL-2 | 231.5 | 143.9 | 346.0 | 218.4 | IL-2 | 182.6 | 237.0 |
| IL-6 | 195.2 | 174.6 | 188.2 | 188.8 | IL-6 * | 81.1 | 208.3 |
| IL-8 | 194.7 | 151.0 | 209.9 | 171.0 | IL-8 * | 72.8 | 208.0 |
| IL-10 | 182.5 | 126.3 | 246.0 | 194.2 | IL-10 | 198.6 | 181.7 |
| MIP-1α | 157.6 | 133.8 | 165.3 | 126.7 | MIP-1α | 142.3 | 159.1 |
| IP-10 | 156.8 | 89.9 | 159.8 | 155.0 | IP-10 | 150.5 | 151.1 |
| TARC | 156.5 | 119.5 | 75.7 | 184.4 | TARC | 152.7 | 156.7 |
| VCAM-1 | 146.3 | 155.1 | 135.0 | 140.1 | VCAM-1 | 56.7 | 137.6 |
| MCP-1 | 139.3 | 106.4 | 79.2 | 162.7 | MCP-1 | 63.3 | 143.4 |
| TNF-α | 122.4 | 90.2 | 165.0 | 121.4 | TNF-α | 126.0 | 122.6 |
| IL-12/23p40 | 117.1 | 144.7 | 102.0 | 114.8 | IL-12/23p40 | 124.4 | 115.8 |
| IL-1β | 114.2 | 111.6 | 109.9 | 95.9 | IL-1β | 115.2 | 110.9 |
| MCP-4 | 114.1 | 75.9 | 50.6 | 169.4 | MCP-4 | 58.1 | 116.9 |
| IL-17A | 113.1 | 82.4 | 92.1 | 85.6 | IL-17A | 114.8 | 113.3 |
| MIP-1β | 106.8 | 93.9 | 99.4 | 101.6 | MIP-1β | 113.6 | 106.0 |
| IL-1α | 98.99 | 116.7 | 80.9 | 80.0 | IL-1α | 92.8 | 98.6 |
| IL-12p70 | 98.86 | 93.2 | 126.5 | 61.3 | IL-12p70 | 211.4 | 95.1 |
| TNF-β | 91.02 | 162.3 | 53.4 | 87.9 | TNF-β | 102.4 | 90.5 |
| Eotaxin | 90.23 | 76.4 | 78.3 | 105.2 | Eotaxin | 81.9 | 88.0 |
| ICAM-1 | 72.41 | 65.0 | 67.0 | 82.4 | ICAM-1 | 90.3 | 69.5 |
| IL-4 | 69.27 | 71.8 | 43.8 | 52.5 | IL-4 | 85.5 | 68.3 |
| CRP | 59.77 | 58.6 | 51.2 | 54.8 | CRP | 74.3 | 53.3 |
| GM-CSF | - | | | | | | |
| IFN-γ | - | | | | | | |
| | | Means | | | | Means | |
| | | **113.6** | **130.0** | **132.5** | | **111.0** | **133.3** |
| **Grand Mean** | **125%** | (105, | 146.2) | 99.5% C.I. | | | |

C.V. = (sample standard deviation) / (sample mean) • 100%. Underlined C.V.'s indicate above the 99.5% confidence interval between all mediators. "–" indicates an incalculable C.V. due to insufficient measurable values among the tissue samples. *Coefficients of variation for these mediators in hippocampus (HC), entorhinal cortex (ERCx) and temporal cortex (TCx) were 1.5- to 7-fold greater among the epileptic cases compared to nonepileptic cases.

**Table S3F. Mediator Coefficients of Variation by Tissue and Epilepsy Status**

| Mediator | HC, 0 | HC, 1 | ERCx, 0 | ERCx, 1 | TCx, 0 | TCx, 1 |
|---|---|---|---|---|---|---|
| **VEGF** | 68.5 | **165.1** | 50.1 | **267.0** | 73.9 | **294.2** |
| **IL-2** | 173.2 | 143.1 | 119.5 | 393.4 | 200.0 | 145.7 |
| **IL-6** | 44.6 | **191.9** | 126.5 | **195.7** | 98.8 | **193.3** |
| **IL-8** | 19.2 | 130.0 | 111.1 | 220.5 | 108.7 | 173.5 |
| **IL-10** | 173.2 | 124.1 | 200.0 | 248.9 | 200.0 | 195.7 |
| **MIP-1α** | 122.7 | 134.1 | 133.5 | 127.7 | 100.2 | 124.4 |
| **IP-10** | 62.6 | 92.6 | 125.4 | 138.2 | 137.3 | 157.7 |
| **TARC** | 50.0 | 119.6 | 67.9 | 71.7 | 189.8 | 185.2 |
| **VCAM-1** | 12.0 | **133.4** | 59.6 | **134.4** | 64.6 | **141.6** |
| **MCP-1** | 18.5 | 112.9 | 75.7 | 79.8 | 97.6 | 162.4 |
| **TNF-α** | 110.3 | 90.1 | 101.2 | 172.3 | 173.2 | 117.4 |
| **IL-12/23p40** | 113.4 | 149.3 | 128.0 | 98.9 | 169.4 | 111.4 |
| **IL-1β** | 91.0 | 112.0 | 89.4 | 108.8 | 158.9 | 88.8 |
| **MCP-4** | 17.4 | 77.9 | 41.4 | 49.8 | 113.9 | 169.1 |
| **IL-17A** | 103.1 | 82.3 | 83.3 | 92.0 | 71.5 | 85.5 |
| **MIP-1β** | 83.9 | 94.9 | 53.5 | 102.8 | 122.2 | 99.7 |
| **IL-1α** | 67.1 | 119.7 | 99.5 | 79.2 | 95.2 | 79.6 |
| **IL-12p70** | 173.2 | 90.5 | 200.0 | 124.0 | | 55.1 |
| **TNF-β** | 173.2 | 163.8 | 115.7 | 50.3 | 77.4 | 87.6 |
| **Eotaxin** | 40.3 | 79.1 | 71.9 | 64.6 | 143.8 | 92.5 |
| **ICAM-1** | 76.6 | 65.2 | 85.3 | 52.1 | 68.5 | 83.0 |
| **IL-4** | 70.5 | 71.5 | 39.6 | 44.2 | 145.2 | 34.9 |
| **CRP** | 104.8 | 50.4 | 58.9 | 44.1 | 66.3 | 51.4 |

| | | |
|---|---|---|
| (92, | 131.8) | 99.5% C.I. |

Of the 9 mediators that showed highest variability (Supplemental Data **3C**), only VEGF, IL-6, and VCAM-1 showed increased variation across all 3 brain regions only in epileptic cases. These deviations might be attributed to the larger number of observations in the epileptic cases, however, though the numbers of observations were comparable, for 10 of 23 measurable mediators, the C.V.'s across all tissues and epilepsy status were inside the 99.5% CI. Only these 3 mediators showed consistently higher coefficients of variation in all 3 brain regions of epilepsy cases. No mediator(s) showed increased variability across all 3 brain regions of nonepileptic cases.

## Table S3G. GM-CSF Homogeneity Analyses

| Brain Region | Group | Outcomes n (%) | |
|---|---|---|---|
| **A. Epilepsy Status** | | | |
| | | **GM-CSF +** | **GM-CSF –** |
| | Nonepileptic | 4 (36%) | 7 (64%) |
| | Epileptic | 40 (25%) | 121 (75%) |
| $\chi^2$ (with Yate's correction) | | 0.2401 (df = 1, p = 0.380) | |
| **B. Brain Region** | | | |
| | | **GM-CSF +** | **GM-CSF –** |
| Hippocampus | | 15 (29%) | 36 (71%) |
| Entorhinal Cortex | | 4 (7%) | 58 (93%) |
| Temporal Cortex | | 25 (42%) | 34 (58%) |
| $\chi^2$ (with Yate's correction) | | 19.03 (df = 2, p < 0.0001) | |
| **C. Brain Region + Epilepsy** | | **GM-CSF +** | **GM-CSF –** |
| Hippocampus | Nonepileptic | 0 (0) | 3 (100%) |
| Hippocampus | Epileptic | 15 (31%) | 33 (69%) |
| Entorhinal Cortex | Nonepileptic | 1 (25%) | 3 (75%) |
| Entorhinal Cortex | Epileptic | 3 (5%) | 55 (95%) |
| Temporal Cortex | Nonepileptic | 3 (75%) | 1 (25%) |
| Temporal Cortex | Epileptic | 22 (40%) | 33 (60%) |
| $\chi^2$ (with Yate's correction) | | 20.74 (df = 5, p = 0.0008) | |

CONCLUSIONS

**For GM-CSF**:
A. there is homogeneity in GM-CSF levels between nonepileptic and epileptic brains;

B. there is NON-homogeneity in GM-CSF levels between the brain regions examined;

C. there is NON-homogeneity in GM-CSF levels between different brain regions AND epilepsy status.

## S3H. IFN-γ Homogeneity Analyses

| Brain Region | Group | Outcomes n (%) | |
|---|---|---|---|
| **A. Epilepsy Status** | | | |
| | | **IFN-γ +** | **IFN-γ –** |
| | Nonepileptic | 0 (0) | 11 (100%) |
| | Epileptic | 10 (6%) | 151 (94%) |
| $\chi^2$ (with Yate's correction) | | 0.6765 (df = 1, p > 0.05) | |
| **B. Brain Region** | | | |
| | | **IFN-γ +** | **IFN-γ –** |
| Hippocampus | | 9 (18%) | 42 (82%) |
| Entorhinal Cortex | | 0 (0) | 62 (100%) |
| Temporal Cortex | | 1 (2%) | 58 (98%) |
| $\chi^2$ (with Yate's correction) | | 14.96 (df = 2, p = 0.0006) | |
| **C. Brain Region + Epilepsy** | | **IFN-γ +** | **IFN-γ –** |
| Hippocampus | Nonepileptic | 0 (0) | 3 (100%) |
| Hippocampus | Epileptic | 9 (23%) | 39 (77%) |
| Entorhinal Cortex | Nonepileptic | 0 (0) | 4 (100%) |
| Entorhinal Cortex | Epileptic | 0 (0) | 58 (100%) |
| Temporal Cortex | Nonepileptic | 0 (0) | 4 (100%) |
| Temporal Cortex | Epileptic | 1 (2%) | 53 (98%) |
| $\chi^2$ (with Yate's correction) | | 17.07 (df = 5, p = 0.004) | |

**For Interferon-γ**:
A. there is homogeneity in IFN-γ levels between nonepileptic and epileptic brains;

B. there is NON-homogeneity in IFN-γ levels between the brain regions examined;

C. there is NON-homogeneity in IFN-γ levels between different brain regions AND epilepsy status.

## 4. Miscellaneous Supplemental Material

### Table S4I. Phase-2 : Brain Inflammatory-related Mediator Associations

Eotaxin, IL-1$\alpha$, MCP-1, and MCP-4 showed overall greater levels in cases with prior electrode placement (n = 31), compared to cases with no prior neurosurgical intervention (n = 31). VEGF levels among the Phase-2 (-) group were greater in the hippocampus than in cortical regions.

### Table S4J-K. Inflammatory-related Mediator Brain & Consensus Blood Levels

Comparisons of median brain and estimated blood levels of inflammatory-related mediators (table of citations referenced in **Table S4J,** below)

### Supplemental Figure 4A. Age-Related Correlations

Upper Row: VCAM-1 levels in human brain; hippocampus correlated to age at surgery ($p < 0.02$). Lower Row: IL-8 levels in human brain; hippocampus correlated to age at surgery ($p < 0.05$). Due to age differences between epileptic and nonepileptic groups (**Table 1A**), and as a test of robustness, correlation analyses were repeated, excluding the older nonepileptics. No significant correlations were observed in this sub-analysis, thus, the older nonepileptic cases greatly influenced the correlation of hippocampal VCAM-1 and IL-8 with age.

### Supplemental Figure 4B. Epilepsy Risk Factor Associations

VEGF: mCHI subgroup > All subgroups (p = 0.0001); hippocampus- hippocampus,mCHI > hippocampus,All subgroups (p < 0.005 for all except hypoxic/ischemic p < 0.035)

IL-12/23 p40: Infection > Febrile, CHI (p = 0.035); ENTORHINAL CORTEX- entorhinal cortex,Infection > entorhinal cortex[CHI, Devel, Cancer] (p < 0.0004 for all)

IP-10: Infection > All subgroups (p = 0.0002); entorhinal cortex,Infection > entorhinal cortex,All subgroups (p < 0.035 for all)

IL-1$\alpha$: Infection > CHI, Febrile (p = 0.041)

MIP-1$\beta$: mCHI > Febrile, Devel, Infection, Cancer (p < 0.002, < 0.02, < 0.02, < 0.04, respectively)

IL-6: CHI > Febrile (p < 0.03)

### Supplemental Figure 4C. Relational Correlations of Inflammatory-related Mediators Between Tissues

Boxes and horizontal lines indicate cross-tissue correlations, *italics* show connections between relational groups.

**Table S4I. Phase-2 and Brain Inflammation-related Mediator Associations**

| MEDIATOR | *Over All Brain Regions*[1] | By Brain Region[2] | | |
|---|---|---|---|---|
| | | *Hippo-campus* | *Entorhinal Cortex* | *Temporal Cortex* |
| **Eotaxin** | (+) > (-) | - | (+) > (-)* | (+) > (-)* |
| **MCP-1** | (+) > (-) | - | (+) > (-)[§] | (+) > (-)[§] |
| **MCP-4** | (+) > (-) | - | (+) > (-)[§] | (+) > (-)[§] |
| **IL-1α** | (+) > (-) | - | (+) > (-)[a] | (+) > (-)[a] |
| **CRP** | (+) > (-) | - | - | (+) > (-)* |
| **ICAM-1** | (+) > (-) | - | - | (+) > (-)[§] |

(+) indicates the group of epilepsy cases with electrode placement prior to resection, (-) indicates cases with no electrode placement. Results of Phase-2 × Brain Region ANOVA: [1]Phase-2 effect $p < 0.05$ post hoc Tukey HSD; [2] Phase-2 × Brain Region interaction, *$p \leq 0.01$, [§]$p < 0.05$, [a]$p < 0.10$ post hoc Tukey HSD. HC = hippocampus, ERCx = entorhinal cortex, TCx = temporal cortex.

# Table S4J. MEDIATOR LEVEL COMPARISONS

| MEDIATOR | BRAIN (median) (pg / g tissue) | BLOOD (pg / mL) | CSF (pg / mL) | BLOOD & CSF REFERENCES estimated consensus levels (see Table S4J for citations) |
|---|---|---|---|---|
| Eotaxin | 340 | 70 - 300 | 0 - 16 | Heidigger 2015, Agarwal 2013, Hashimoto 2006, Rosa 2008, Hang 2014, Comar 2014, Kothur 2016, Yoshio 2016 |
| IP-10 | 2987 | 50 - 1000 | 45 - 4000 | Romagnani 2002, Simmons 2013, Xu 2005, Purohit 2015, Hang 2014, Comar 2014, Kothur 2016, Yoshio 2016 |
| MCP-1 | 4170 | 165 - 1000 | 65 - 820 | Garcia-Alonso 2009, Floris-Moore 2009, Purohit 2015, Pawlak 2006, Hang 2014, Dworacka 2014, Fontes2015, Schnabel 2008, Kothur 2016, Yoshio 2016 |
| MCP-4 | 495 | 24 - 350 | | Breland 2010, Hashimoto 2006 |
| MIP-1α | 442 | 3 - 600 | 0 - 16 | Zinyama-Gutsire 2009, Terpos 2005, Pawlak 2006, Comar 2014, Quirico-Santos 2013, Kothur 2016, Yoshio 2016 |
| MIP-1β | 1364 | 4 - 225 | 4 - 27 | Flammand 2001, Pawlak 2006, Hang 2014, Kothur 2016, Yoshio 2016 |
| TARC | 358 | 10 - 720 | | Miyazaki 2007, Feng 2015 |
| GM-CSF | 0 | 0.5 - 75 | 180 - 444 | Bilgici 2014, Surendar 2012, Navarro-Sobrino 2009, Szaryska 2015, Kothur 2016 |
| IFN-γ | 0 | 0 - 230 | 0 - 40 | Resende 2012 , Nishida 2015, Uyanik 2015, Grant 2012, Comar 2014, Kothur 2016, Yoshio 2016 |
| IL-1α | 1752 | 2 - 800 | | Rosa 2008, Jung 2010, Karamehic 2008, Chao 2008, Comar 2014, |
| IL-1β | 19 | 0.1 - 1150 | 0.03 - 13 | Surendar 2012, Rosa 2008, Alapirtti 2009, Grant 2012, Uludag 2013, Bauer 2009, Quirico-Santos 2013, Nowak 2011, Kothur 2016, Yoshio 2016 |
| IL-2 | 0 | 0 - 37 | 0 - 5.1 | Petrikis 2015, Ichinose 2015, Grant 2012, Kothur 2016, Yoshio 2016 |
| IL-4 | 7 | 1 - 400 | 0 - 3.7 | Rosa 2008, Uyanik 2015, Zhang 2015, Nishida 2015, El-Kady 2005, Comar 2014, Kothur 2016, Yoshio 2016 |
| IL-6 | 9 | 2 -51 | 1.3 - 800 | Xu 2005, Shiah 2005, Surendar 2012, Nishida 2015, Uyanik 2015, Rosa 2008, Chao 2008, Billiau 2007, Ichiyama 2008, Quirico-Santos 2013, Petrikis 2015, Sayed 2015, Grant 2012, Comar 2014, Fontes2015 , Schnabel 2008, Uludag 2013, Bauer 2009, Nowak 2011, Kothur 2016, Yoshio 2016 |
| IL-8 | 299 | 3 - 61 | 5 - 60 | Sayed 2015, Nishida 2015, Billiau 2007Hang 2014 , Grant 2012, Tylman 2011, Comar 2014, Siregar 2015, Kothur 2016, Yoshio 2016 |
| IL-10 | 0 | 3 - 60 | 0.1 - 4.4 | Chao 2008, Billiau 2007, Rosa 2008, Uyanik 2015, Szaryska 2015, Grant 2012, Kothur 2016, Yoshio 2016 |
| IL-12/23p40 | 61 | 1 - 230 | | Comar 2014 |
| IL-12p70 | 5 | 1 - 14 | 0.05 - 16 | Hang 2014, Rosa 2008, Billiau 2007, Kothur 2016 |
| IL-17A | 280 | 1.4 - 74 | 1 - 4 | Rosa 2008, Grant 2012, Tylman 2011, Comar 2014, Kothur 2016, Yoshio 2016 |
| TNF-α | 3 | 4 - 15 | 0 - 16 | Rosa 2008, Surendar 2012, Szary_ska 2015, Nishida 2015, Uyanik 2015, Chao 2008, Grant 2012, Hang 2014, Siregar 2015, Schnabel 2008, Quirico-Santos 2013, Nowak 2011, Kothur 2016, Yoshio 2016 |
| TNF-β | 7 | 0.6 - 5.1 | | Nishida 2015 , Kim 2009, Comar 2014 |
| VEGF | 1268 | 22 - 450 | 1.5 - 64 | Bilgici 2014 , Hang 2014, Siregar 2015, Legros 2012, Wahl 2011, Legros 2014, Kothur 2016 |
| CRP | 17490 | 2.5k – 31k | | Sayed 2015, Grant 2012, Ram 2013, Siemes 2006, Fontes 2015, Schnabel 2008 |
| ICAM-1 | 7885 | 85k – 1.25M | | Tylman 2011, Fontes 2015, Schnabel 2008, Aksoy 2015 |
| VCAM-1 | 8203 | 0.4M – 11M | | Dworacka 2014, Tylman 2011, Aksoy 2015 |

# Table S4K. REFERENCES, BLOOD MEDIATOR LEVELS

| | | |
|---|---|---|
| **Agarwal M. et al.** | 2013 | Prostate. 2013 May ; 73(6): 573–581. doi:10.1002/pros.22597. |
| **Aksoy S. et al.** | 2015 | Am. J. Phys. Med. Rehabil. & Vol. 94, No. 10, October 2015 |
| **Alapirtti T. et al.** | 2009 | Journal of the Neurological Sciences 280 (2009) 94-97 |
| **Bauer S. et al.** | 2009 | Epilepsy Research (2009) 86, 82—88 |
| **Bilgici B. et al.** | 2014 | Endocrine (2014) 45:61–66 DOI 10.1007/s12020-013-9945-7 |
| **Billiau A.D. et al.** | 2007 | *Epilepsia, Vol. 48, No. 9, 2007* |
| **Breland U. et al.** | 2010 | Cardiovascular Research (2010) 86, 265–273 doi:10.1093/cvr/cvq044 |
| **Chao P-c et al.** | 2008 | European Journal of Nutrition Oct 2010, 49(7): 429-434 |
| **Comar M. et al.** | 2014 | PLoS One. 2014; 9(8): e104848. Pub online 2014 Aug 27. doi: 10.1371/journal.pone.0104848 |
| **Dworacka M. et al.** | 2014 | European Journal of Pharmacology 740(2014): 474–479 |
| **Feng S. et al.** | 2015 | BMC Pediatrics (2015) 15:109 DOI 10.1186/s12887-015-0424-6 |
| **Flammand L. et al.** | 2001 | LTB4 Induces Release of a-Defensins • JID 2004:189 (1 June) • 2003 |
| **Floris-Moore M. et al.** | 2009 | AIDS. 2009 May 15; 23(8): 941–949. doi: 10.1097/QAD.0b013e328329c76b |
| **Fontes J.D. et al.** | 2015 | Atherosclerosis 240 (2015) 431-436 |
| **Garcia-Alonso M. et al.** | 2009 | Journal of Nutritional Biochemistry 20 (2009) 521–529 |
| **Grant P.M. et al.** | 2012 | J Infect Dis. 2012 Dec 1; 206(11): 1715–1723. 2012 Sep 21. doi: 10.1093/infdis/jis604 |
| **Hang H. et al.** | 2014 | Mol Vis. 2014; 20: 1137–1145. |
| **Hashimoto I. et al.** | 2006 | Obesity Hashimoto 2009 Volume 14, Issue 5, pages 799-811, 6 SEP 2012 DOI: 10.1038/oby.2006.93 http://onlinelibrary.wiley.com/doi/10.1038/oby.2006.93/full#f2 |
| **Heidigger I. et al.** | 2015 | The Prostate vol. 75, Issue 16, pages 1904–1909, December 1, 2015 |
| **I. M. El-Kady et al.** | 2005 | Scandinavian Journal of Immunology 61, 87–91 (2005) |
| **Ichinose K. et al.** | 2015 | Clinical Immunology (2015) 157, 114–120 |
| **Ichiyama T. et al.** | 2008 | Brain & Development 30 (2008) 47–52 |
| **Jung C. et al.** | 2010 | Mediators Inflamm. 2010;2010:958403. doi: 10.1155/2010/958403. |
| **Karamehic J. et al.** | 2008 | Med Arh. 2008;62(3):136-8. |
| **Kim J-G. et al.** | 2009 | Menopause 16(3): 534-538, 2009. DOI: 10.1097/gme.0b013e3181920c77 |
| **Kothur K. et al.** | 2016 | Cytokine 77 (2016) 227–237 |
| **Legros L. et al.** | 2012 | Ann Hematol. 2012 Jan;91(1):39-46. doi: 10.1007/s00277-011-1242-z. Epub 2011 May 7. |
| **Miyazaki E. et al.** | 2007 | CHEST 2007; 131(6):1726–1734 |
| **Navarro-Sobrino M. et al.** | 2009 | Current Neurovascular Research 6(4): 246-251 |
| **Nishida Y. et al.** | 2015 | Archives of Gerontology and Geriatrics 61 (2015) 443–451 |
| **Nowak M. et al.** | 2011 | Brain, Behavior, and Immunity 25 (2011) 423–428 |
| **Pawlak K. et al.** | 2006 | Cytokine 35 (2006) 258–262 |
| **Petrikis P. et al.** | 2015 | Journal of Psychosomatic Research 79 (2015) 324–327 |
| **Purohit S. et al.** | 2015 | J Clin Endocrinol Metab, September 2015, 100(9):E1179–E1187 http://dx.doi.org/10.1210/JC.2015-1388#sthash.YZOKoguD.dpuf |
| **Quirico-Santos T. et al.** | 2013 | Journal of Neuroimmunology 254 (2013) 125–130 |
| **Ram R. et al.** | 2013 | Journal of Infection 2013, 67(3): 194–198 |
| **Resende R. et al.** | 2012 | Oral Diseases (2012) 18, 816–822 doi:10.1111/j.1601-0825.2012.01955.x |
| **Romagnani P. et al.** | 2002 | American Journal of Pathology, Vol. 161, No. 1, July 2002 |
| **Rosa J.S. et al.** | 2008 | Pediatric Diabetes 2008: 9: 9–16 |
| **Sayed S. et al.** | 2015 | British Journal of Biomedical Science, 72:3, 93-101, DOI: 10.1080/09674845.2015.11666803 |
| **Schnabel R. et al.** | 2008 | Hypertension. 2008; 51:1651-1657 |
| **Shiah I. et al.** | 2005 | International Clinical Psychopharmacology 2005, 20:295–298 |
| **Siemes C. et al.** | 2006 | J Clin Oncol. 2006 Nov 20;24(33):5216-22. |
| **Simmons R. et al.** | 2013 | AIDS. 2013 October 23; 27(16): 2505–2517. doi:10.1097/01.aids.0000432455.06476.bc. |
| **Siregar G. et al.** | 2015 | Acta Med Indones. 2015 Apr;47(2):120-6. |
| **Surendar J. et al.** | 2012 | DIABETES TECHNOLOGY & THERAPEUTICS (2012) 14(4): 344-349. DOI: 10.1089/dia.2011.0182 |
| **Szaryńska M. et al.** | 2015 | Cellular Immunology 293 (2015) 137–141 |
| **Terpos E. et al.** | 2005 | Leukemia & Lymphoma 46, (12), 2005, 1699-1707 DOI: 10.1080/10428190500175049 |
| **Tylman M. et al.** | 2011 | Minerva Anestesiol. 2011 Mar;77(3):275-82. Epub 2010 Dec 9. |
| **Uludag I.F. et al.** | 2013 | Seizure 22 (2013):457–461 |
| **Uyanik V. et al.** | 2015 | Psychiatry Research 228 (2015) 386–392 |
| **Wahl P. et al.** | 2011 | Eur J Appl Physiol (2011) 111:1405–1413 |
| **Xu H. et al.** | 2005 | Nihon Jinzo Gakkai Shi. 2005;47(5):524-30. |
| **Yoshio T. et al.** | 2016 | Lupus (2016 Feb3) 0, 1–7 DOI:10.1177/0961203316629556 |
| **Zhang F. et al.** | 2015 | BMC Infect Dis. 2015; 15: 457. |
| **Zinyama-Gutsire R. et al.** | 2009 | BMC Infect Dis. 2009; 9: 174. doi: 10.1186/1471-2334-9-174 |

# 4A. Age-Related Correlations

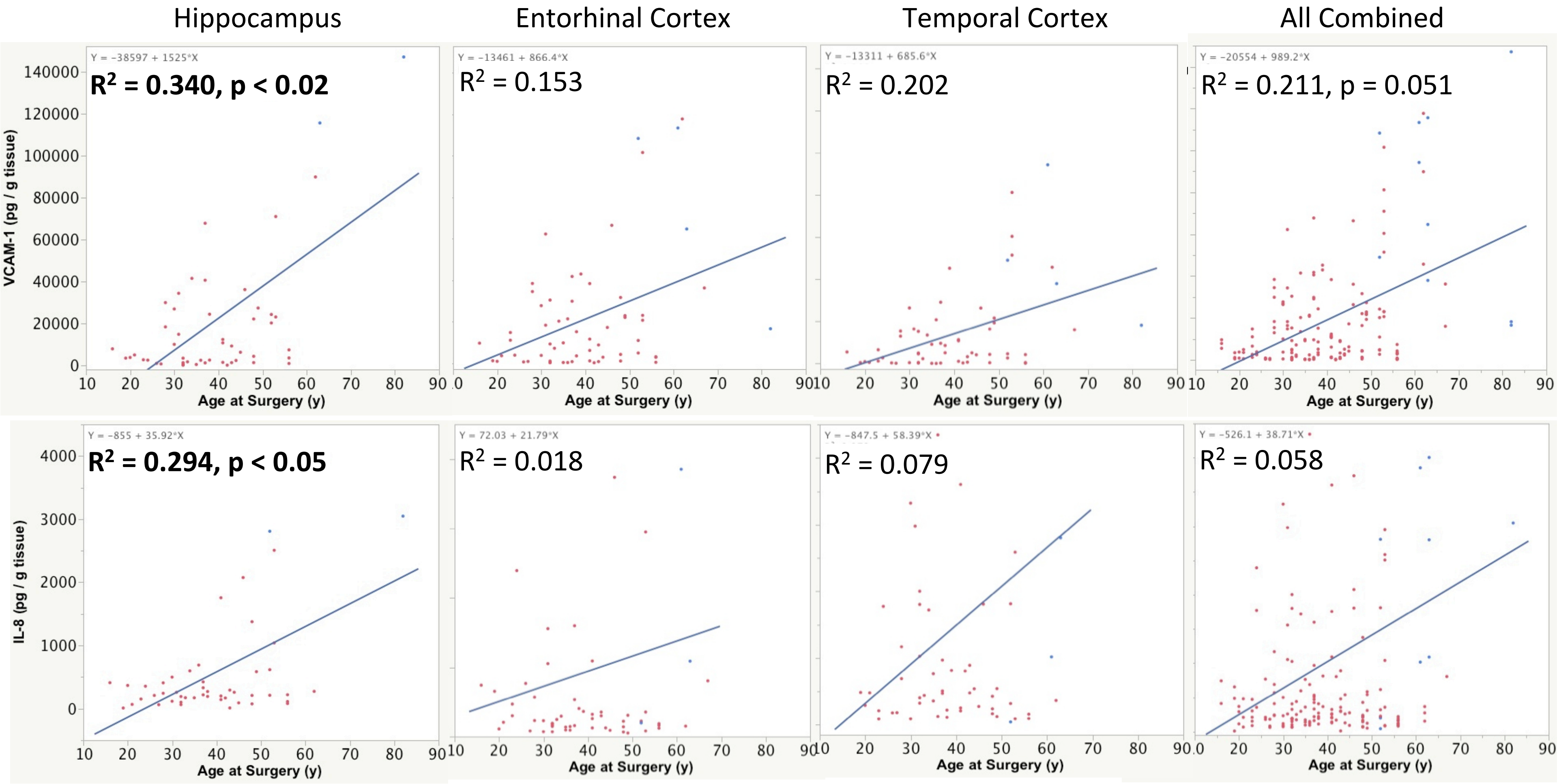

# 4B. Epilepsy Risk Factor Associations

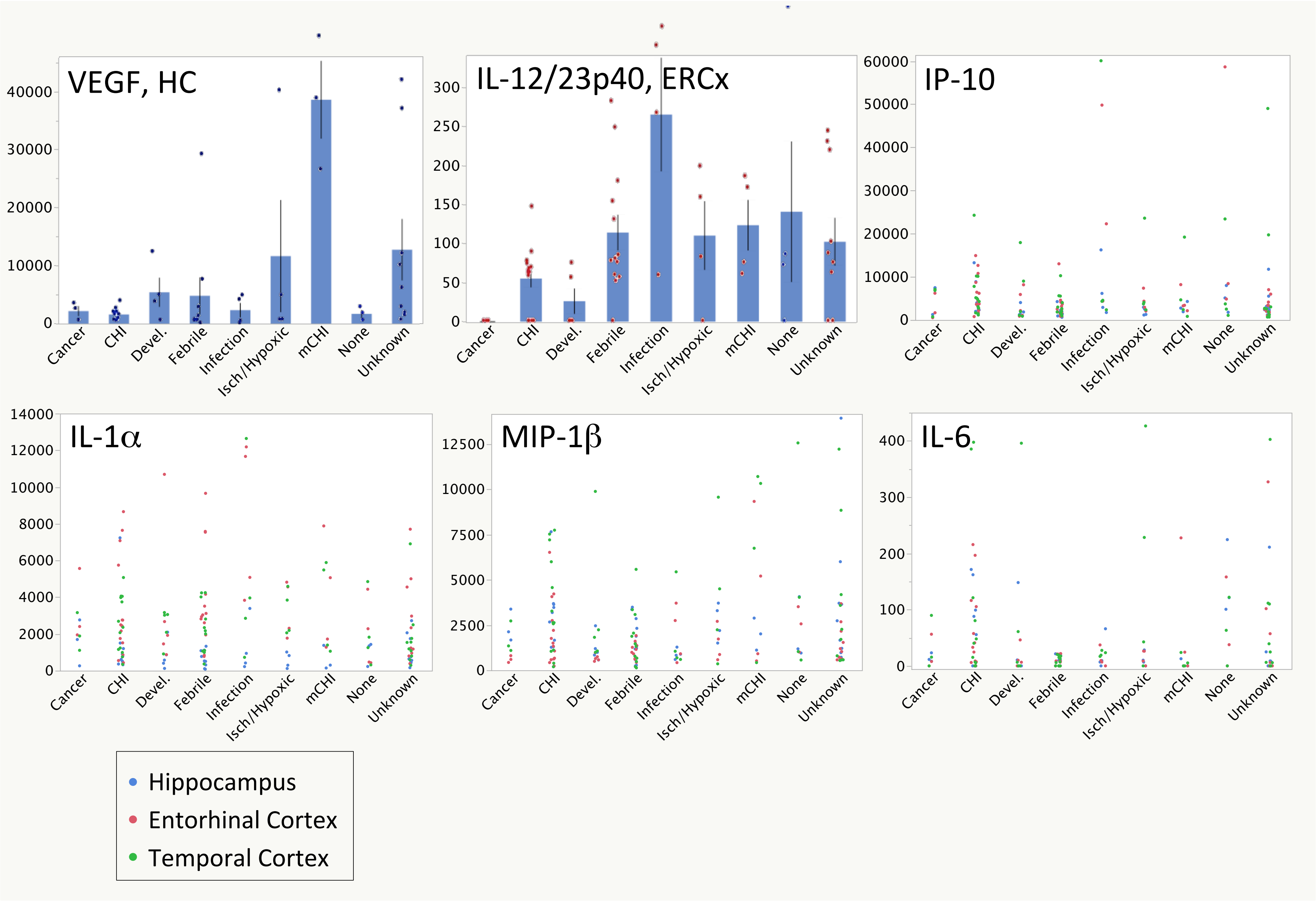

## 4C. Relational Correlations Between Tissues

Boxes and horizontal lines indicate cross-tissue correlations, *italics* show connections between relational groups.

| | | | | | | | |
|---|---|---|---|---|---|---|---|
| Eotaxin_HC | MCP-4_HC | TARC_HC | *CRP_HC* | | | | |
| Eotaxin_ERCx | MCP-4_ERCx | TARC_ERCx | Eotaxin_ERCx | | | IL-12/23p40_HC | |
| *VCAM-1_TCx* | *VCAM-1_ERCx* | *VCAM-1_ERCx* | | TARC_TCx | IL-12/23p40_TCx | VEGF_ERCx | |
| *VCAM-1_ERCx* | *VCAM-1_HC* | *VCAM-1_HC* | | | | VEGF_TCx | |
| *IL-8_HC* | *IL-6_HC* | | | MCP-1_HC | IL-12/23p40_TCx | | |
| | *IL-8_HC* | | | | | | |

| | | | | | | | | | | | | |
|---|---|---|---|---|---|---|---|---|---|---|---|---|
| | | | | | | | | | | | *IL-8_HC* | |
| | | | | | | | | | | | *IL-6_HC* | |
| Eotaxin_HC | ICAM-1_HC | CRP_HC | IL-12p70:p40_HC | | | | | | CRP_HC | | CRP_TCx | |
| Eotaxin_ERCx | ICAM-1_ERCx | CRP_ERCx | MCP-1_ERCx | MIP-1α_ERCx | MIP-1β_ERCxª | *MCP-4_ERCx* | CRP_ERCx | | ICAM-1_ERCx | IL-17A_ERCx | MCP-1_ERCx | |
| Eotaxin_TCx | ICAM-1_TCx | CRP_TCx | MCP-1_TCx | MIP-1α_TCx | MIP-1β_TCx | MCP-4_TCxª | Eotaxin_TCx | IP-10_TCx | ICAM-1_TCx | IL-17A_TCx | MCP-1_TCx | *IL-10_TCx* |
| *IL-2_TCx* | | ICAM-1_ERCx | *VCAM-1_TCx* | | | | | IP-10_HC | | | *IL-6_HC* | |
| *IL-4_TCx* | | | | | | | | IL-12p70:p40_HC | | | | |

ªNot_directly_corr_to_Eotaxin_TCx

| | | | | | | |
|---|---|---|---|---|---|---|
| MIP-1α_HC | MIP-1β_HC | | *IL-8_HC* | MIP-1α_HC | IL-1α_HC | |
| MIP-1α_ERCx | MIP-1β_ERCx | IL-1β_ERCx | MIP-1α_ERCx | IP-10_ERCx | | IL-1α_ERCx |
| MIP-1α_TCx | MIP-1β_TCx | TNF-α_TCx | MIP-1β_TCx | | | IL-1α_TCx |
| | | | *IL-2_TCx* | | | |
| | | | *IL-4_TCx* | | | |

| | | | | | | | | |
|---|---|---|---|---|---|---|---|---|
| IL-1β_HC* | IL-6_HC | IL-8_HC | | | | | IL-6_HC,_IL8_HC | VCAM-1_HC |
| IL-1β_ERCx | IL-6_ERCx | IL-8_ERCx | TNF-α_ERCx | IL-1β_ERCx | IP-10_ERCx | TNF-α_ERCx | IL-6_HC,_IL8_HC | VCAM-1_ERCx |
| IL-1β_TCx | IL-6_TCx | IL-8_TCx | TNF-α_TCx | IL-10_TCx | | TNF-β_HC | IL-6_HC,_IL8_HC | VCAM-1_TCx |
| | *IL-2_TCx* | | IL-8_TCx | IL-10_TCx | | | | |

*not_correlated_to_IL-8_HC

**Negative Correlations:**

| | |
|---|---|
| IL-12p70:p40_ | CRP_HC |
| TNF-α_ERCx | TNF-β_HC |

| | |
|---|---|
| IFN-γ_HC | IL-2_HC |
| IFN-γ_HC | IL-4_HC |
| IFN-γ_HC | MCP-4_HC |
| IFN-γ_HC | TARC_HC |
| IFN-γ_TCx | IL-8_ERCx |
| GM-CSF_HC | IL-10_HC |
| GM-CSF_ERCx | IL-6_HC |

| | | | | | | | | | |
|---|---|---|---|---|---|---|---|---|---|
| IL-4_HC | IL-12p70_HC | TNF-β_HC | | | IL-2_HC | | | | |
| IL-4_ERCx | IL-12p70_ERCx | IL-1β_ERCx | TNF-α_ERCx | IP-10_ERCx | | IL-2_ERCx | IL-17A_ERCx | IP-10_ERCx | |
| IL-4_TCx | IL-12p70_TCx | IL-1β_TCx | TNF-α_TCx | IL-4_TCx | IL-2_TCx | IL-1β_TCx | | IL-2_TCx | IL-8_TCx |
| | | | | | | | | *VCAM-1_HC* | |

| # CORRELATIONS | Hippocampus | Entorhinal cortex | Temporal cortex |
|---|---|---|---|
| NONE: | IL-1α, IL-17A, IL-10, TNF-α, GM-CSF | IL-10, IL-12 p70, IL-12/23 p40, GM-CSF | IL-12 p70 GM-CSF |
| ONE: | MCP-1 ≈ IL-12/23P40_TCx<br>IL-1β ≈ IL-6_HC<br>IL-12/23p40 ≈ VEGF_ERCx<br>MIP-1β ≈ MIP-1a_HC<br>IP-10 ≈ IP-10_TCx | - | MCP-4 ≈ ICAM-1_TCx<br>TARC ≈ IL-12/23p40_TCx |

"≈" indicates a significant correlation

| | |
|---|---|
| GM-CSF | (2) |
| IL-1α | (2) |
| TNF-β | (3) |
| VEGF | (4) |
| IFN-γ | (6) |
